# Rosuvastatin Attenuates CD40L-Induced Downregulation of Extracellular Matrix Production in Human Aortic Smooth Muscle Cells via TRAF6-JNK-NF-κB Pathway

**DOI:** 10.1371/journal.pone.0153919

**Published:** 2016-04-27

**Authors:** Xiao-Lin Wang, Yuan-Li Zhou, Wei Sun, Li Li

**Affiliations:** 1 Department of Cardiology, Jinan Central Hospital affiliated to Shandong University, Jinan, Shandong, 250013, P.R. China; 2 Department of Health, Jinan Central Hospital affiliated to Shandong University, Jinan, Shandong, 250013, P.R. China; 3 Department of Cardiology, Shandong Academy of Medical Sciences, Jinan, Shandong, 250062, P.R. China; Max-Delbrück Center for Molecular Medicine (MDC), GERMANY

## Abstract

CD40L and statins exhibit pro-inflammatory and anti-inflammatory effects, respectively. They are both pleiotropic and can regulate extracellular matrix (ECM) degeneration in an atherosclerotic plaque. Statins can decrease both the CD40 expression and the resulting inflammation. However, the effects of CD40L and stains on atherosclerotic plaque ECM production and the underlying mechanisms are not well established. Moreover, prolyl-4-hydroxylase α1 (P4Hα1) is involved in collagen synthesis but its correlations with CD40L and statins are unknown. In the present study, CD40L suppressed P4Hα1 expression in human aortic smooth muscle cells (HASMCs) in a dose- and time-dependent manner, with insignificant changes in MMP2 expression and negative enzymatic activity of MMP9. CD40L increased TRAF6 expression, JNK phosphorylation, NF-κB nuclear translocation as well as DNA binding. Furthermore, silencing TRAF6, JNK or NF-κB genes abolished CD40L-induced suppression of P4Hα1. Lower NF-κB nuclear import rates were observed when JNK or TRAF6 silenced HASMCs were stimulated with CD40L compared to HASMCs with active JNK or TRAF6. Together, these results indicate that CD40L suppresses P4Hα1 expression in HASMCs by activating the TRAF6-JNK- NF-κB pathway. We also found that rosuvastatin inhibits CD40L-induced activation of the TRAF6-JNK- NF-κB pathway, thereby significantly rescuing the CD40L stimulated P4Hα1 inhibition. The results from this study will help find potential targets for stabilizing vulnerable atherosclerotic plaques.

## Introduction

Atherosclerotic plaque rupture is a key event contributing to the pathogenesis of acute coronary syndrome. The risk of atherosclerotic plaque rupture depends mostly on plaque composition and vulnerability [1]. Characteristic histomorphological features of vulnerable plaques include a large lipid core and a thin fibrous cap [2]. Type 1 and type 3 collagen synthesized by human aortic smooth muscle cells (HASMCs) are the main components of the extracellular matrix (ECM) in arterial wall/plaque. A major portion of the fibrous cap is made up of these collagens that provide strength and integrity to the fibrous cap as well as maintain plaque stability [3]. Prolyl-4-hydroxylase (P4H) is a key enzyme in collagen biosynthesis, where the subunit P4Hα1 catalyzes the posttranslational processing of collagen synthesis in most cell types and tissues[4]. Matrix metalloproteinases (MMPs) are a family of enzymes that degrade ECM components of the atherosclerotic plaque, thereby inducing plaque instability. MMPs are mainly produced by macrophages and HASMCs of the atherosclerotic plaque, and are known to play a role in balancing the collagen homeostasis [5–7]. MMP2 and MMP9 belong to a sub-group of gelatinases that share similar proteolytic activity, and degrade denatured collagens, gelatins and various ECM components, thereby playing an important role in atherosclerotic plaque rupture [8–10].

The CD40 ligand (CD40L) and its receptor, CD40, belong to the tumor necrosis factor (TNF) family and tumor necrosis factor receptor (TNF-R) family, respectively [11]. It is well established that they are involved in immune regulation, inflammation and in plaque instability, and are not restricted to T- and B-lymphocytes [12–15]. CD40 and CD40L are expressed on the macrophages, endothelial cells and SMCs in the atherosclerotic plaque. [16] The soluble form of CD40L (sCD40L) is an 18-kDa protein comprising the entire TNF homologous region of CD40L. It is generated *in vivo* by intracellular proteolytic processing of the full length CD40L. Recombinant human soluble CD40 ligand (rhsCD40L) is a 16.3-kDa protein containing 149 amino acid residues and comprises the receptor binding TNF-like domain of CD40L. Previous studies have reported that CD40L upregulates MMPs in the atherosclerotic plaque, which eventually leads to increased collagen degradation and plaque instability [17–19]. The present study aims to identify the dual role of CD40L in the regulation of P4Hα1 and MMPs, 2 important proteins involved in collagen homeostasis.

TNF receptor-associated factors (TRAFs) are major signaling mediators downstream of CD40 [20] and particularly, TRAF6 has been shown to play an important role in promoting atherosclerosis [21–22]. CD40-mediated signal transduction differs significantly depending on the cell type, function, origin, differentiation stage and activation status [12, 13, 23, 24]. Mitogen-activated protein kinase (MAPK) pathway is one of the most studied controversial signaling pathway [24]. Extracellular signal-regulated kinase (ERK), C-jun N-terminal kinase (JNK) and p38 MAPK are the 3 key pro-inflammatory MAPKs, and are involved in collagen turnover and the development of atherosclerotic lesions [25]. Nuclear factor kappa-light-chain-enhancer of activated B cells (NF-κB) is activated via TRAF-mediated MAPKs and/or by TRAFs itself via CD40 signaling [24]. NF-κB can control the expression of genes that are involved in the initiation and progression of atherosclerosis [26]. The current study examines the relationship between CD40L-stimulated P4Hα1 and MMP expression, as well as the TRAF6-MAPKs-NF-κB signaling pathway in HASMCs. The results from this study will aid in finding new targets for stabilizing vulnerable atherosclerotic plaques.

HMG-CoA reductase inhibitors (statins) are widely prescribed as potent lipid lowering agents. It has been previously demonstrated that statins being pleiotropic can exert cholesterol-independent effects in atherosclerosis by reducing inflammation and stabilizing the plaque [27, 28]. Previous studies have found that statins decrease CD40 expression [29, 30] and the resulting inflammation [31], and also inhibit ECM degradation of the atherosclerotic plaques [32–34]. However, the direct effects of rosuvastatin—a statin—on CD40-induced P4Hα1 expression and collagen production are unknown. We therefore investigated whether rosuvastatin regulates ECM metabolism in CD40L-stimulated HASMCs, and if so, the possible regulatory mechanisms.

## Materials and Methods

### 1. Cell culture and treatment

HASMCs were purchased from American Type Cell Collection (Manassas, VA, USA) and cultured in Smooth Muscle Cell Medium (Cat#1101, ScienCell, Carlsbad, CA, USA) containing 2% fetal bovine serum. Cells were cultured up to the 6^th^ passage in a humidified incubator at 37°C, 5% CO_2_. Experiments were performed with 80%-90% confluent cells after rendering them quiescent by serum-free starvation for 24 h.

To study the dose- and time- dependent effects of CD40L stimulation on P4Hα1 expression, HASMCs were treated with 0, 2, 5, and 10 μg/ml of recombinant human sCD40-Ligand (rhsCD40L; PeproTech, Rocky Hill, NJ, USA) for 8 h, and with 5 μg/ml rhsCD40L for 0, 4, 8 and 12 h.

HASMCs were treated with 5 μg/ml rhsCD40L for 0, 15, 30, 60 and 120 min, following which the NF-κB subcellular localization and NF-κB DNA binding were assayed to explore the role of CD40L-induced NF-κB on P4Hα1 expression. To investigate the role of TRAF6-MAPKs pathway in CD40L-induced suppression of P4Hα1, HASMCs were treated with 5 μg/ml rhsCD40L for 0, 5, 15, 30 and 120 min or 5 μg/ml rhsCD40L for 10 min, following which the expression of TRAF6 or phosphorylation levels of ERK1/2, JNK and p38 MAPK were analyzed respectively.

The role of the TRAF6-MAPKs-NF-κB pathway was further verified by gene silencing. HASMCs were transfected with control siRNA (Cat#6201), TRAF6 siRNA (Cat#sc-36717, Santa Cruz, Dallas, TX, USA; 100 nM), ERK1/2 siRNA (Cat#6560), JNK siRNA (Cat#6232), p38 MAPK siRNA (Cat#6564) or NF-κB siRNA (Cat#6261) (all Cell Signaling Technology, Danvers, MA, USA; all 100 nM) for 48 h using MACSfectin Reagent (Cat#130098410, Miltenyi, Germany), and then incubated with 5μg/ml rhsCD40L for additional 8 h.

To study the effects of rosuvastatin, HASMCs were pretreated with or without 10 μM rosuvastatin calcium salt (Cat#sc-208316, Santa Cruz) for 2 h before rhsCD40L stimulation (statin was still included in the stimulation medium). This particular dose was selected because it corresponds to the highest therapeutic dose (40 mg) found in the patients’ blood treated with rosuvastatin [35].

### 2. Quantitative real-time reverse-transcriptase-PCR (qRT-PCR)

Total RNA was extracted from HASMCs using TRIzol (Invitrogen, Carlsbad, CA, USA) following the manufacturer’s protocol, and quantified by spectrophotometry. The cDNA was reverse transcribed in a Bio-Rad thermocycler using the RT reagent kit (Cat#RR047A, TaKaRa, Japan). Real-time PCR was then performed with a SYBR Green I Premix kit (Cat#RR820A, TaKaRa) in a 7500 Real-Time PCR System (Applied Biosystems, Foster City, CA, USA) under the following conditions: 40 cycles at 95°C for 5 seconds and 60°C for 34 seconds. Sequence-specific primers used were as follows:

GAPDH  (Fw) 5’- GGAGCGAGATCCCTCCAAAAT

               (Rev) 5’- GGCTGTTGTCATACTTCTCATGG

P4Hα1    (Fw) 5’- CATGACCCTGAGACTGGAAA

                Rev) 5’- GCCAGGCACTCTTAGATACT

MMP2    (Fw) 5’- ACTGTGACGCCACGTGAACAA

                (Rev) 5’- CGTATACCGCATCAATCTTTTCC

MMP9    (Fw) 5’- CCTGGAGACCTGAGAACCAATC

                (Rev) 5’- GATTTCGACTCTCCACGCATC

TRAF6    (Fw) 5’- CCAATTCCATGCACATTCAG

                (Rev) 5’- GGGCCAACATTCTCATGTGT

The relative mRNA expression was normalized to GAPDH and analyzed using the 2^−ΔΔCT^ method. All the experiments were repeated at least 3 times.

### 3. Western blot analysis

The HASMCs and corresponding supernatant were collected, lysed and concentrated (×10) for protein extraction. Phosphatase inhibitor cocktail (Cat#5870, CST) was added to RIPA lysis buffer supplemented with 1mM PMSF for detection of the phosphorylated proteins. Equal amounts of protein were separated using 10% sodium dodecyl sulfate-polyacrylamide gel electrophoresis (SDS-PAGE) and transferred to polyvinylidene difluoride membranes. The membranes were blocked for 1 h at room temperature using 5% nonfat milk, and incubated overnight at 4°C with the appropriate primary antibody. The protein in concentrated supernatant were used to detect secretory collagen. The primary antibodies used were as follows: goat polyclonal anti-P4Hα1 (Cat#ab59497; 1:500), rabbit polyclonal anti-type I collagen (Cat#ab34710; 1:5000), rabbit polyclonal anti-type III collagen (Cat#ab7778; 1:5000) (all Abcam, Cambridge, UK), rabbit monoclonal anti-phospho-ERK1/2 (Cat#4370; 1:2000), rabbit monoclonal anti-ERK1/2 (Cat#4695; 1:1000), rabbit monoclonal anti-phospho-JNK (Cat#4671; 1:1000), rabbit monoclonal anti-JNK (Cat#9258; 1:1000), rabbit monoclonal anti-phospho-p38MAPK (Cat#4511; 1:1000), rabbit monoclonal anti-p38MAPK (Cat#8690; 1:1000), and rabbit monoclonal anti-β-Actin (Cat#4970; 1:1000) (all CST). The membranes were washed 3 times for 10 min each with Tris-buffered saline containing Tween (TBS-T), and then incubated with horseradish peroxidase-conjugated secondary antibodies for 2 h at room temperature. Following additional washes, the immunoreactive proteins on the membrane were visualized with enhanced chemiluminescence plus reagents (Millipore, Plano, TX, USA). The intensities of the immunoreactive proteins were measured via computerized image analysis and normalized to β-actin. All the experiments were repeated at least 3 times.

### 4. Gelatin zymography

The gelatinolytic activities of MMP-2 and MMP-9 were evaluated by gelatin zymography using a gelatinase activity assay kit (Cat#30071, Genmed Scientific Inc, Shanghai, China). Equal volume of cell culture supernatants were electrophoresed in the SDS gel containing gelatin, and subjected to renaturation (1 h), digestion (20 h), staining (1 h), destaining (30 min) and termination by incubation in the corresponding reagents at room temperature. All the reagents were provided in the kit. The zymograms were photographed on a light box, and the latent or active MMP-2 and MMP-9 activity was detected as a translucent area over a dark blue background. All the experiments were repeated at least 3 times.

### 5. Electrophoretic mobility shift assay (EMSA) analysis of NF-κB

Nuclear proteins were isolated from cultured HASMCs treated with or without rhsCD40L using a NucBuster Protein Extraction Kit (Cat#71183, Merck Millipore, Darmstadt, Germany) following the manufacturer’s protocol. EMSA was performed with a DIG Gel Shift Kit (Cat#03353591910, Roche, Madison, WI, USA) using the DIG-endlabeling technique to detect sequence-specific NF-κB-binding proteins. Briefly, NF-κB DNA fragment sense strand oligonucleotides (5'-AGTTGAGGGGACTTTCCCAGGC-3') were annealed with their antisense strands and labeled with digoxigenin-11-ddUTP at the 3’-ends. The labeled probes were incubated with the extracted nuclear proteins, which allowed the formation of DNA-protein complexes. The mixture was run on a 6% native polyacrylamide gel at 80 V and blotted on a positively charged nylon membrane (Cat#INYC00010, Millipore). The membrane was UV-cross linked, blocked, and then incubated with anti-Digoxigenin-AP. Chemiluminescent detection was performed with CSPD and recorded on an imaging device. All the experiments were repeated at least 3 times.

### 6. Immunofluorescence

All the slides containing cells were fixed in 4% paraformaldehyde for 15 min, permeated in 0.1% Triton X-100 for 5 min, and then blocked in 5% goat serum for 1 h. HASMCs were stained or costained with rabbit anti-TRAF6 (Cat#ab62488, Abcam; 1:50) and mouse anti-NF-κB (Cat#sc8008, Santa Cruz; 1:50) overnight at 4°C. The cells were incubated with secondary goat anti-rabbit IgG-PE conjugate (Cat#sc3739, Santa Cruz; 1:100) or goat anti-mouse IgG-FITC conjugate (Cat#sc2010, Santa Cruz; 1:100). The slides were washed 3 times in between each step, and lastly, incubated with anti-fade reagent with DAPI (Cat#8961, CST, USA). The cells were observed under a confocal laser scanning microscopy (LSM710, ZEISS, Germany). We took 4 images for every slide randomly, and repeated all the experiments for at least 3 times.

### 7. Statistical analysis

Statistical analysis was performed using the SPSS software (version 20.0, SPSS, Chicago, IL, USA) for Windows. Data was presented as Mean ± SD. The differences between 2 groups were analyzed by independent-samples *t* test. For more than 2 groups, the differences were calculated by one-way ANOVA with LSD post hoc test. All the experiments were repeated at least 3 times. P < 0.05 was considered statistically significant.

## Results

### 1. Effect of CD40L and rosuvastatin on the expression of P4Hα1 and gelatinases in HASMCs

#### 1.1. Dose- and time-dependent effect of CD40L on the expression of P4Hα1 and gelatinases

**Fig 1:**
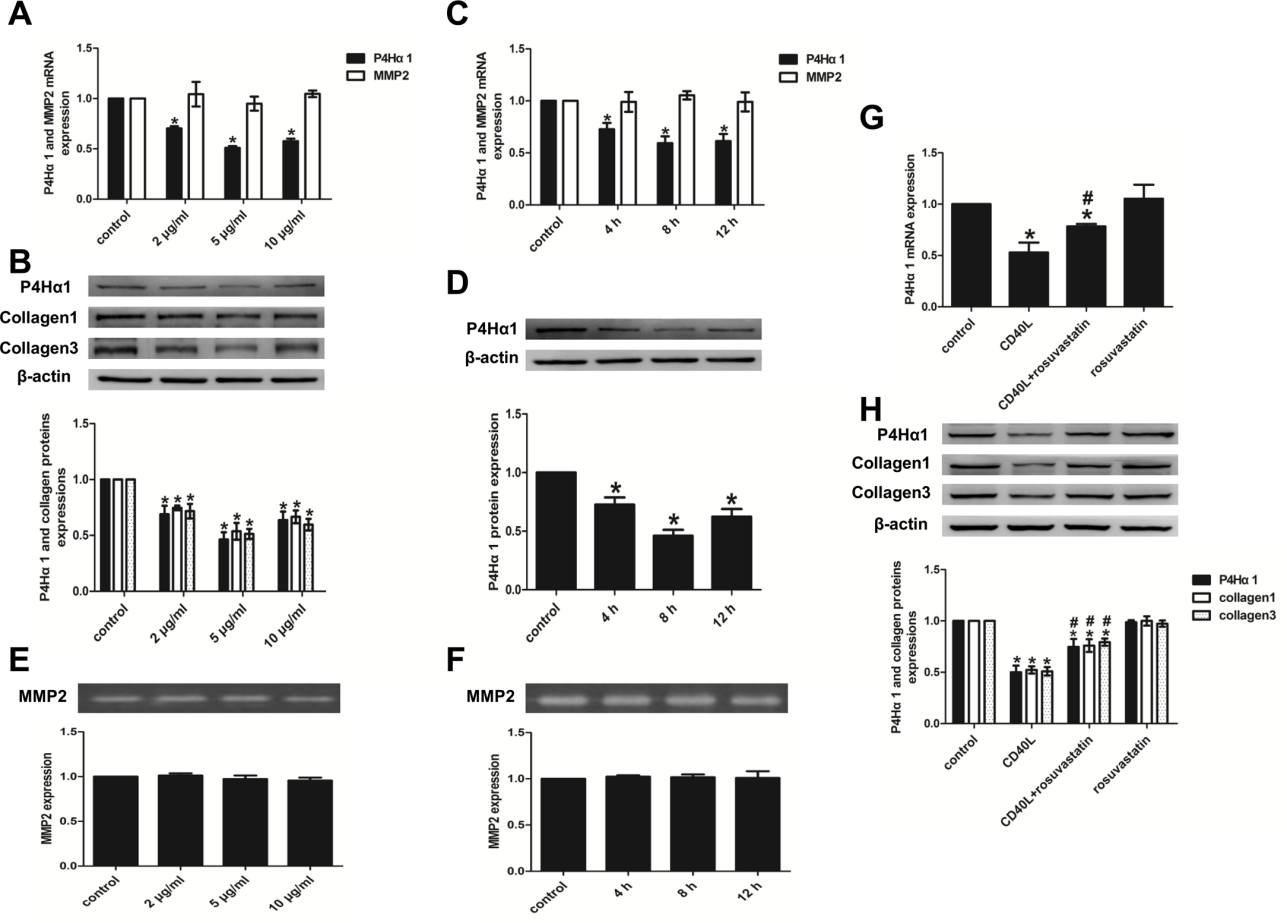
Effect of CD40L and rosuvastatin on the expression of P4Hα1 and gelatinases in HASMCs. (A, B and E) MMP2, P4Hα1 and collagen expression after treated with 0, 2, 5, and 10 μg/ml rhsCD40L for 8 h by qRT-PCR (A), western blot analysis (B) and gelatin zymography (E). (C, D and F) MMP2 and P4Hα1 expression after treated with 5 μg/ml rhsCD40L for 0, 4, 8 and 12 h by qRT-PCR (C), western blot analysis (D) and gelatin zymography (F). (G and H) P4Hα1 and collagen expression after treated with or without rhsCD40L and rousuvastatin by qRT-PCR (G) and western blot analysis (H). ^*^P < 0.05 vs. control, ^#^*P* <0.05 vs. CD40L.

To study the dose-dependent effects of CD40L stimulation on P4Hα1 expression, HASMCs were treated with 0, 2, 5, and 10 μg/ml rhsCD40L for 8 h, following which the RNA and protein were extracted for qRT-PCR analysis and western blotting, respectively. The analysis revealed that CD40L significantly suppressed both the P4Hα1 mRNA (Fig 1A) and protein expression (Fig 1B). The suppression was strongest at 5 μg/ml, and the P4Hα1 protein expression corresponded with the inhibition of type 1 and 3 collagens (Fig 1B). For the time-dependent study, HASMCs were treated with 5 μg/ml rhsCD40L for 0, 4, 8 and 12 h. The data revealed that CD40L downregulated P4Hα1 mRNA and protein expression, where P4Hα1 reached a plateau after 8 hours of stimulation (Fig 1C and 1D).

Additional studies were performed to analyze the effect of CD40L stimulation on the expression of MMP2 and MMP9 using qRT-PCR and gelatin zymography. The results indicated that CD40L does not affect the MMP2 mRNA expression (Fig 1A and 1C) and MMP2 enzymatic activity (Fig 1E and 1F) at any of the indicated doses and time points, and it had no effects on MMP9 enzymatic activity.

#### 1.2. Rosuvastatin attenuates the suppressive effect of CD40L on P4Hα1 expression in HASMCs

In order to investigate the effects of rosuvastatin, HASMCs were pretreated with or without 10 μM rosuvastatin for 2 h, and then stimulated with 5 μg/ml rhsCD40L for additional 8 h. The analysis revealed that pretreatment with rosuvastatin followed by CD40L stimulation significantly rescued the CD40L stimulated P4Hα1 mRNA inhibition (Fig 1G) and protein expression with a concomitant increase in both type 1 and 3 collagen (Fig 1H). Moreover, CD40L-stimulated HASMCs expressed significantly lower P4Hα1 and collagen (both type 1 and 3) compared to control cells, which is consistent with results mentioned above. Interestingly, rosuvastatin treatment alone had no effect on the expression of P4Hα1 or collagen (Fig 1G and 1H).

### 2. NF-κB regulates the effect of rosuvastatin’s rescue of the CD40L stimulated P4Hα1 inhibition in CD40L-stimulated HASMCs

#### 2.1. CD40L activates NF-κB in HASMCs

**Fig 2:**
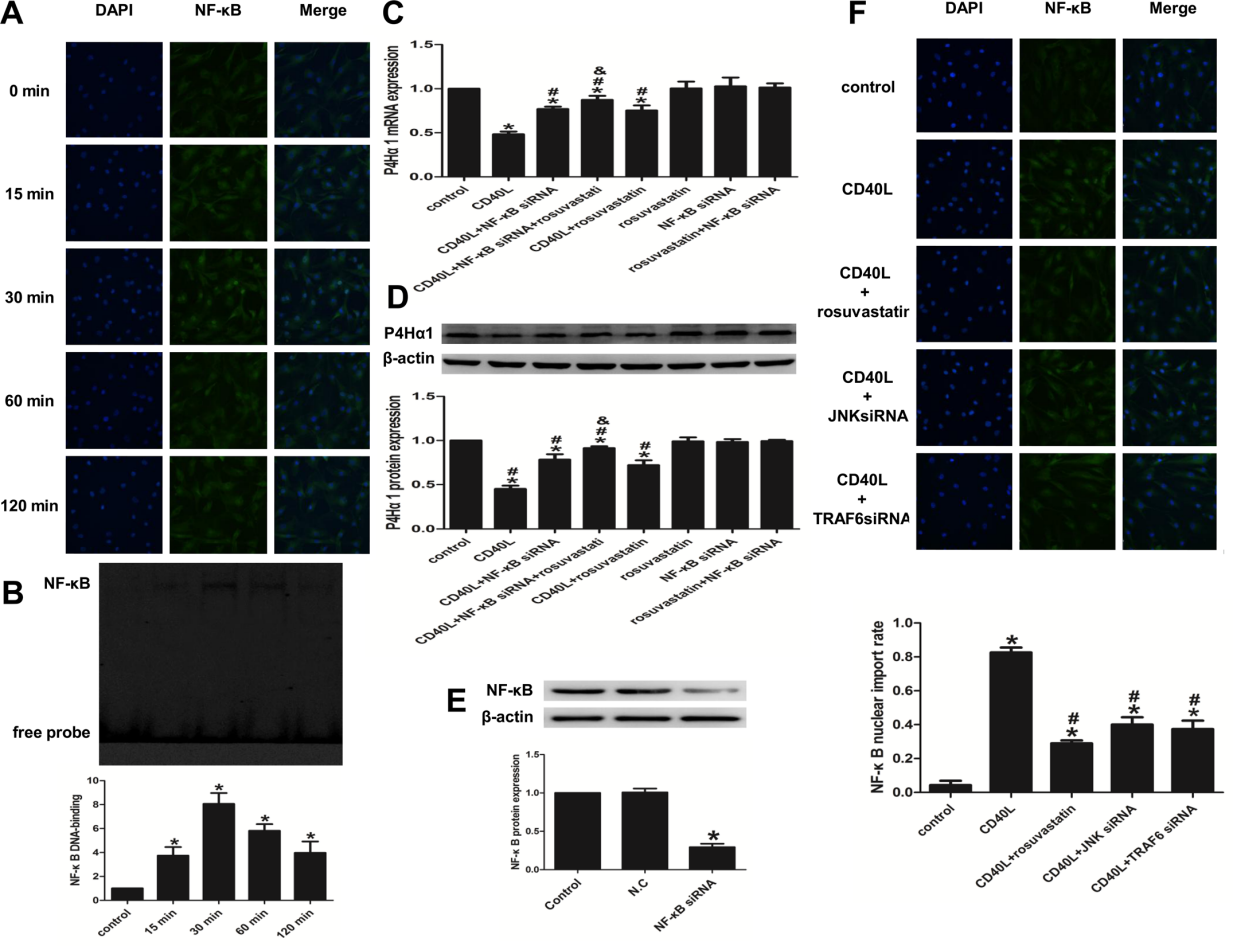
NF-κB regulates the effect of rosuvastatin’s rescue of the CD40L stimulated P4Hα1 inhibition in CD40L-stimulated HASMCs. (A and B) Representative images of NF-κb nuclear translocation and NF-κB DNA binding activity after treated with 5 μg/ml rhsCD40L for 0, 15, 30, 60 and 120 min by immunofluorescence (A) and EMSA (B). (C and D) P4Hα1 expression in HASMCs treated with or without rhsCD40L, NF-κB siRNA and rousuvastatin by qRT-PCR (C) and western blot analysis (D). (E) NF-κB protein expression in HASMCs transfected with or without NF-κB siRNA and negative control siRNA. (F) Representative images and quantitative analysis of NF-κB nuclear import rate after treated with or without rhsCD40L, rousuvastatin, JNK siRNA and TRAF6 siRNA. ^*^P < 0.05 vs. control, ^#^*P* <0.05 vs. CD40L, ^&^*P*<0.05 vs. CD40L+siRNA. N.C: the group transfected with negative control siRNA.

Time-dependent nuclear translocation of NF-κB was demonstrated by confocal microscopy after stimulation of HASMCs with 5μg/ml rhsCD40L for 0, 15, 30, 60, and 120 min. Under normal growth conditions, most unstimulated cells showed diffused staining in the cytoplasm and a weak staining in the nucleus. After stimulation with 5 μg/ml rhsCD40L for 15 min, a considerable fraction of NF-κB accumulated in the nucleus. NF-κB is maximally concentrated in the nucleus at 30 min after rhsCD40L exposure (Fig 2A). EMSA was performed to demonstrate the time course for nuclear NF-kB mobilization. The analysis revealed that CD40L significantly upregulated the NF-κB binding activity in HASMCs compared to the control cells, where it reached a peak at 30 min (Fig 2B). Collectively, the data indicated that 5 μg/ml CD40L is enough to strongly activate NF-κB at 30 min post-stimulation.

#### 2.2. Rosuvastatin attenuates the suppressive effect of CD40L on P4Hα1 expression via NF-κB

To study the downstream signaling effects of rosuvastatin, HASMCs were transfected with or without 100 nM NF-κB siRNAs for 48 h, and then pretreated with 10 μM rosuvastatin for 2 h. Following this, the cells were stimulated with 5 μg/ml rhsCD40L for 8 h. The results demonstrated that the transfected HASMCs had significantly higher P4Hα1 mRNA and protein expression compared with the non-transfected HASMCs. The NF-κB gene silenced HASMCs that had been pretreated with rosuvastatin lead to a further increase in expression of P4Hα1 (Fig 2C and 2D). The effects of CD40L or/and rosuvastatin on P4Hα1 expression are consistent with the results shown in Fig 1. However, the expression of P4Hα1 was not influenced by NF-κB siRNAs transfection alone. To measure the transfection efficiency, the expression levels of NF-κB protein in the transfected group compared with the control group were used (Fig 2E).

In addition, the NF-κB nuclear import rate was significantly lower in CD40L stimulated HASMCs pretreated with rosuvastatin than in the untreated CD40L-stimulated HASMCs (Fig 2F). Rosuvastatin itself had no significant effect on the nuclear import rate of NF-κB (S13).

### 3. JNK regulates the effect of rosuvastatin’s rescue of the CD40L stimulated P4Hα1 inhibition in CD40L-stimulated HASMCs

#### 3.1. CD40L induces the phosphorylation of JNK upstream of NF-κB

**Fig 3:**
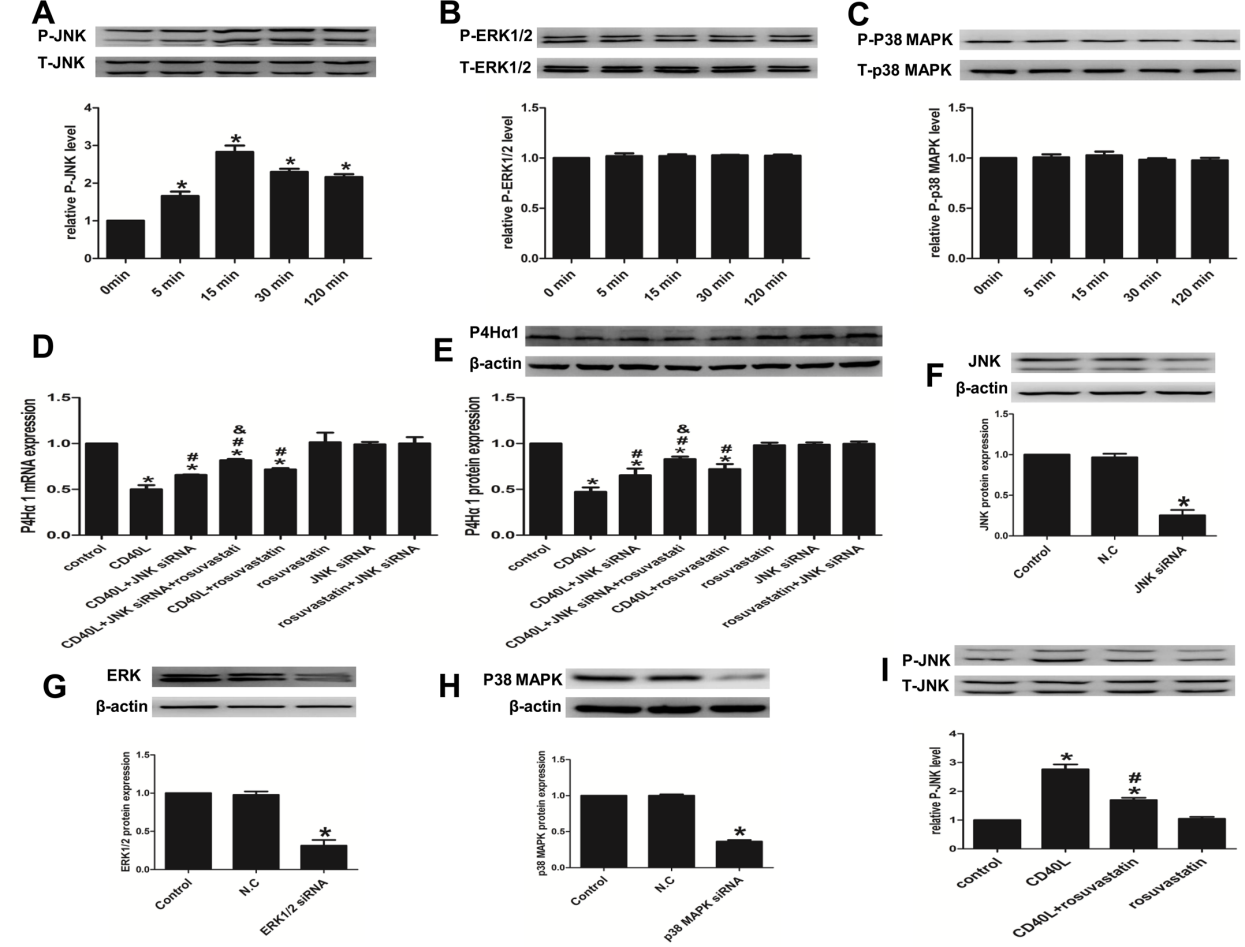
JNK regulates the effect of rosuvastatin’s rescue of the CD40L stimulated P4Hα1 inhibition in CD40L-stimulated HASMCs. (A, B and C) The phosphorylation levels of JNK (A), ERK (B), and p38 MAPK (C) in HASMCs treated with 5μg/ml rhsCD40L for 0, 5, 15, 30 and 120 min western blot analysis. (D and E) P4Hα1 expression in HASMCs treated with or without rhsCD40L, JNK siRNA and rousuvastatin by qRT-PCR (D) and western blot analysis (E). (F, G and H) JNK (F), ERK (G) and p38 MAPK (H) protein expression in HASMCs transfected with or without target siRNA and negative control siRNA. (I) The phosphorylation levels of JNK in HASMCs treated with or without rhsCD40L and rosuvastatin. *P < 0.05 vs. control, ^#^*P* <0.05 vs. CD40L, ^&^*P*<0.05 vs. CD40L+siRNA. N.C: the group transfected with negative control siRNA.

Western blot analysis was performed to elucidate the effect of CD40L on the phosphorylation status of several proteins. The results demonstrated that CD40L significantly induced the phosphorylation of JNK in HASMCs compared with the control cells, with the phosphorylation peaking at 15 min and then decreasing gradually (Fig 3A). Interestingly, CD40L had no effect on the levels of phosphorylated ERK1/2 and p38 MAPK (Fig 3B and 3C).

The NF-κB nuclear import rate was significantly lower in JNK siRNAs transfected CD40L-stimulated HASMCs compared with non-transfected CD40L-stimulated HASMCs (Fig 2F). The nuclear import rates were not influenced by ERK1/2 and p38MAPK siRNAs transfections (S13). Also, no significant changes were observed in the NF-κB nuclear import rate in HASMCs transfected with JNK, ERK1/2 or p38MAPK siRNAs alone (S13).

#### 3.2. Rosuvastatin attenuates the suppressive effect of CD40L on P4Hα1 expression via the JNK pathway

Based on the results obtained above, we further questioned whether treatment with rosuvastatin affected P4Hα1 expression via the JNK pathway. JNK siRNA transfected HASMCs were treated as described previously. The results demonstrated that the P4Hα1 mRNA (Fig 3D) and protein expression (Fig 3E) were significantly higher in the transfected cells stimulated with rhsCD40L compared with non-transfected CD40L stimulated HASMCs. The expression of P4Hα1 increased further when the transfected HASMCs were pretreated with rosuvastatin and then stimulated with CD40L. The effects of CD40L and/or rosuvastatin on P4Hα1 expression are consistent with the results shown in Fig 1. P4Hα1 expression was not influenced by JNK siRNAs transfection alone (Fig 3D and 3E). The expression levels of JNK, ERK1/2 and p38 MAPK protein in the transfected group compared with the control group were used to measure the transfection efficiencies (Fig 3F–3H).

Western blot analysis to quantify the levels of phosphorylated JNK protein revealed that the levels were significantly lower in HASMCs pretreated with rosuvastatin prior to stimulation with the CD40L compared with untreated CD40L stimulated HASMCs. However, rosuvastatin itself had no significant effect on the phosphorylation levels of JNK (Fig 3I).

### 4. TRAF6 regulates the effect of rosuvastatin’s rescue of the CD40L stimulated P4Hα1 inhibition in CD40L-stimulated HASMCs

#### 4.1. CD40L activates TRAF6 upstream of JNK and/or NF-κB

**Fig 4:**
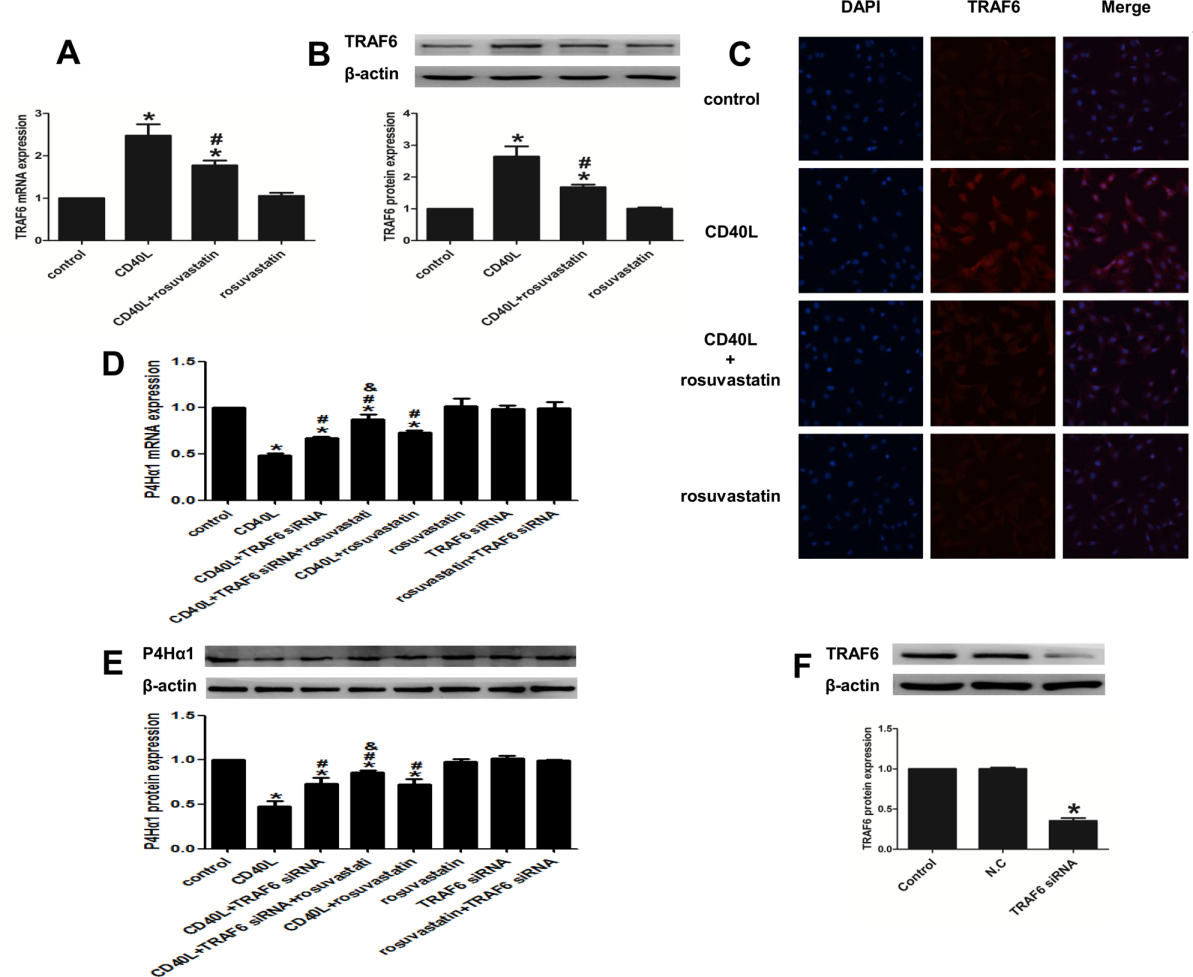
TRAF6 regulates the effect of rosuvastatin’s rescue of the CD40L stimulated P4Hα1 inhibition in CD40L-stimulated HASMCs. (A, B and C) TRAF6 expression in HASMCs treated with or without rhsCD40L and rousuvastatin by qRT-PCR (A), western blot analysis (B) and immunofluorescence (C). (D and E) P4Hα1 expression in HASMCs treated with or without rhsCD40L, TRAF6 siRNA and rousuvastatin by qRT-PCR (D) and western blot analysis (E). (F) TRAF6 protein expression in HASMCs transfected with or without TRAF6 siRNA and negative control siRNA. *P < 0.05 vs. control, #P <0.05 vs. CD40L, &P<0.05 vs. CD40L+siRNA. N.C: the group transfected with negative control siRNA.

In order to determine the downstream signaling effects of TRAF6, HASMCs were stimulated with CD40L for 5 min, and the levels of TRAF6 mRNA and protein were detected by qRT-PCR, Western blot and immunofluorescence. The data indicated that CD40L stimulated HASMCs had significantly higher levels of TRAF6 mRNA (Fig 4A) and protein (Fig 4B and 4C) compared with the unstimulated control cells. In addition, we questioned whether TRAF6 regulated the NF-κB nuclear import rate in CD40-stimulated HASMCs. We therefore transfected the HASMCs with 100 nM TRAF6 siRNAs for 48 h prior to stimulation with 5 μg/ml rhsCD40L for 8 h. The analysis revealed that the transfected cells had significantly lower NF-κB nuclear import rate compared with non-transfected HASMCs, both stimulated with CD40L (Fig 2F). No significant changes were observed in the NF-κB nuclear import rate in HASMCs transfected with TRAF6 siRNAs alone (S13).

#### 4.2. Rosuvastatin attenuates the suppressive effect of CD40L on P4Hα1 expression via TRAF6

P4Hα1 mRNA (Fig 4D) and protein expression (Fig 4E) was significantly higher in HASMCs transfected with TRAF6 siRNAs compared with the non-transfected HASMCs, both stimulated with the CD40L. The expression of P4Hα1 increased further when the transfected cells were pretreated with rosuvastatin prior to being stimulated with CD40L. The effects of CD40L and/or rosuvastatin on P4Hα1 expression are consistent with results shown in Fig 1. Interestingly, P4Hα1 expression was not influenced by TRAF6 siRNAs transfection alone (Fig 4D and 4E). To measure the transfection efficiency, the expression levels of TRAF6 protein in the transfected group compared with the control group were used (Fig 4F).

TRAF6 expression was significantly attenuated in HASMCs pretreated with rosuvastatin before being stimulated with CD40L compared with CD40L stimulated cells alone. Rosuvastatin itself had no significant effect on the expression of TRAF6 (Fig 4A–4C).

## Discussion

In the current study, we found that (1) HASMCs stimulation of with the CD40L inhibits the expression of P4Hα1 via activation of the TRAF6-JNK-NF-κB pathways, (2) Cultured HASMCs take no role in the expression changes of MMP2 and MMP9 under a P4Hα1-affected CD40L stimulation range, and (3) treatment with rosuvastatin attenuated the CD40L-mediated suppressive effect on the expression of P4Hα1 via inhibition of the CD40L-activated TRAF6-JNK- NF-κB pathways. This study therefore, contributes towards understanding the direct effects of CD40L stimulation and/or rosuvastatin on P4Hα1 expression in HASMCs, and the underlying signaling mechanisms, which may provide evidence for collagen homeostasis in atherosclerotic plaque ECM.

The collagen-rich fibrous cap that covers the lipid core, and its thickness are crucial to the stability of an atherosclerotic plaque, whereas P4Hα1 and MMPs play important roles in the production and degradation of a fibrous cap, respectively [1–7]. Atherosclerosis is believed to be a chronic inflammatory-immune disease, as a variety of cytokines participate in the pathogenesis of atherosclerosis. Special emphasis is placed on the role of pro- and anti-inflammatory cytokines in pathogenic and regulatory immunity [36]. Several *in vitro* studies have shown that TNF-α, IL-6 and TGF-β1 affect P4Hα1 expression in the ECM of an atherosclerotic plaque [37–39]. CD40L is a protein that is involved in the pathogenesis of atherosclerosis, and is known to actively regulate ECM metabolism [12–16]. Previous studies have mainly focused on CD40L-mediated ECM degradation via upregulated MMPs [17–19]. In the present study, we found that CD40L dose- and time-dependently reduced P4Hα1 mRNA and protein expression as well as type 1 and 3 collagen production in HASMCs. The data indicate that CD40L inhibits collagen synthesis via suppression of P4Hα1, which simultaneously increases ECM degradation and decreases its production, thereby contributing to atherosclerotic plaque rupture. Moreover, CD40L stimulation of the HASMCs did not significantly alter the expression of MMP2 and the negative enzymatic activity of MMP9, suggesting that CD40L exerts its affect independent of these matrix degrading enzymes in HASMs, at least within the range that reduced P4Hα1 expression.

Statins are cholesterol lowering drugs that have been widely prescribed in cardiovascular diseases however, over the years, several studies have reported about its pleiotropic, cholesterol-independent effects. [27, 28] Additionally, it has shown that statins exhibit the cholesterol-independent effect by decreasing the inflammation associated with atherosclerosis [31, 32], and also by stabilizing the plaques [33, 34]. Rosuvastatin, a member of new generation of statins, has also been shown to display similar effects [40, 41]. Interestingly, as with CD40L, most of the previous studies on statins mainly focused on the inhibition of matrix degradation, and therefore, the effects on matrix production remained poorly understood. To the best of our knowledge, the current study is the first to demonstrate that rosuvastatin attenuates CD40L-induced suppressive effect on P4Hα1 mRNA and protein expression, as well as the resulting collagen protein expression. However, rosuvastatin treatment without CD40L stimulation had no effect on the expression of P4Hα1 and collagen in HASMCs. These results are consistent with a previous study showing that P4Hα1 and collagen expression did not change with statin treatment alone in cultured HASMCs [42].

The CD40 signal transduction pathway differs significantly with cell type, function, origin, stage of differentiation and activation status [11, 12, 23, 24]. The canonical TRAFs-MAPKs-NF-κB pathway mediates various proatherogenic processes in atheroma-associated cell types [11, 12, 23]. NF-κB controls the expression of genes directing the initiation, progression and resolution of atherosclerotic plaque, including genes for proatherogenic cytokines such as TNF-α, IL-1β, IL-6, chemokine MCP-1, adhesion molecule ICAM-1 and MMPs, where few of these factors persistently maintain NF-κB in an activated state [26, 43–46]. MAPKs play important roles in the pathogenesis of atherosclerosis, proinflammation and collagen turnover. ^25^ Previous studies have shown that JNK and ERK1/2 mediate the suppressive effects of TNFα and IL-6 on P4Hα1 expression in HASMCs [38, 39]. TNFα shares structural homologies with CD40L, and they both belong to the TNF superfamily. Furthermore, Zhang K et al. demonstrated that oxidized-low density lipoprotein can inhibit the expression of P4Hα1 in HASMCs however, treatment with statins can abrogate this effect leading to increased P4Hα1 via p38 MAPK and ERK1/2 signaling pathway [42]. TRAF6 is a relatively independent CD40-binding protein compared with TRAF1/2/3/4/5, and CD40-TRAF6 interactions are capable of activating the canonical NF-κB pathway [23]. In the present study, we found that CD40L upregulated TRAF6 expression, JNK phosphorylation, and NF-κB nuclear translocation, as well as DNA binding, suggesting that the suppressive effect of CD40L on P4Hα1 and collagen expression in HASMCs is mediated by TRAF6, JNK and NF-κB. These finding were further confirmed by transfection studies, where the suppressive effect of CD40L on P4Hα1 expression was abolished by silencing TRAF6, JNK and NF-κB genes. Several studies have shown NF-κB to be a transcription factor for CD40, and therefore, the NF-κB nuclear import rate was measured to confirm the involvement of this pathway [24]. We found that JNK or TRAF6 siRNA transfected HASMCs when stimulated with CD40L displayed lower NF-κB nuclear import rate compared with non-transfected CD40L-stimulated HASMCs. Most importantly, we found that rosuvastatin treatment attenuated the suppressive effect of CD40L on P4Hα1 and collagen expression via inhibition of CD40L activated TRAF6-JNK-NF-κB pathways.

## Conclusions

CD40L suppressed P4Hα1 and collagen expression via activation of TRAF6-JNK- NF-κB signaling pathways, and this effect was significantly reversed by rosuvastatin. This suggests that CD40L and statins regulate atherosclerotic plaque ECM production in HASMCs. These findings provide a novel mechanism for stabilizing vulnerable atherosclerotic plaques.

## Supporting Information

S1 Fig First β-Actin original Western blot image for Fig 1B. (TIF)

S2 Fig First collagen1 original Western blot image for Fig 1B. (TIF)

S3 Fig First collagen3 original Western blot image for Fig 1B. (TIF)

S4 Fig First P4Hα1 original Western blot image for Fig 1B. (TIF)

S5 Fig Second β-Actin (collagen1) original Western blot image for Fig 1B. (TIF)

S6 Fig Second β-Actin (collagen3) original Western blot image for Fig 1B. (TIF)

S7 Fig Second β-Actin (P4Hα1) original Western blot image for Fig 1B. (TIF)

S8 Fig Second collagen1 original Western blot image for Fig 1B. (TIF)

S9 Fig Second collagen3 original Western blot image for Fig 1B. (TIF)

S10 Fig Second P4Hα1 original Western blot image for Fig 1B. (TIF)

S11 Fig Third β-Actin (collagen1) original Western blot image for Fig 1B. (TIF)

S12 Fig Third β-Actin (collagen1) original Western blot image for Fig 1B. (TIF)

S13 Fig Third β-Actin (P4Hα1) original Western blot image for Fig 1B. (TIF)

S14 Fig Third collagen1 original Western blot image for Fig 1B. (TIF)

S15 Fig Third collagen3 original Western blot image for Fig 1B. (TIF)

S16 Fig Third P4Hα1 original Western blot image for Fig 1B. (TIF)

S17 Fig First β-Actin original Western blot image for Fig 1D. (TIF)

S18 Fig First P4Hα1 original Western blot image for Fig 1D. (TIF)

S19 Fig Second β-Actin original Western blot image for Fig 1D. (TIF)

S20 Fig Second P4Hα1 original Western blot image for Fig 1D. (TIF)

S21 Fig Third β-Actin original Western blot image for Fig 1D. (TIF)

S22 Fig Third P4Hα1 original Western blot image for Fig 1D. (TIF)

S23 Fig First β-Actin original Western blot image for Fig 1H. (TIF)

S24 Fig First collagen1 original Western blot image for Fig 1H. (TIF)

S25 Fig First collagen3 original Western blot image for Fig 1H. (TIF)

S26 Fig First P4Hα1 original Western blot image for Fig 1H. (TIF)

S27 Fig Second β-Actin original Western blot image for Fig 1H. (TIF)

S28 Fig Second collagen1 original Western blot image for Fig 1H. (TIF)

S29 Fig Second collagen3 original Western blot image for Fig 1H. (TIF)

S30 Fig Second P4Hα1 original Western blot image for Fig 1H. (TIF)

S31 Fig Third β-Actin original Western blot image for Fig 1H. (TIF)

S32 Fig Third collagen1 original Western blot image for Fig 1H. (TIF)

S33 Fig Third collagen3 original Western blot image for Fig 1H. (TIF)

S34 Fig Third P4Hα1 original Western blot image for Fig 1H. (TIF)

S35 Fig First β-Actin original Western blot image for Fig 2D. (TIF)

S36 Fig First P4Hα1 original Western blot image for Fig 2D. (TIF)

S37 Fig Second β-Actin original Western blot image for Figs 2D, 3E and 4E. (TIF)

S38 Fig Second P4Hα1 original Western blot image for Figs 2D, 3E and 4E. (TIF)

S39 Fig Third β-Actin original Western blot image for Figs 2D, 3E and 4E. (TIF)

S40 Fig Third P4Hα1 original Western blot image for Figs 2D, 3E and 4E. (TIF)

S41 Fig First β-Actin original Western blot image for Fig 2E. (TIF)

S42 Fig First NF-κB original Western blot image for Fig 2E. (TIF)

S43 Fig Second β-Actin original Western blot image for Fig 2E. (TIF)

S44 Fig Second NF-κB original Western blot image for Fig 2E. (TIF)

S45 Fig Third β-Actin original Western blot image for Fig 2E. (TIF)

S46 Fig Third NF-κB original Western blot image for Fig 2E. (TIF)

S47 Fig First phospho-JNK original Western blot image for Fig 3A. (TIF)

S48 Fig First JNK original Western blot image for Fig 3A. (TIF)

S49 Fig Second phospho-JNK original Western blot image for Fig 3A. (TIF)

S50 Fig Second JNK original Western blot image for Fig 3A. (TIF)

S51 Fig Third phospho-JNK original Western blot image for Fig 3A. (TIF)

S52 Fig Third JNK original Western blot image for Fig 3A. (TIF)

S53 Fig Fourth phospho-JNK original Western blot image for Fig 3A. (TIF)

S54 Fig Fourth JNK original Western blot image for Fig 3A. (TIF)

S55 Fig First phospho-ERK1/2 original Western blot image for Fig 3B. (TIF)

S56 Fig First ERK1/2 original Western blot image for Fig 3B. (TIF)

S57 Fig Second phospho-ERK1/2 original Western blot image for Fig 3B. (TIF)

S58 Fig Second ERK1/2 original Western blot image for Fig 3B. (TIF)

S59 Fig Third phospho-ERK1/2 original Western blot image for Fig 3B. (TIF)

S60 Fig Third ERK1/2 original Western blot image for Fig 3B. (TIF)

S61 Fig First phospho-p38MAPK original Western blot image for Fig 3C. (TIF)

S62 Fig First p38MAPK original Western blot image for Fig 3C. (TIF)

S63 Fig Second phospho-p38MAPK original Western blot image for Fig 3C. (TIF)

S64 Fig Second p38MAPK original Western blot image for Fig 3C. (TIF)

S65 Fig Third phospho-p38MAPK original Western blot image for Fig 3C. (TIF)

S66 Fig Third p38MAPK original Western blot image for Fig 3C. (TIF)

S67 Fig First β-Actin original Western blot image for Fig 3E. (TIF)

S68 Fig First P4Hα1 original Western blot image for Fig 3E. (TIF)

S69 Fig First β-Actin original Western blot image for Fig 3F. (TIF)

S70 Fig First JNK original Western blot image for Fig 3F. (TIF)

S71 Fig Second β-Actin original Western blot image for Fig 3F. (TIF)

S72 Fig Second JNK original Western blot image for Fig 3F. (TIF)

S73 Fig Third β-Actin original Western blot image for Fig 3F. (TIF)

S74 Fig Third JNK original Western blot image for Fig 3F. (TIF)

S75 Fig First β-Actin original Western blot image for Fig 3G. (TIF)

S76 Fig First ERK1/2 original Western blot image for Fig 3G. (TIF)

S77 Fig Second β-Actin original Western blot image for Fig 3G. (TIF)

S78 Fig Second ERK1/2 original Western blot image for Fig 3G. (TIF)

S79 Fig Third β-Actin original Western blot image for Fig 3G. (TIF)

S80 Fig Third ERK1/2 original Western blot image for Fig 3G. (TIF)

S81 Fig First β-Actin original Western blot image for Fig 3H. (TIF)

S82 Fig First p38MAPK original Western blot image for Fig 3H. (TIF)

S83 Fig Second β-Actin original Western blot image for Fig 3H. (TIF)

S84 Fig Second p38MAPK original Western blot image for Fig 3H. (TIF)

S85 Fig Third β-Actin original Western blot image for Fig 3H. (TIF)

S86 Fig Third p38MAPK original Western blot image for Fig 3H. (TIF)

S87 Fig First phospho-JNK original Western blot image for Fig 3I. (TIF)

S88 Fig First JNK original Western blot image for Fig 3I. (TIF)

S89 Fig Second phospho-JNK original Western blot image for Fig 3I. (TIF)

S90 Fig Second JNK original Western blot image for Fig 3I. (TIF)

S91 Fig Third phospho-JNK original Western blot image for Fig 3I. (TIF)

S92 Fig Third JNK original Western blot image for Fig 3I. (TIF)

S93 Fig Fourth phospho-JNK original Western blot image for Fig 3I. (TIF)

S94 Fig Fourth JNK original Western blot image for Fig 3I. (TIF)

S95 Fig First β-Actin original Western blot image for Fig 4B. (TIF)

S96 Fig First TRAF6 original Western blot image for Fig 4B. (TIF)

S97 Fig Second β-Actin original Western blot image for Fig 4B. (TIF)

S98 Fig Second TRAF6 original Western blot image for Fig 4B. (TIF)

S99 Fig Third β-Actin original Western blot image for Fig 4B. (TIF)

S100 Fig Third TRAF6 original Western blot image for Fig 4B. (TIF)

S101 Fig First β-Actin original Western blot image for Fig 4E. (TIF)

S102 Fig First P4Hα1 original Western blot image for Fig 4E. (TIF)

S103 Fig First β-Actin original Western blot image for Fig 4F. (TIF)

S104 Fig First TRAF6 original Western blot image for Fig 4F. (TIF)

S105 Fig Second β-Actin original Western blot image for Fig 4F. (TIF)

S106 Fig Second TRAF6 original Western blot image for Fig 4F. (TIF)

S107 Fig Third β-Actin original Western blot image for Fig 4F. (TIF)

S108 Fig Third TRAF6 original Western blot image for Fig 4F. (TIF)

S1 Table CT and RQ value of Fig 1A. (XLSX)

S2 Table Gray value statistic of Fig 1B. (XLSX)

S3 Table CT and RQ value of Fig 1C. (XLSX)

S4 Table Gray value statistic of Fig 1D. (XLSX)

S5 Table Gray value statistic of Fig 1E. (XLSX)

S6 Table Gray value statistic of Fig 1F. (XLSX)

S7 Table CT and RQ value of Fig 1G. (XLSX)

S8 Table Gray value statistic of Fig 1H. (XLSX)

S9 Table Gray value statistic of Fig 2B. (XLSX)

S10 Table CT and RQ value of Fig 2C. (XLSX)

S11 Table Gray value statistic of Fig 2D. (XLSX)

S12 Table Gray value statistic of Fig 2E. (XLSX)

S13 Table NF-κB nuclear import rate statistic. (XLSX)

S14 Table Gray value statistic of Fig 3A. (XLSX)

S15 Table Gray value statistic of Fig 3B. (XLSX)

S16 Table Gray value statistic of Fig 3C. (XLSX)

S17 Table CT and RQ value of Fig 3D. (XLSX)

S18 Table Gray value statistic of Fig 3E. (XLSX)

S19 Table Gray value statistic of Fig 3F. (XLSX)

S20 Table Gray value statistic of Fig 3G. (XLSX)

S21 Table Gray value statistic of Fig 3H. (XLSX)

S22 Table Gray value statistic of Fig 3I. (XLSX)

S23 Table CT and RQ value of Fig 4A. (XLSX)

S24 Table Gray value statistic of Fig 4B. (XLSX)

S25 Table CT and RQ value of Fig 4D. (XLSX)

S26 Table Gray value statistic of Fig 4E. (XLSX)

S27 Table Gray value statistic of Fig 4F. (XLSX)
