# Supplementary figures and images for: Rosuvastatin Attenuates CD40L-Induced Downregulation of Extracellular Matrix Production in Human Aortic Smooth Muscle Cells via TRAF6-JNK-NF-κB Pathway
Source: PLoS One. 2016 Apr 27;11(4):e0153919. doi: 10.1371/journal.pone.0153919 (PMC4847831; doi:10.1371/journal.pone.0153919)

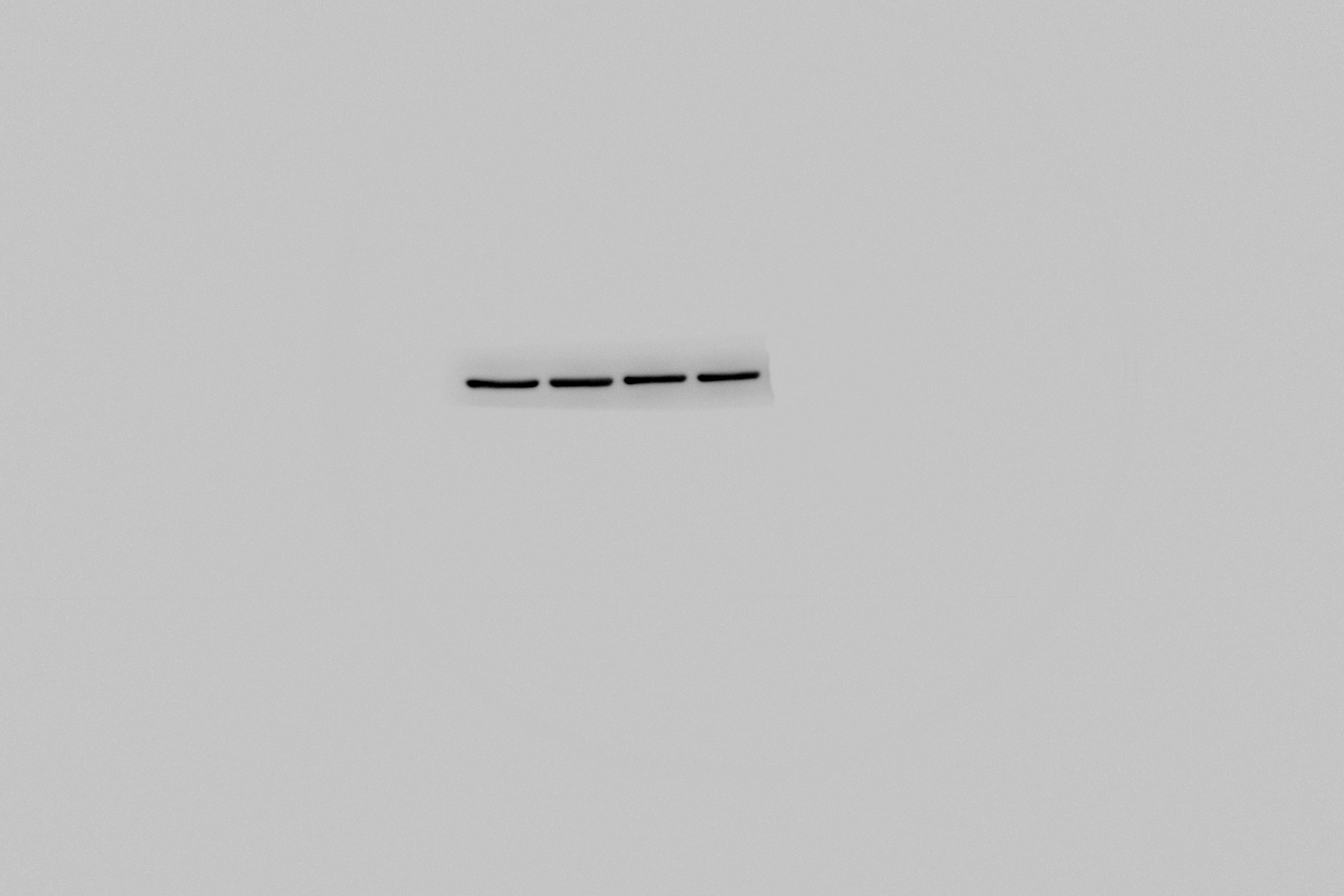

Supplement: S1 Fig — (TIF) [file pone.0153919.s001.tif]

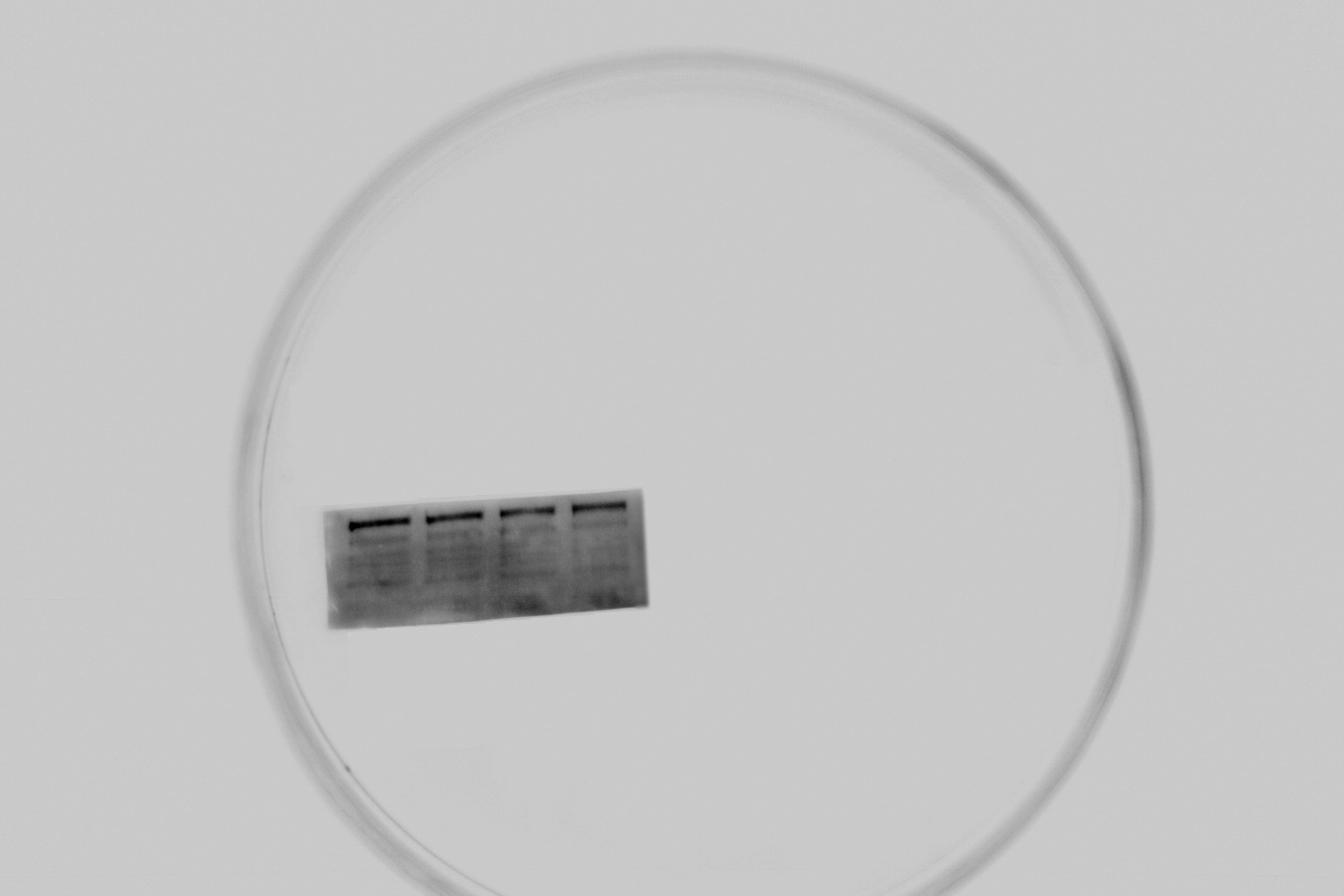

Supplement: S2 Fig — (TIF) [file pone.0153919.s002.tif]

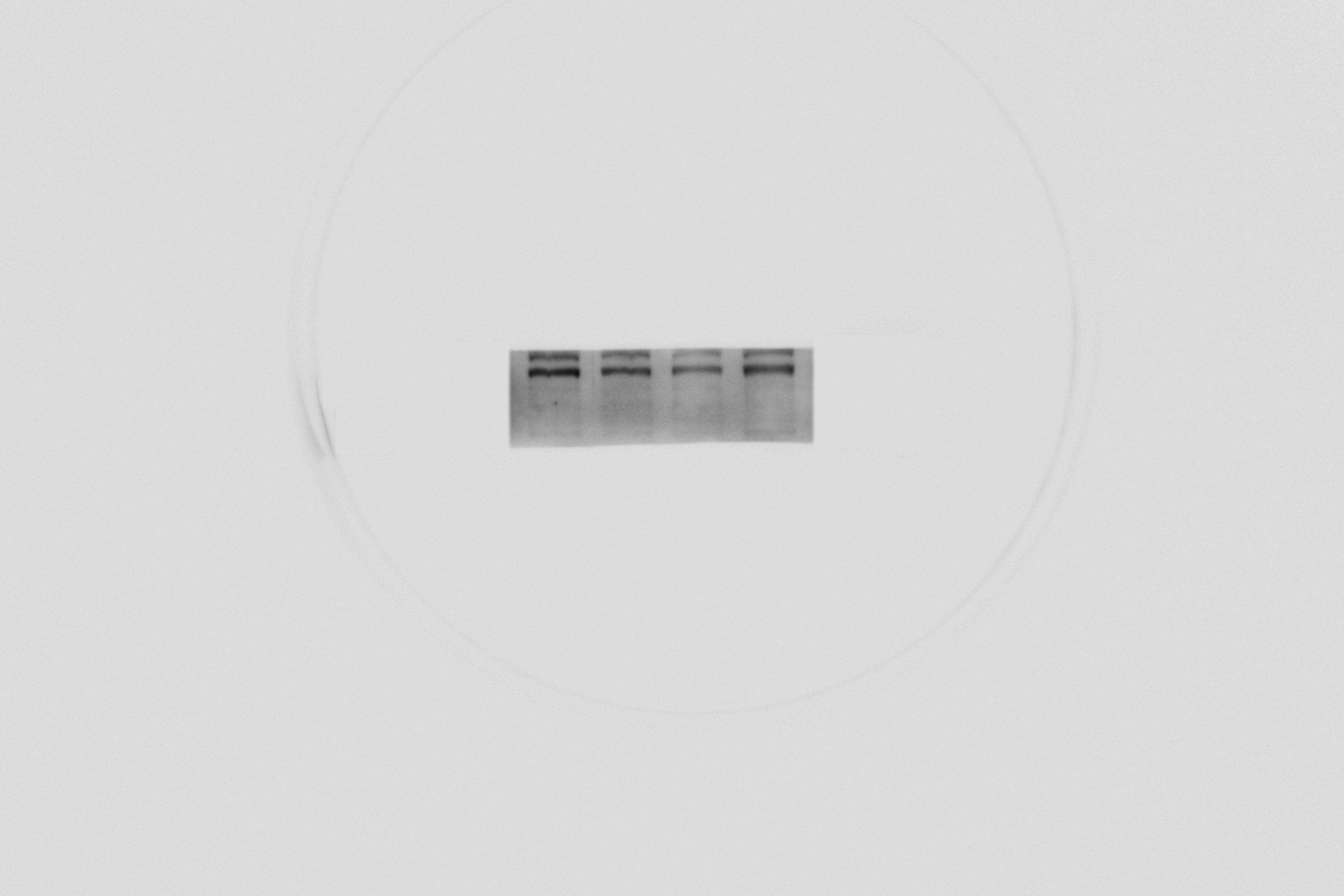

Supplement: S3 Fig — (TIF) [file pone.0153919.s003.tif]

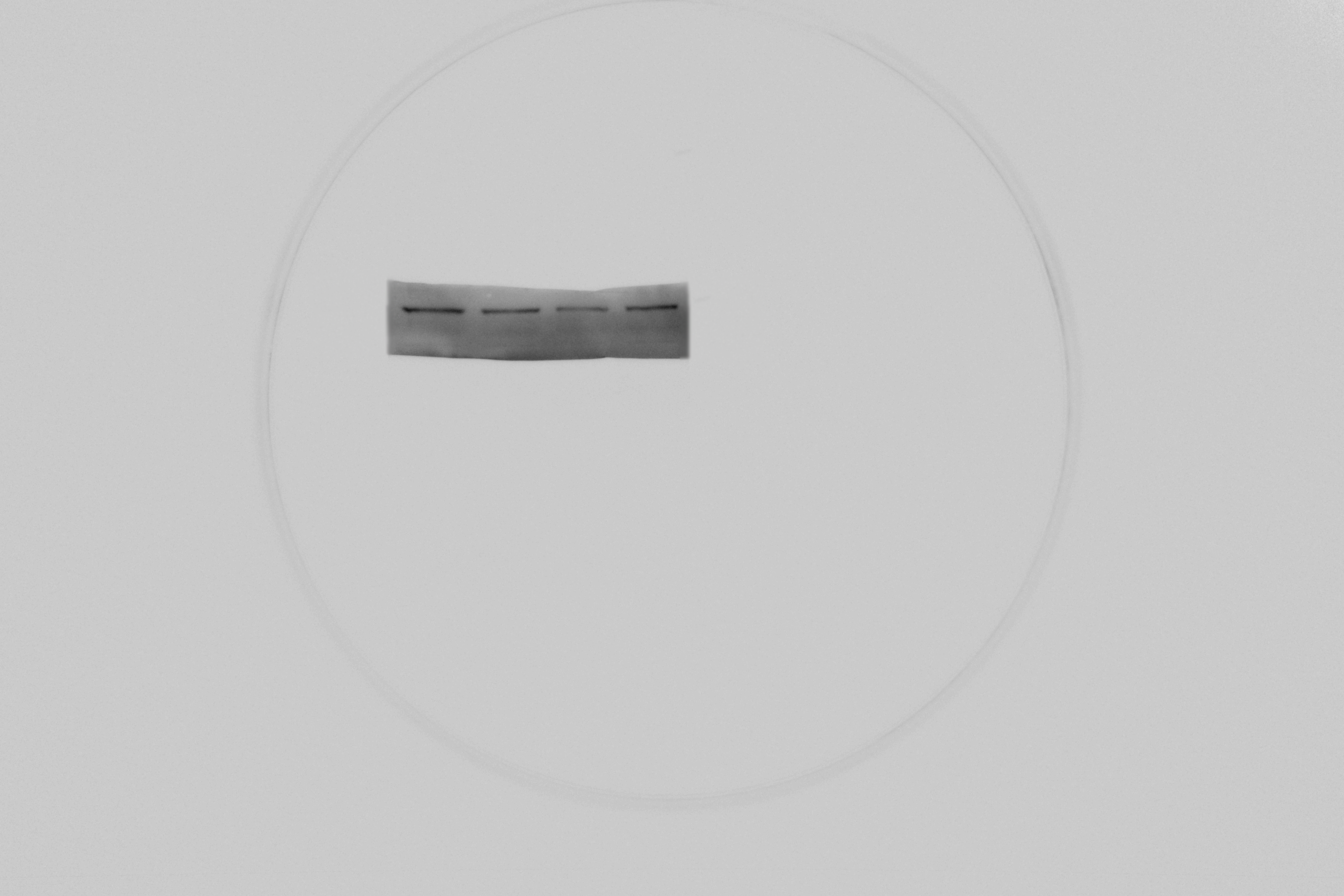

Supplement: S4 Fig — (TIF) [file pone.0153919.s004.tif]

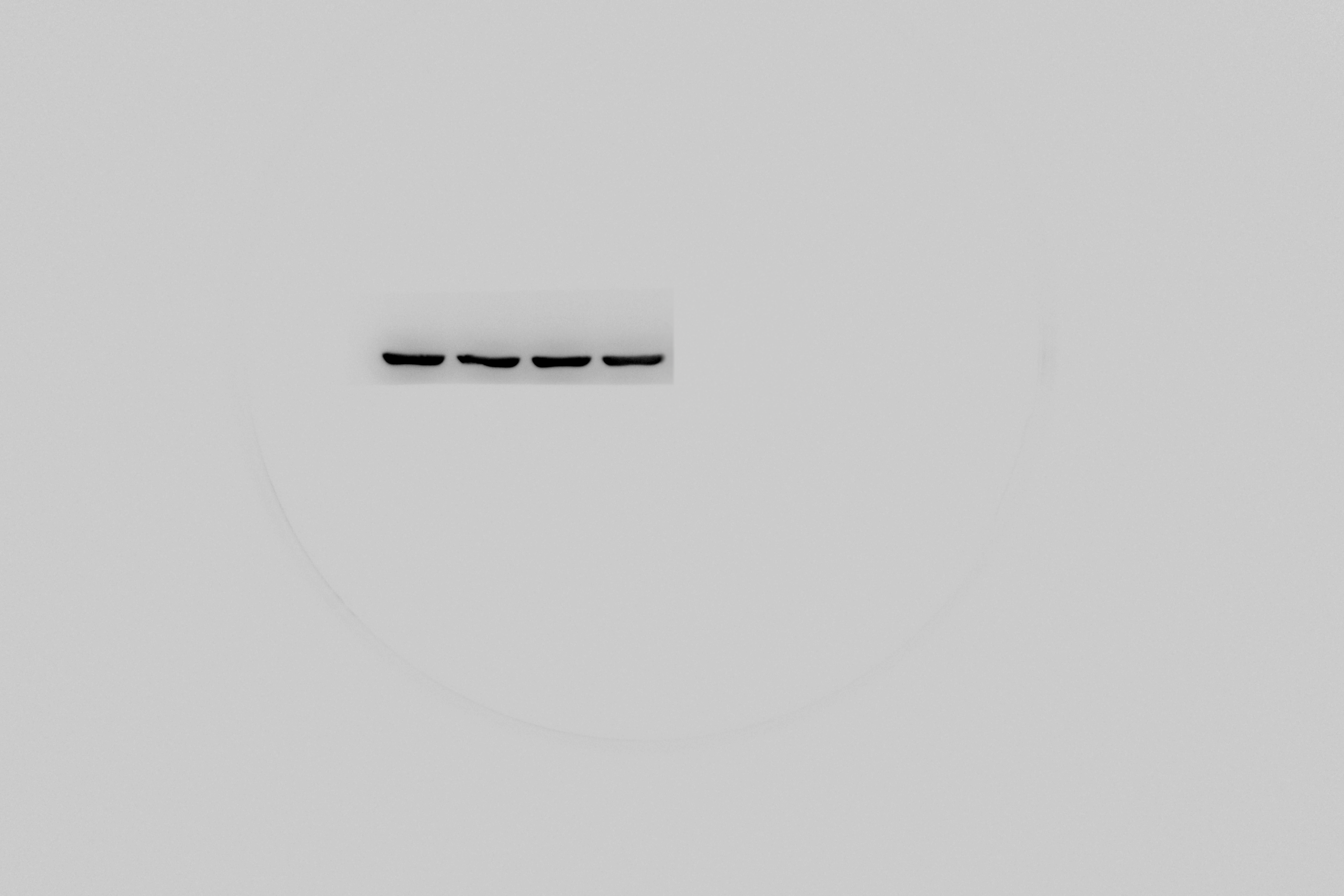

Supplement: S5 Fig — (TIF) [file pone.0153919.s005.tif]

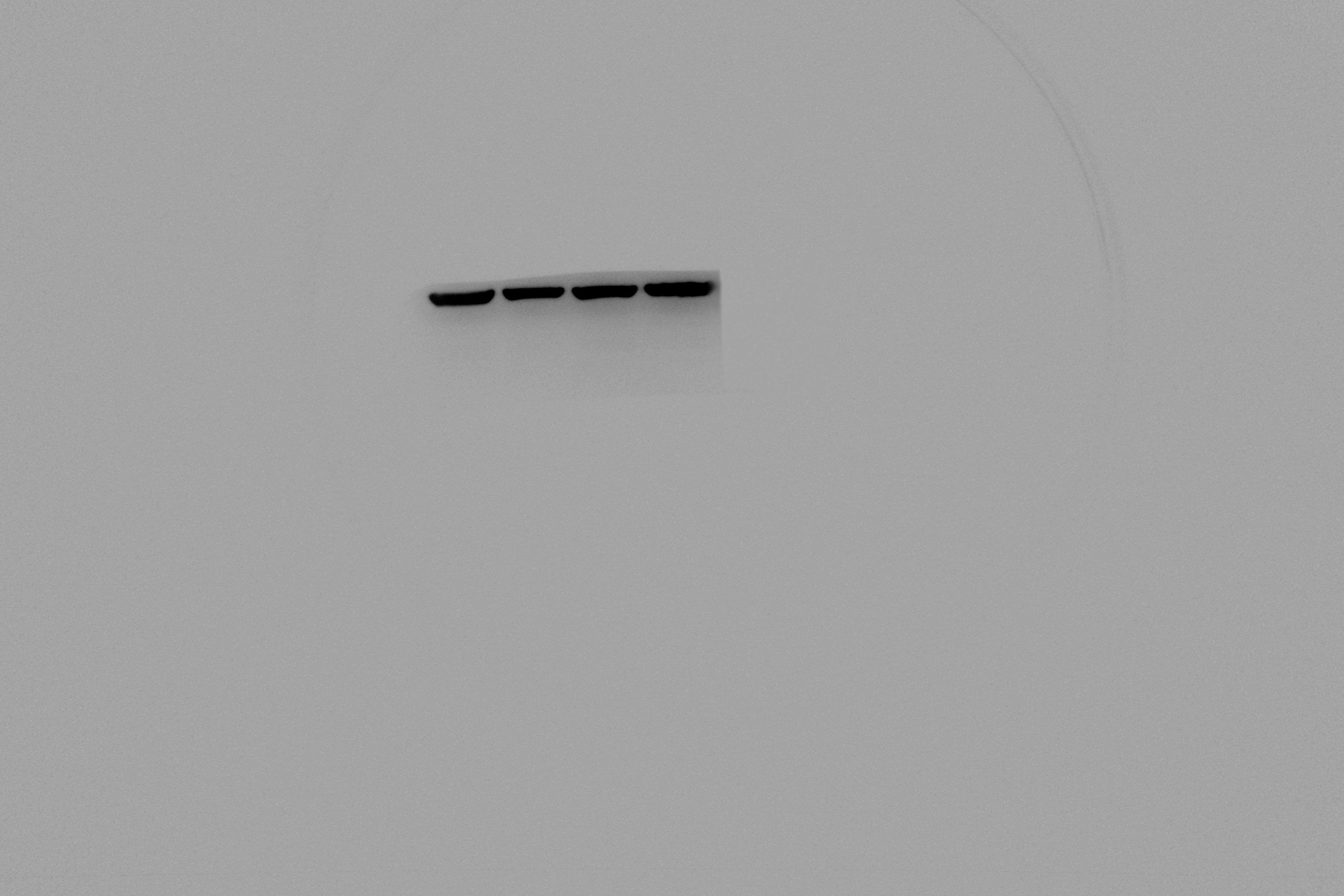

Supplement: S6 Fig — (TIF) [file pone.0153919.s006.tif]

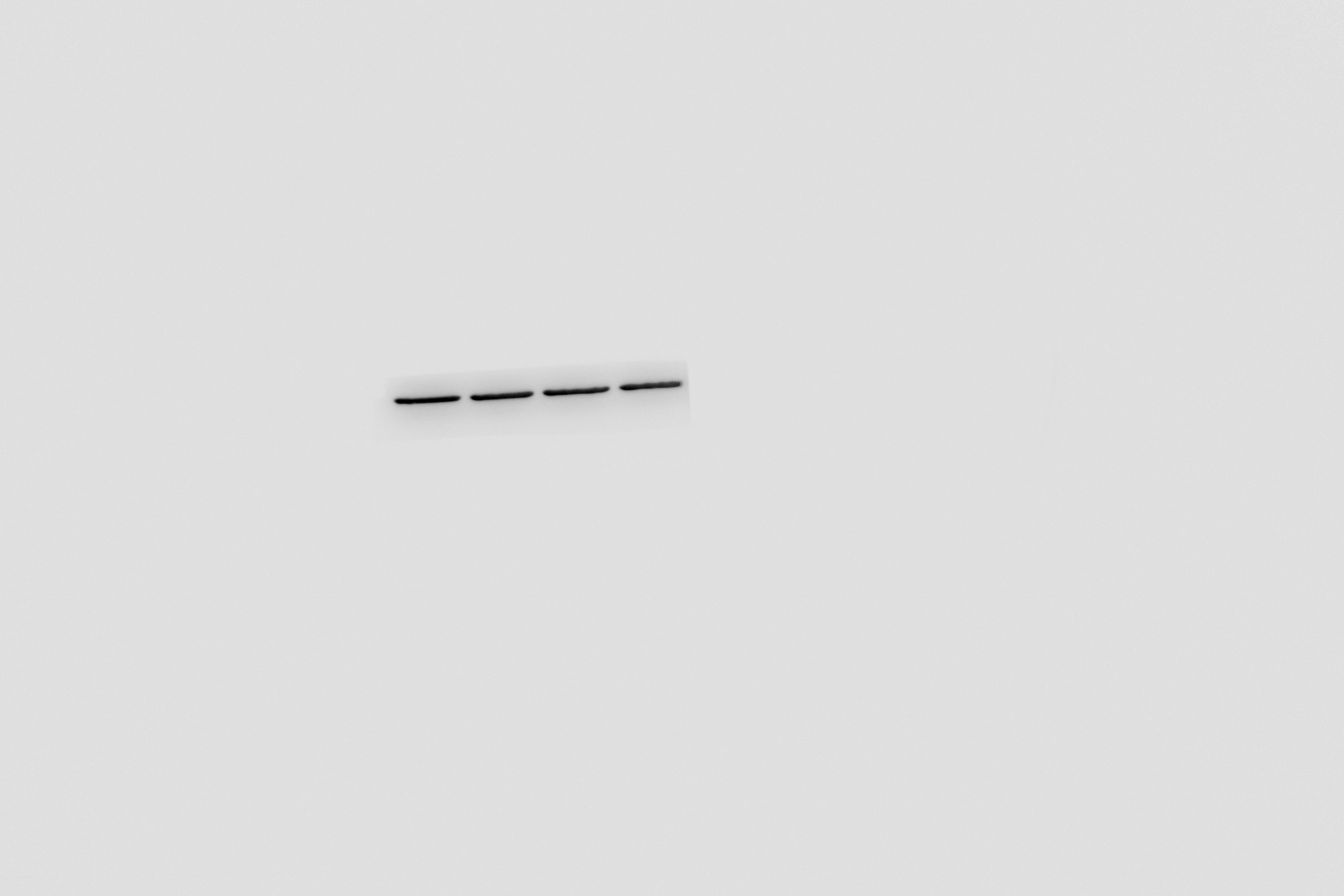

Supplement: S7 Fig — (TIF) [file pone.0153919.s007.tif]

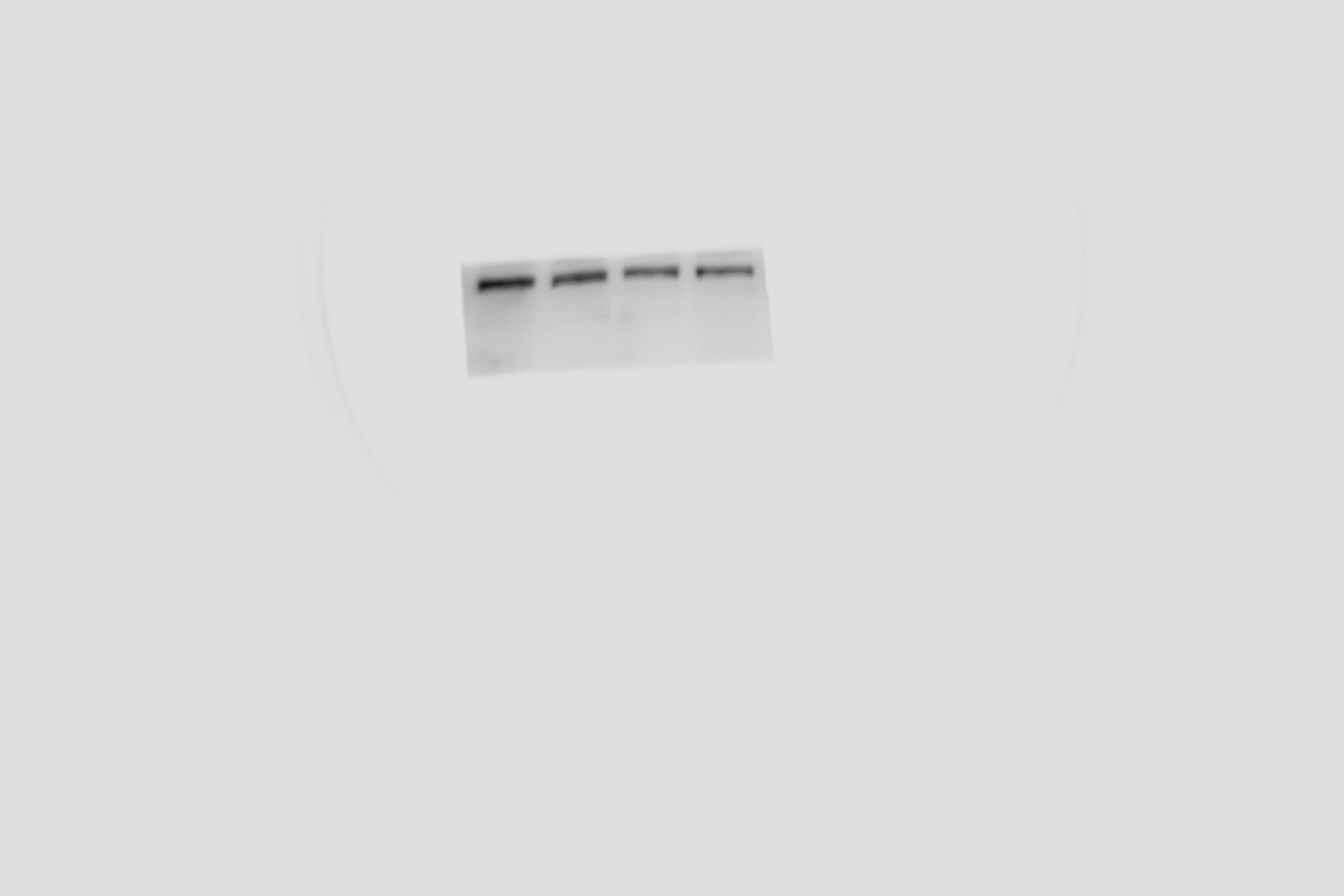

Supplement: S8 Fig — (TIF) [file pone.0153919.s008.tif]

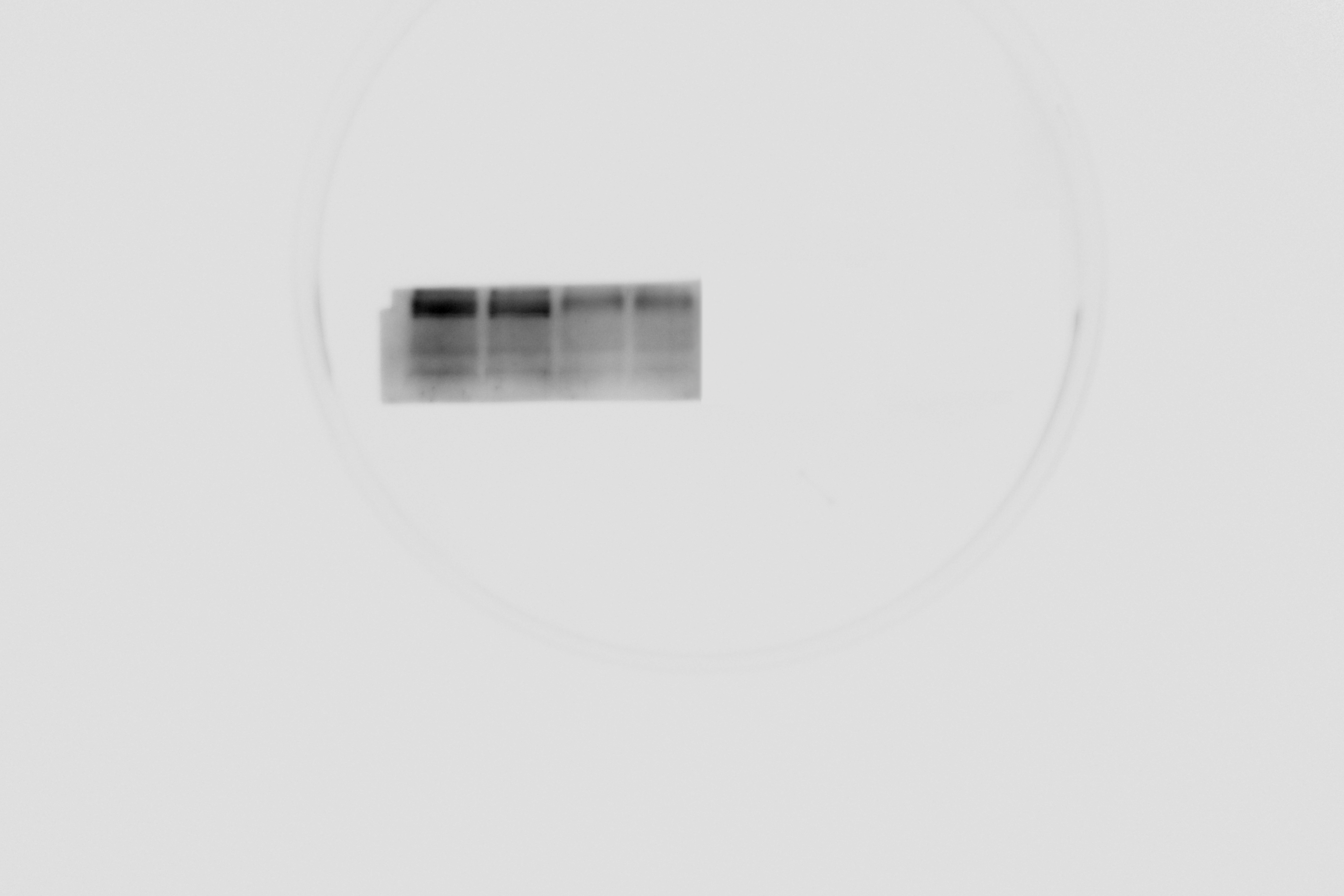

Supplement: S9 Fig — (TIF) [file pone.0153919.s009.tif]

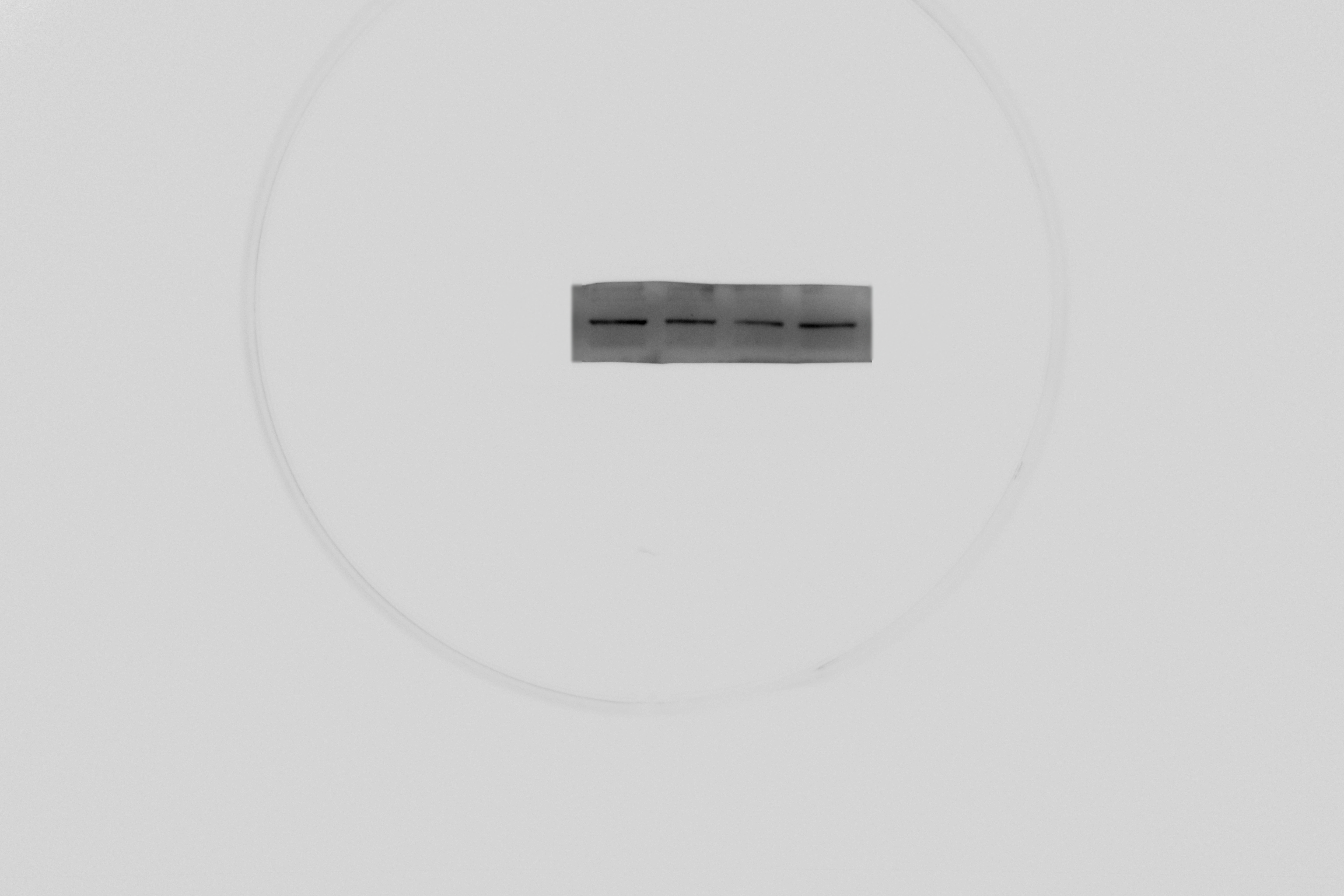

Supplement: S10 Fig — (TIF) [file pone.0153919.s010.tif]

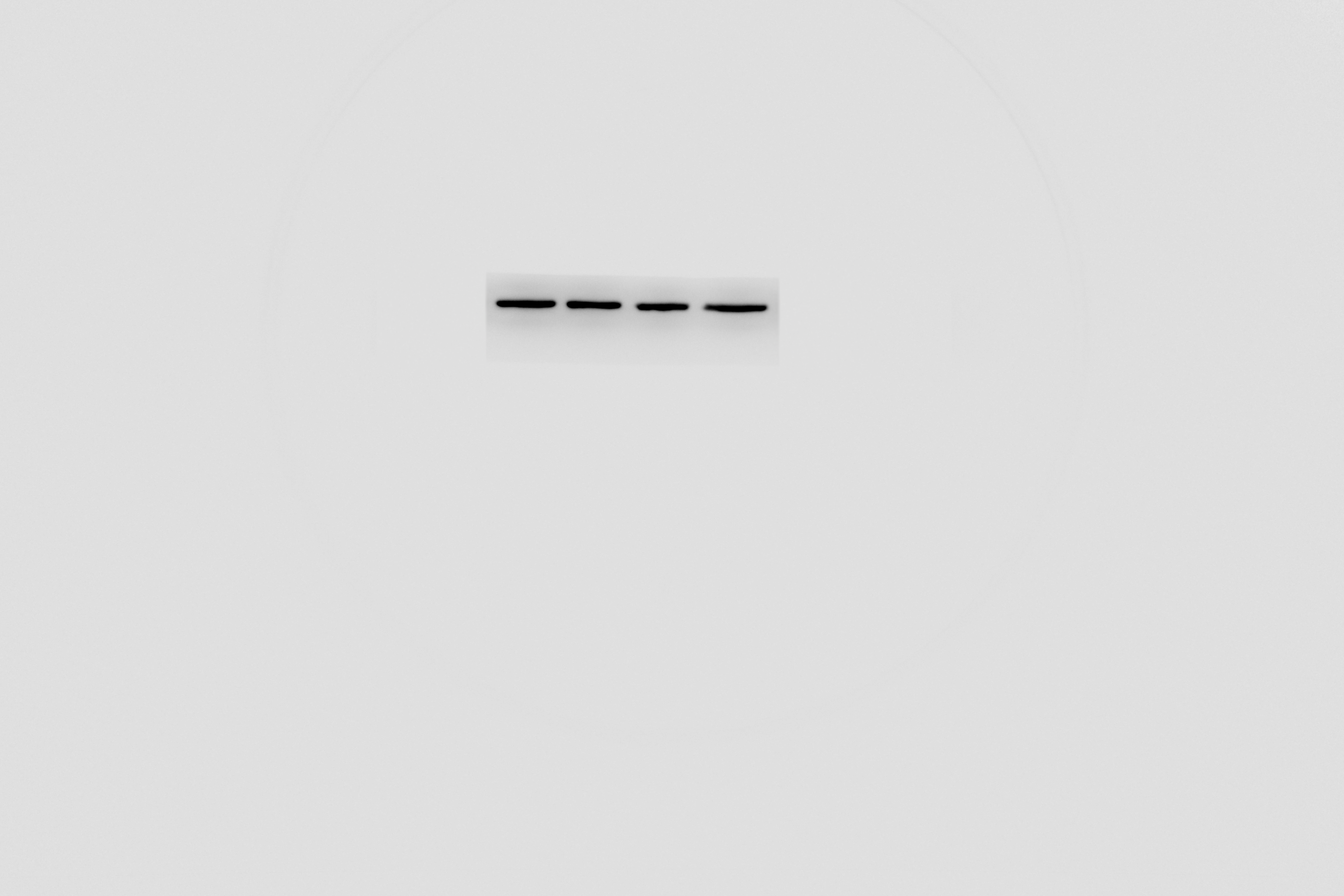

Supplement: S11 Fig — (TIF) [file pone.0153919.s011.tif]

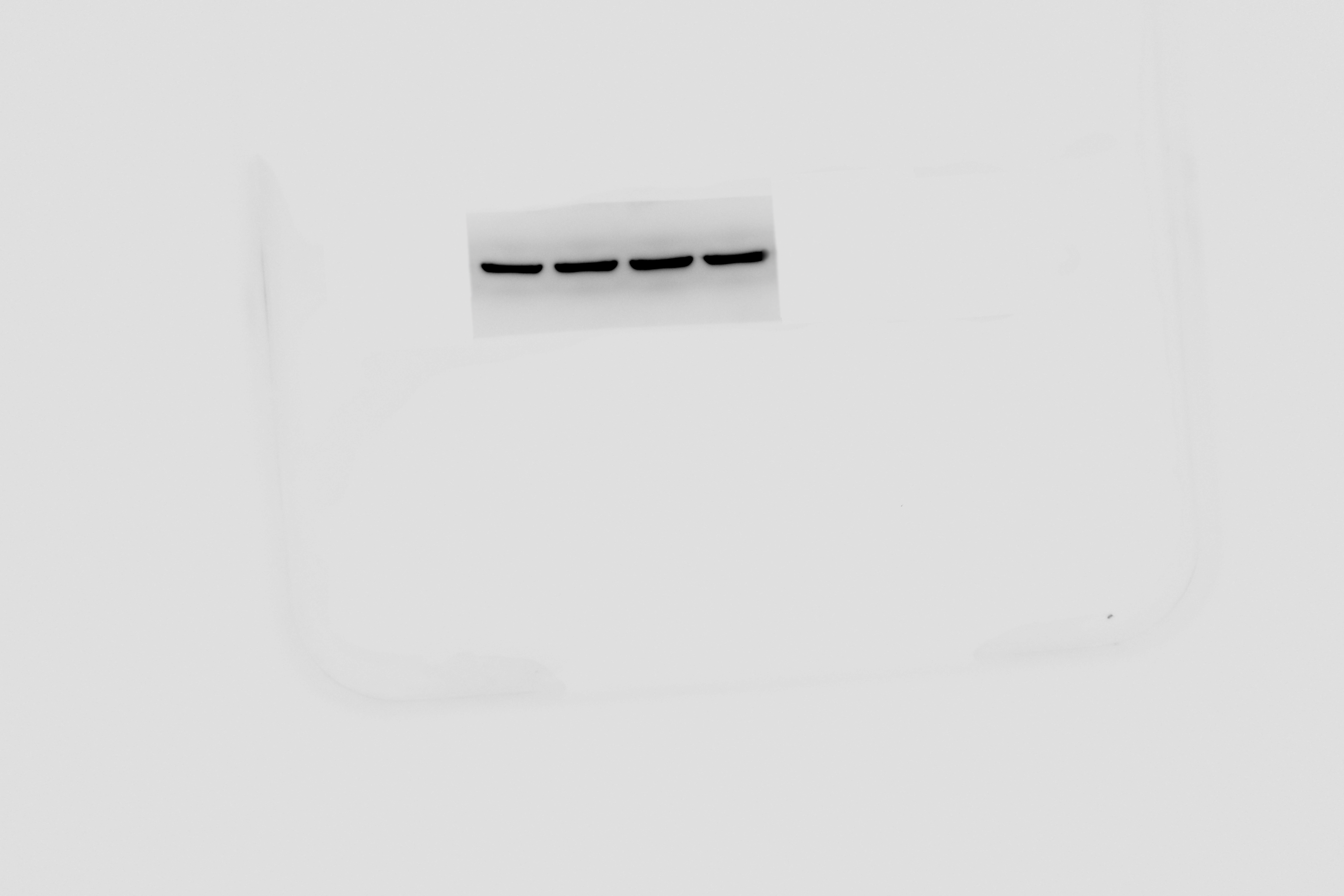

Supplement: S12 Fig — (TIF) [file pone.0153919.s012.tif]

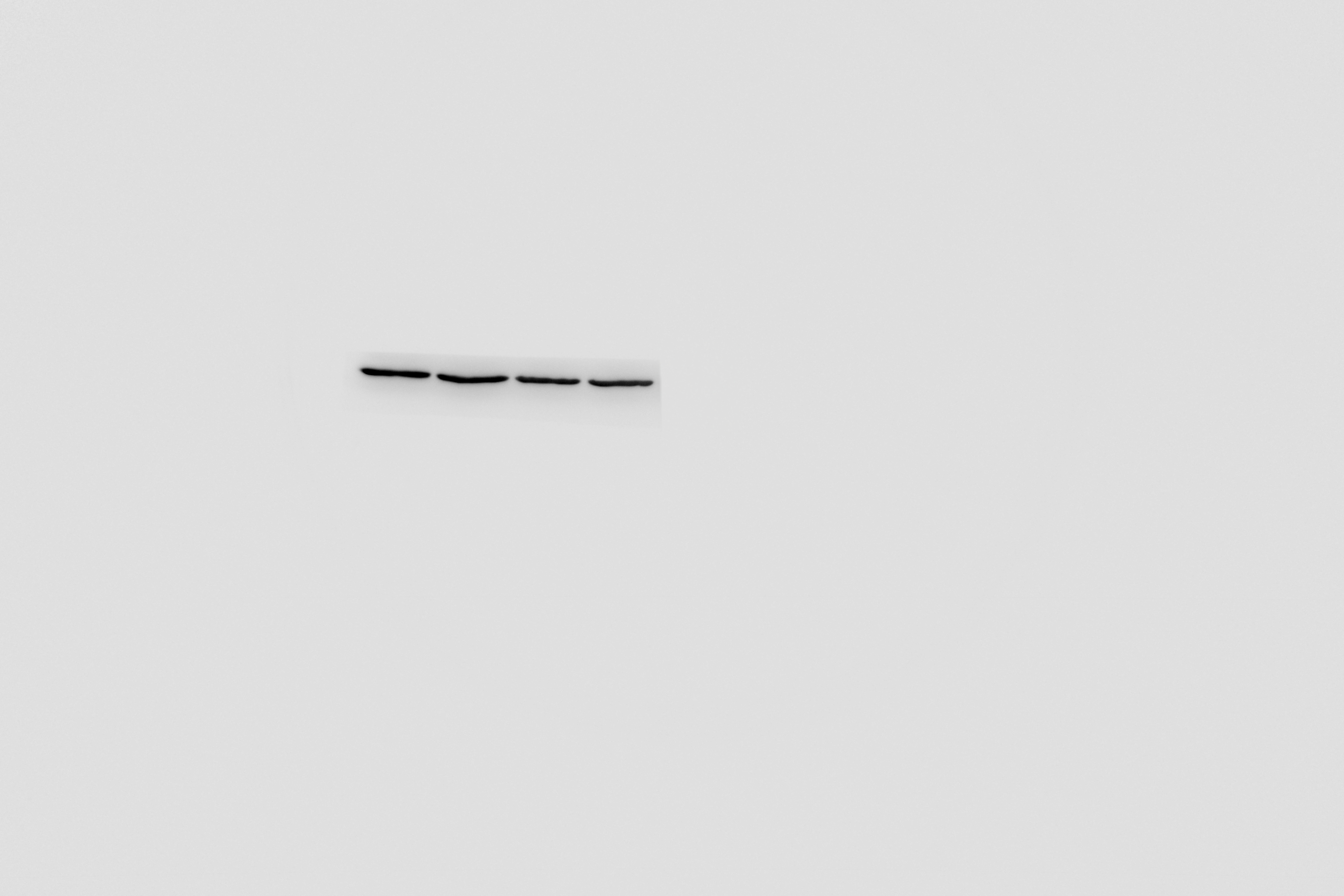

Supplement: S13 Fig — (TIF) [file pone.0153919.s013.tif]

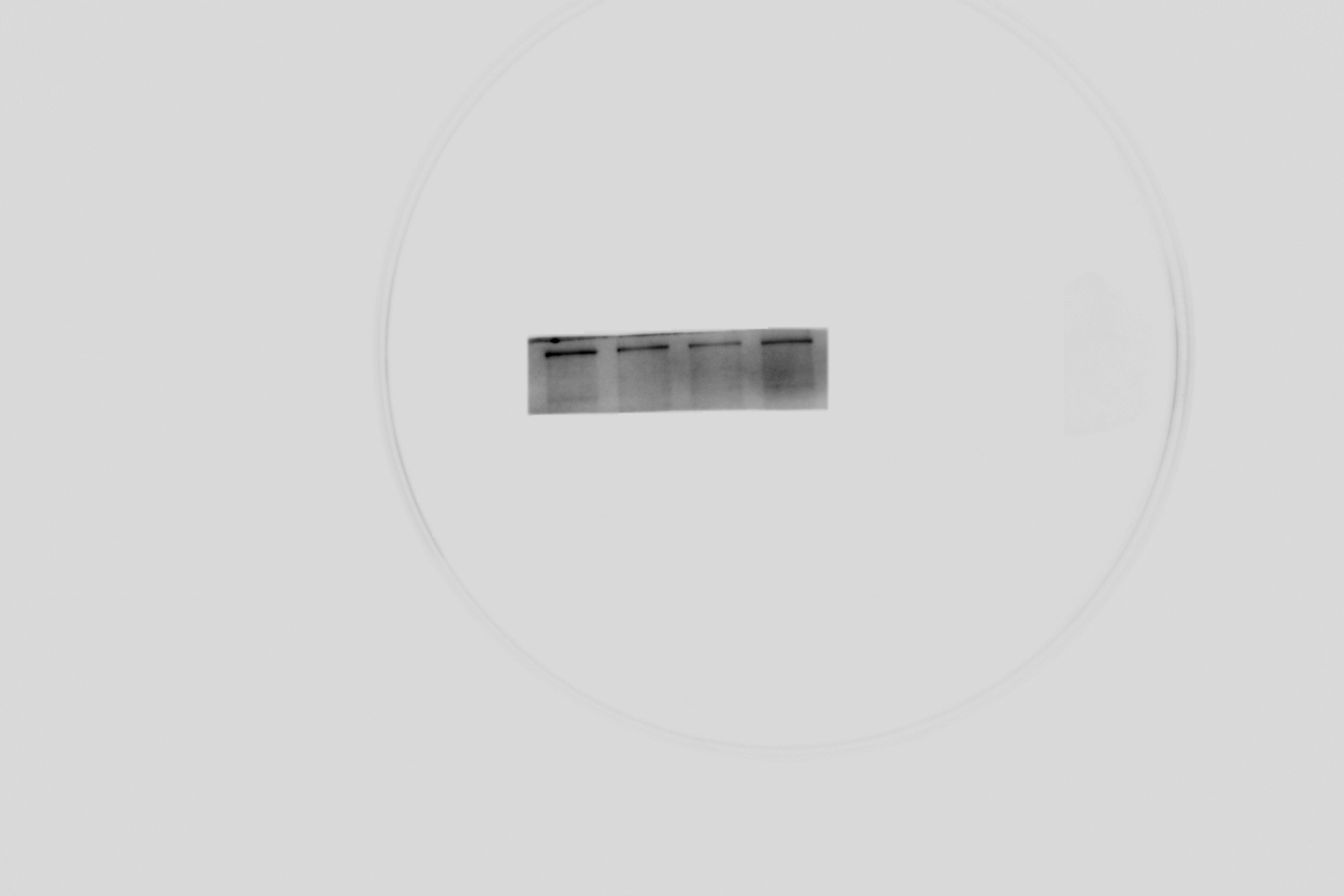

Supplement: S14 Fig — (TIF) [file pone.0153919.s014.tif]

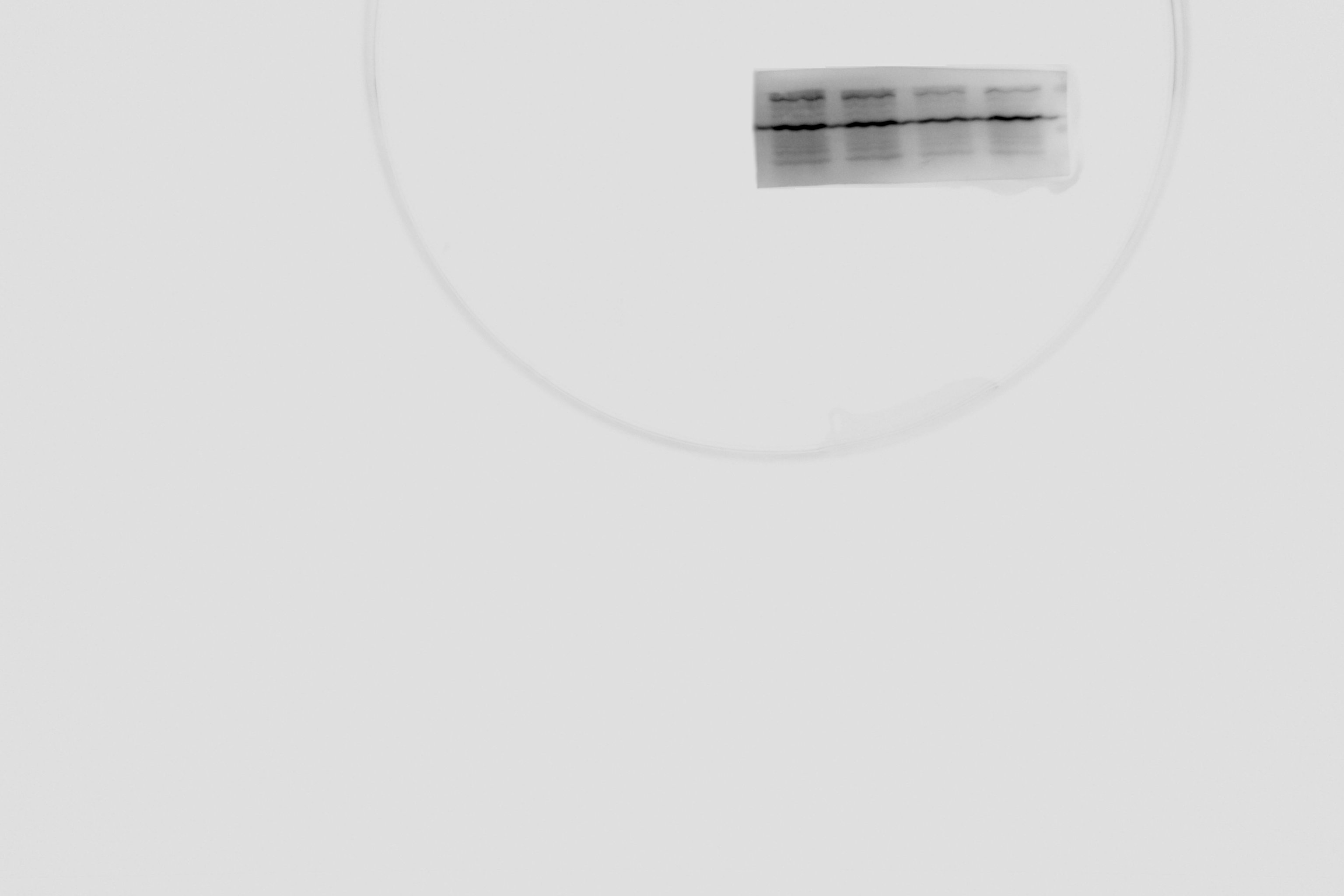

Supplement: S15 Fig — (TIF) [file pone.0153919.s015.tif]

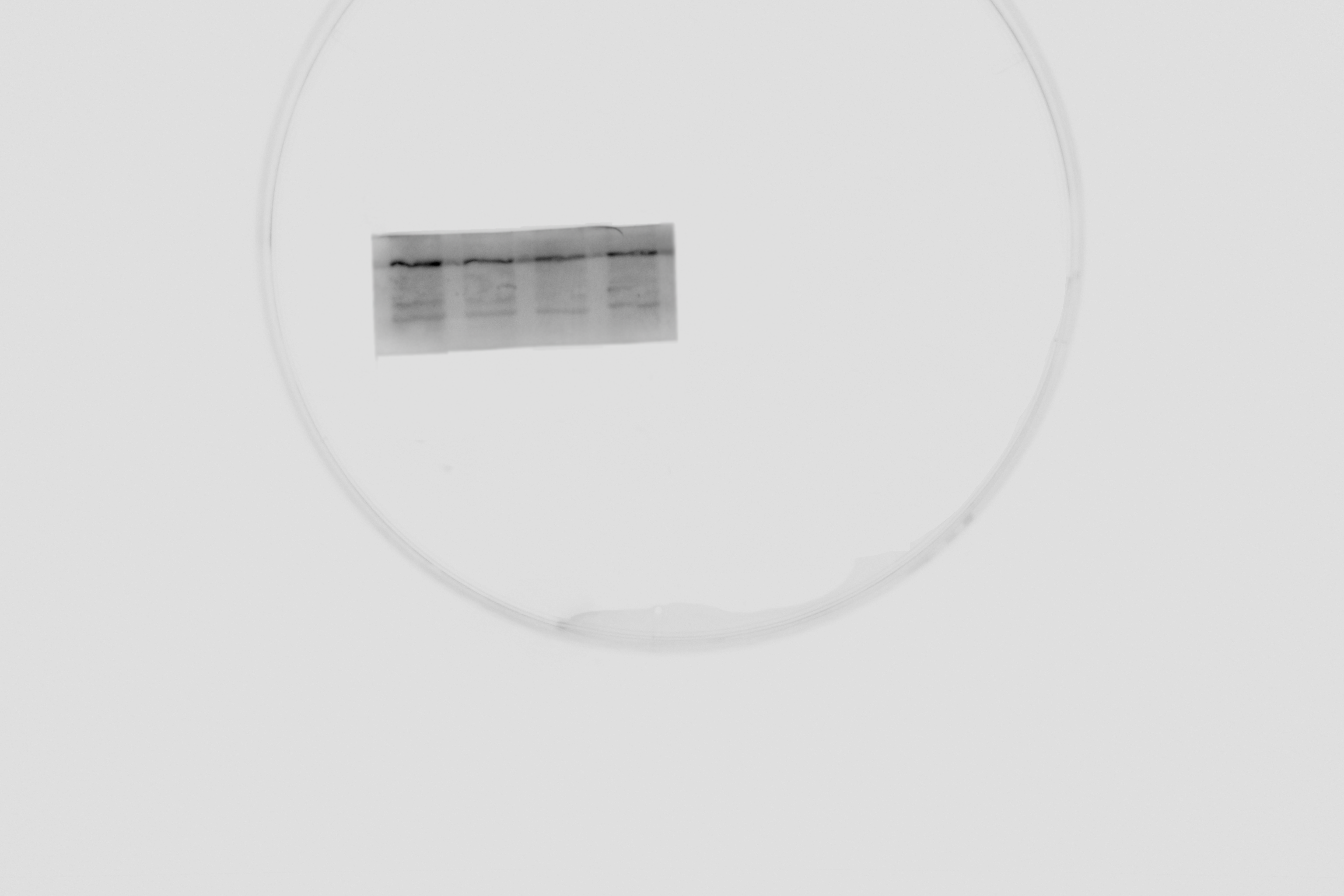

Supplement: S16 Fig — (TIF) [file pone.0153919.s016.tif]

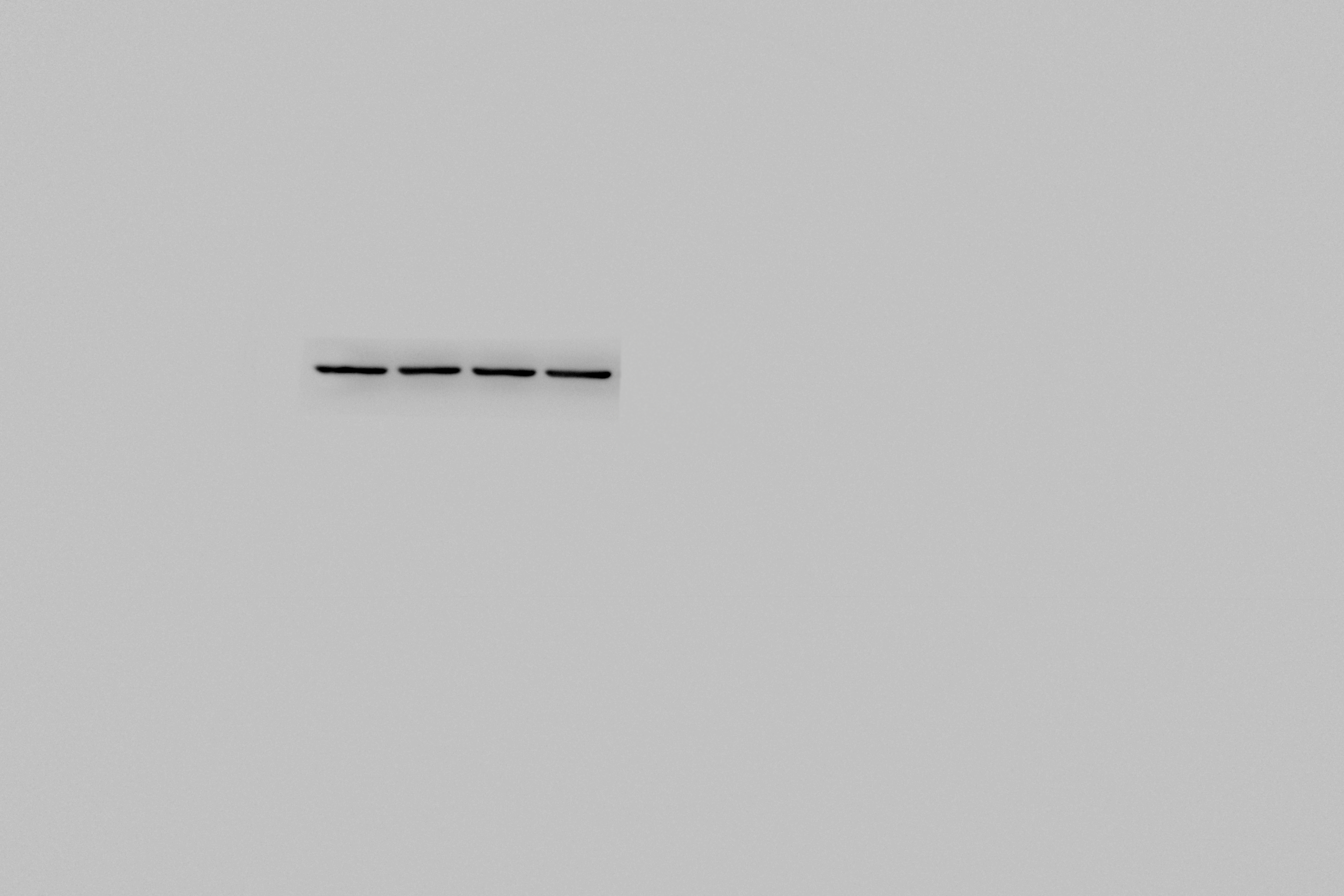

Supplement: S17 Fig — (TIF) [file pone.0153919.s017.tif]

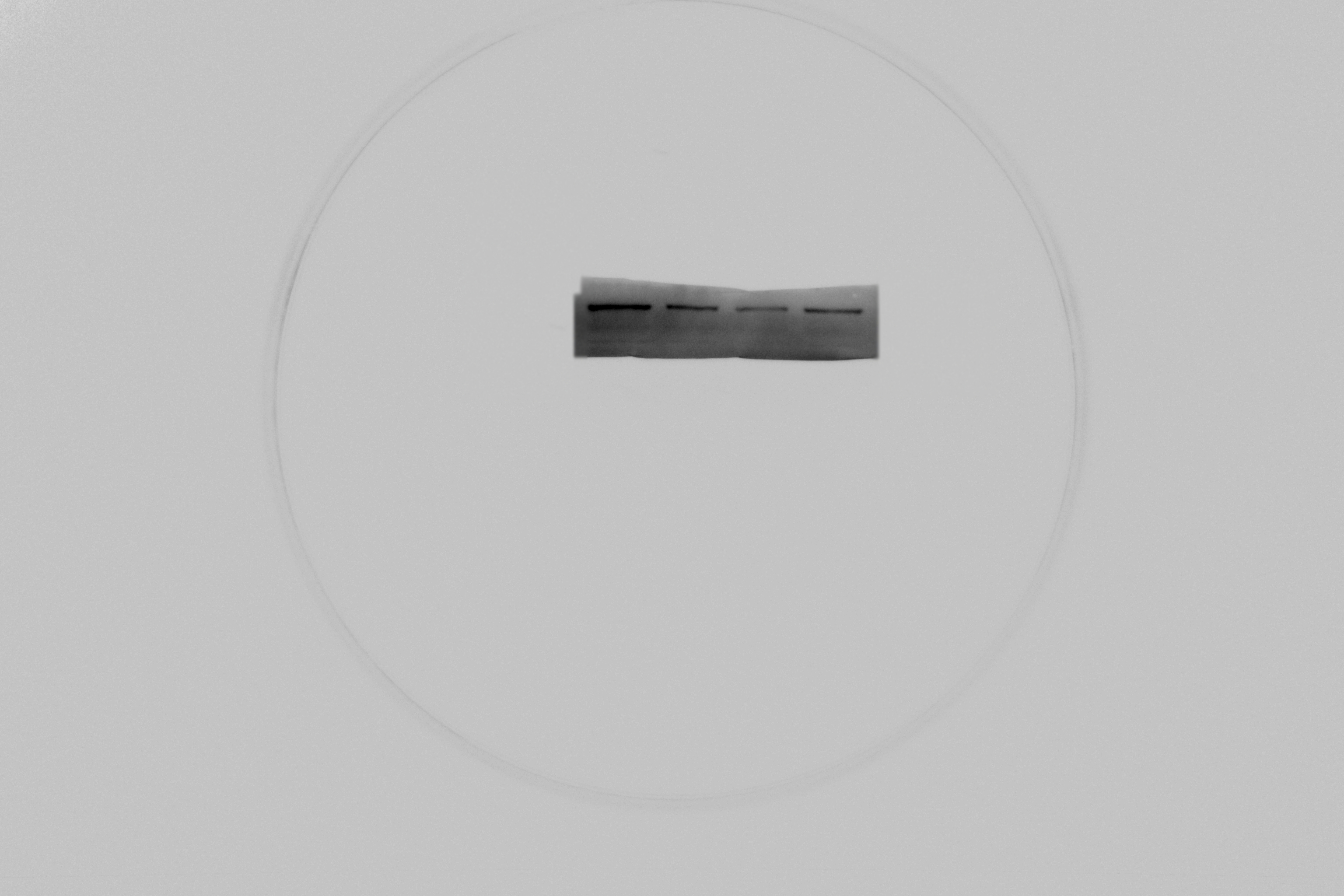

Supplement: S18 Fig — (TIF) [file pone.0153919.s018.tif]

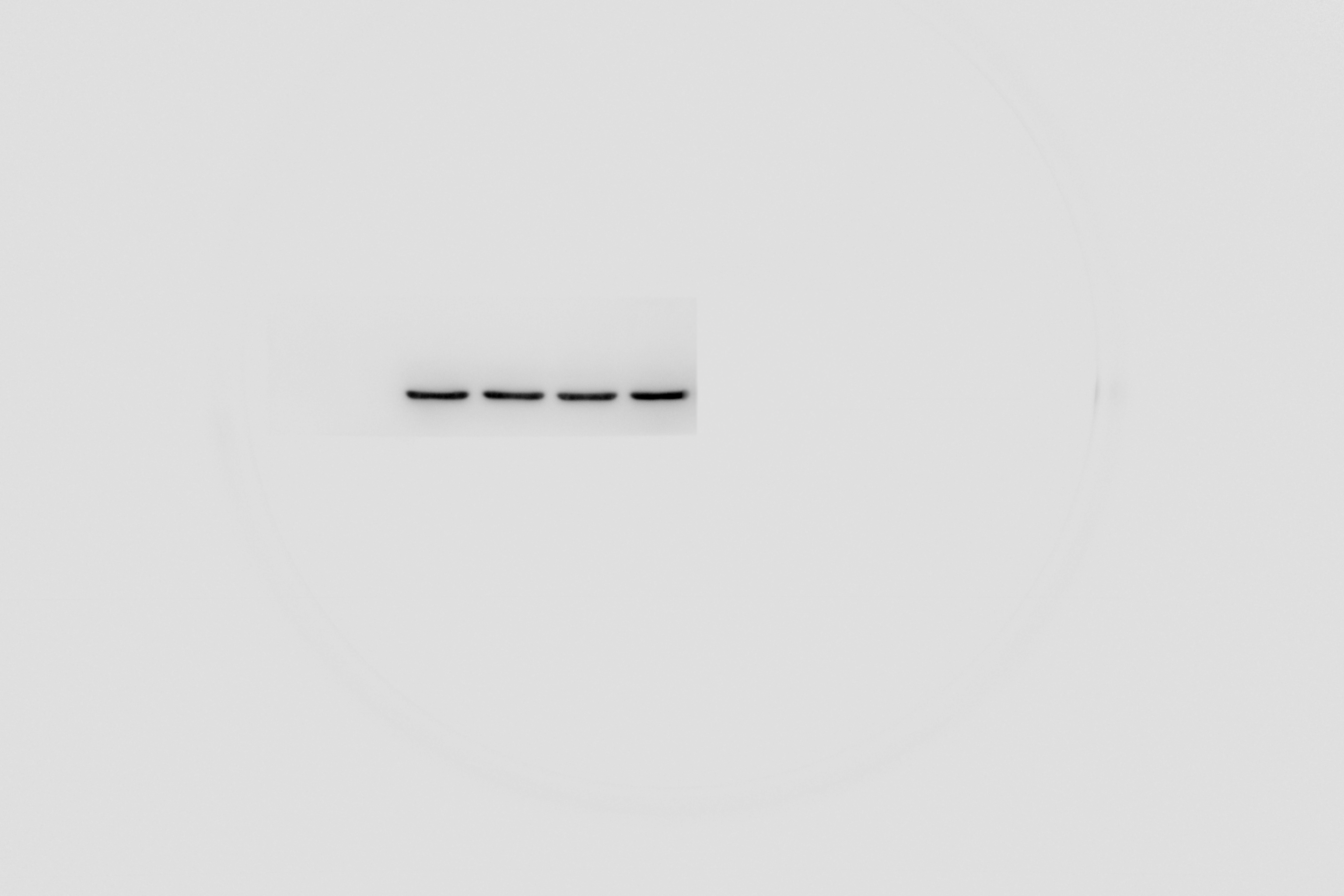

Supplement: S19 Fig — (TIF) [file pone.0153919.s019.tif]

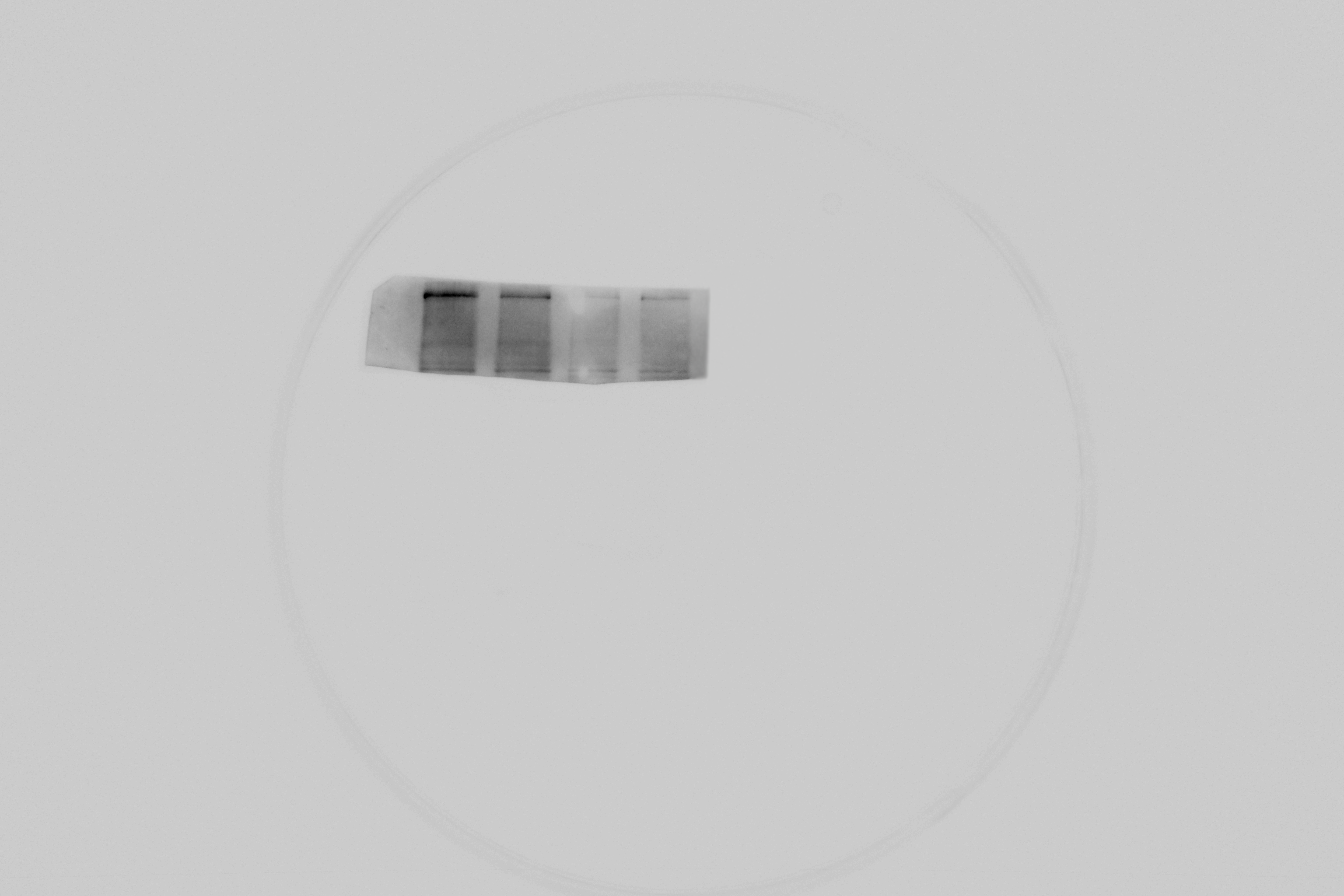

Supplement: S20 Fig — (TIF) [file pone.0153919.s020.tif]

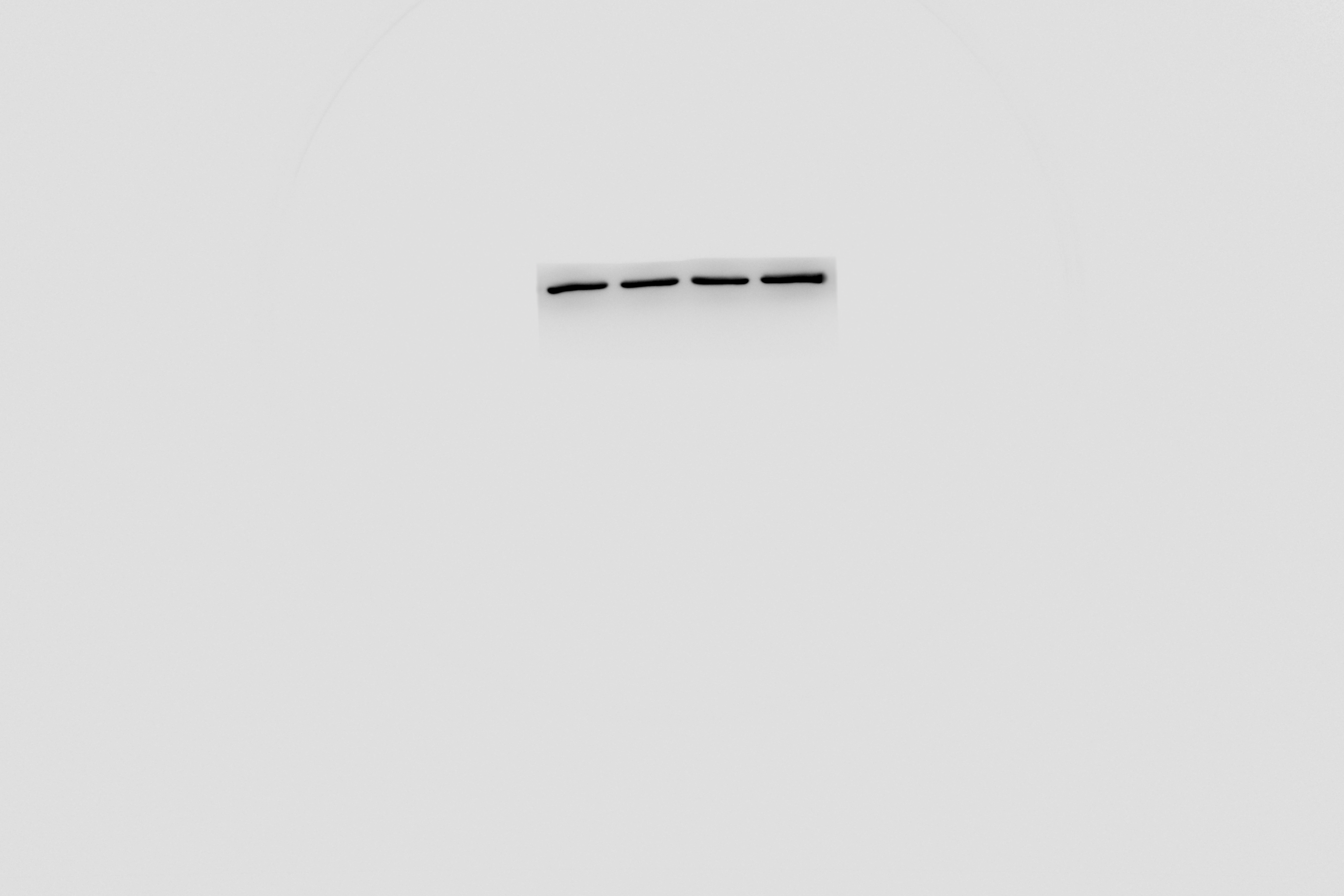

Supplement: S21 Fig — (TIF) [file pone.0153919.s021.tif]

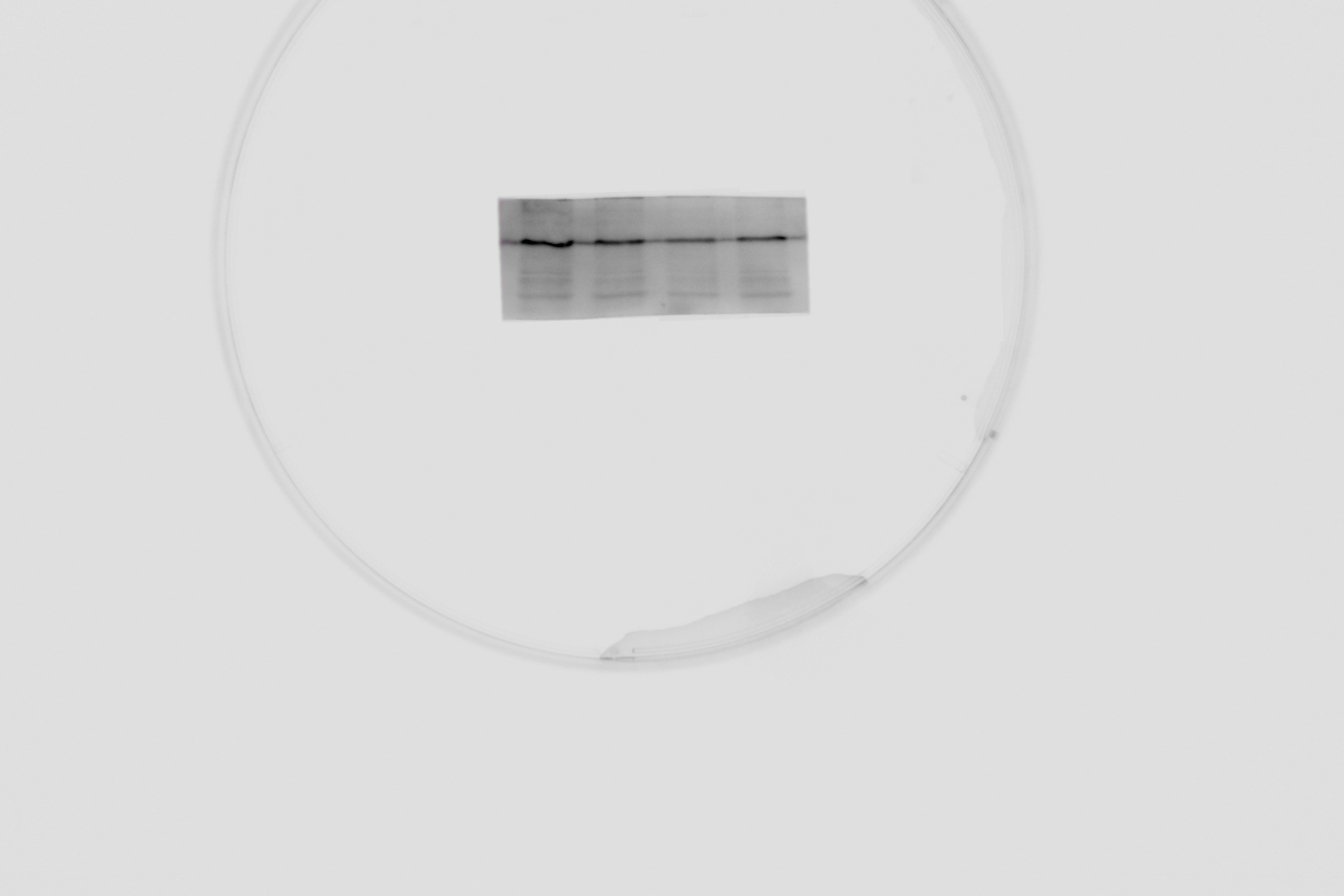

Supplement: S22 Fig — (TIF) [file pone.0153919.s022.tif]

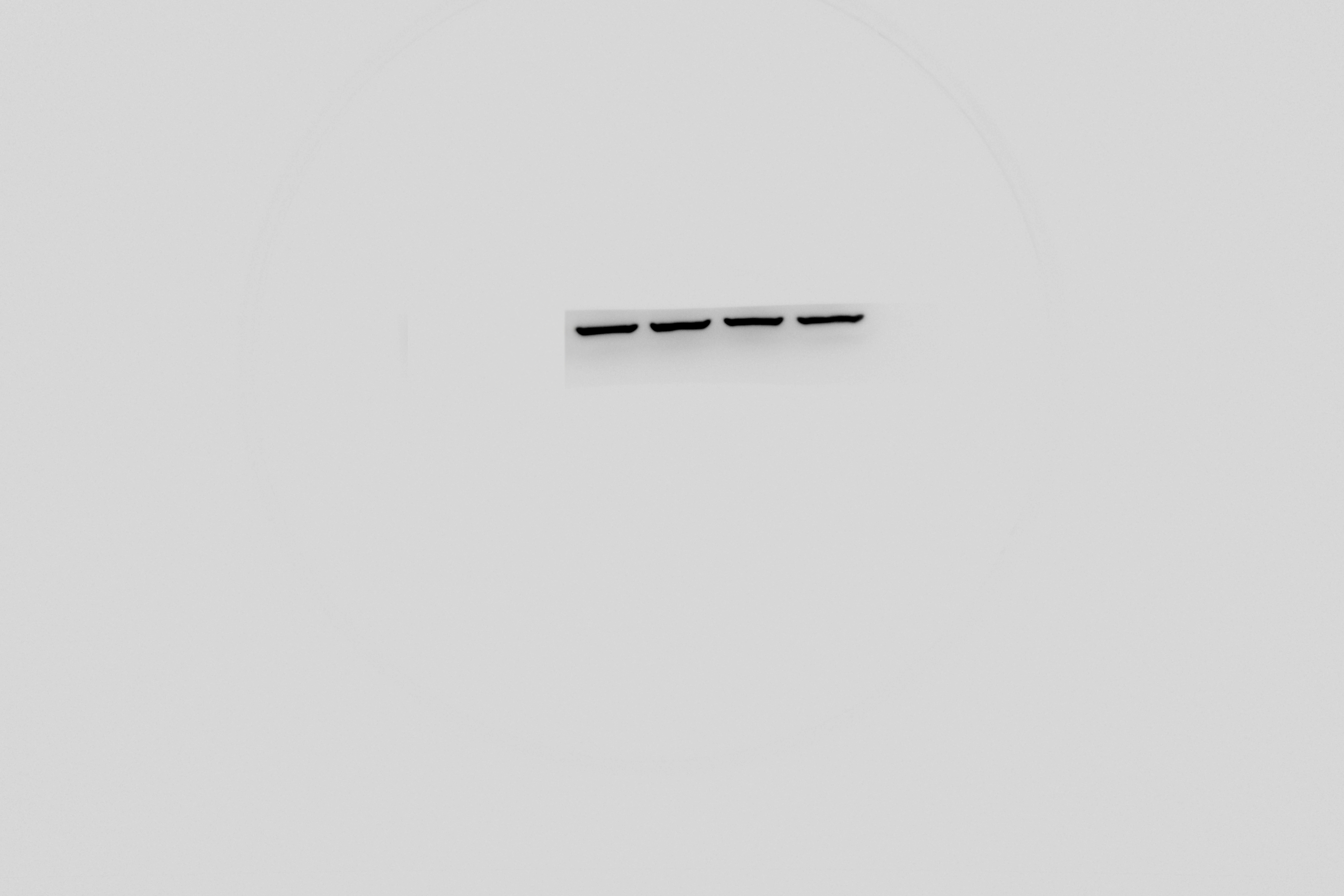

Supplement: S23 Fig — (TIF) [file pone.0153919.s023.tif]

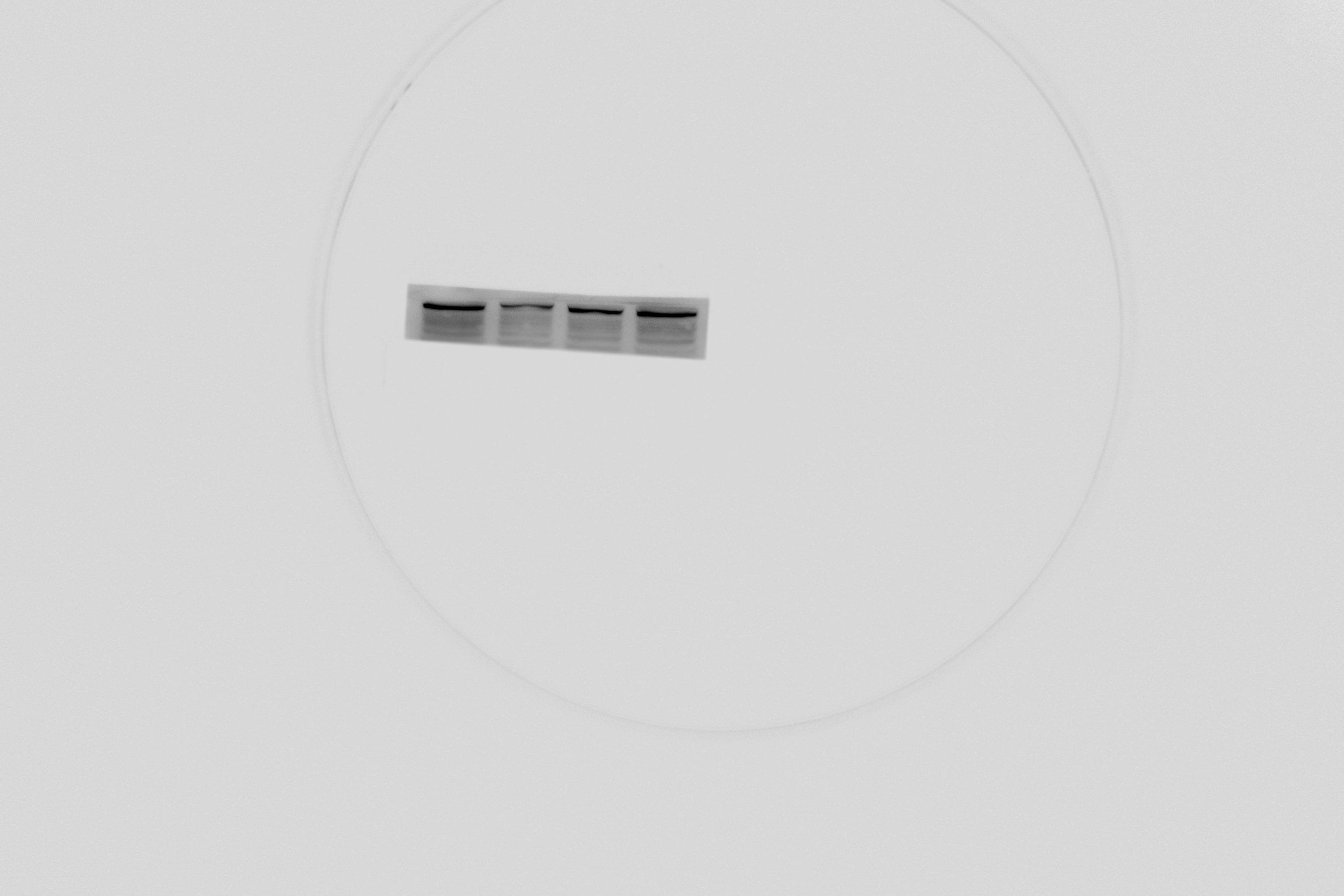

Supplement: S24 Fig — (TIF) [file pone.0153919.s024.tif]

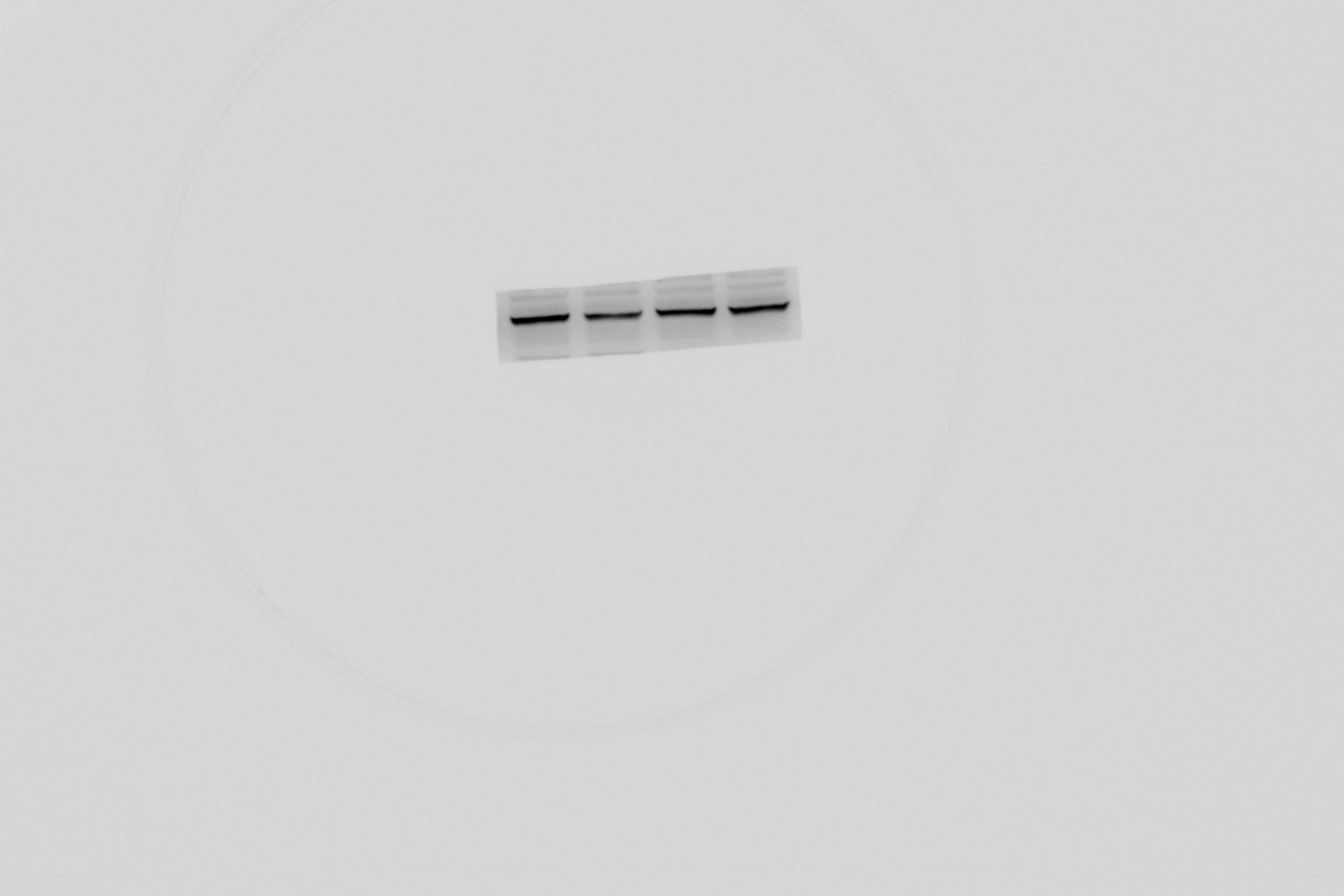

Supplement: S25 Fig — (TIF) [file pone.0153919.s025.tif]

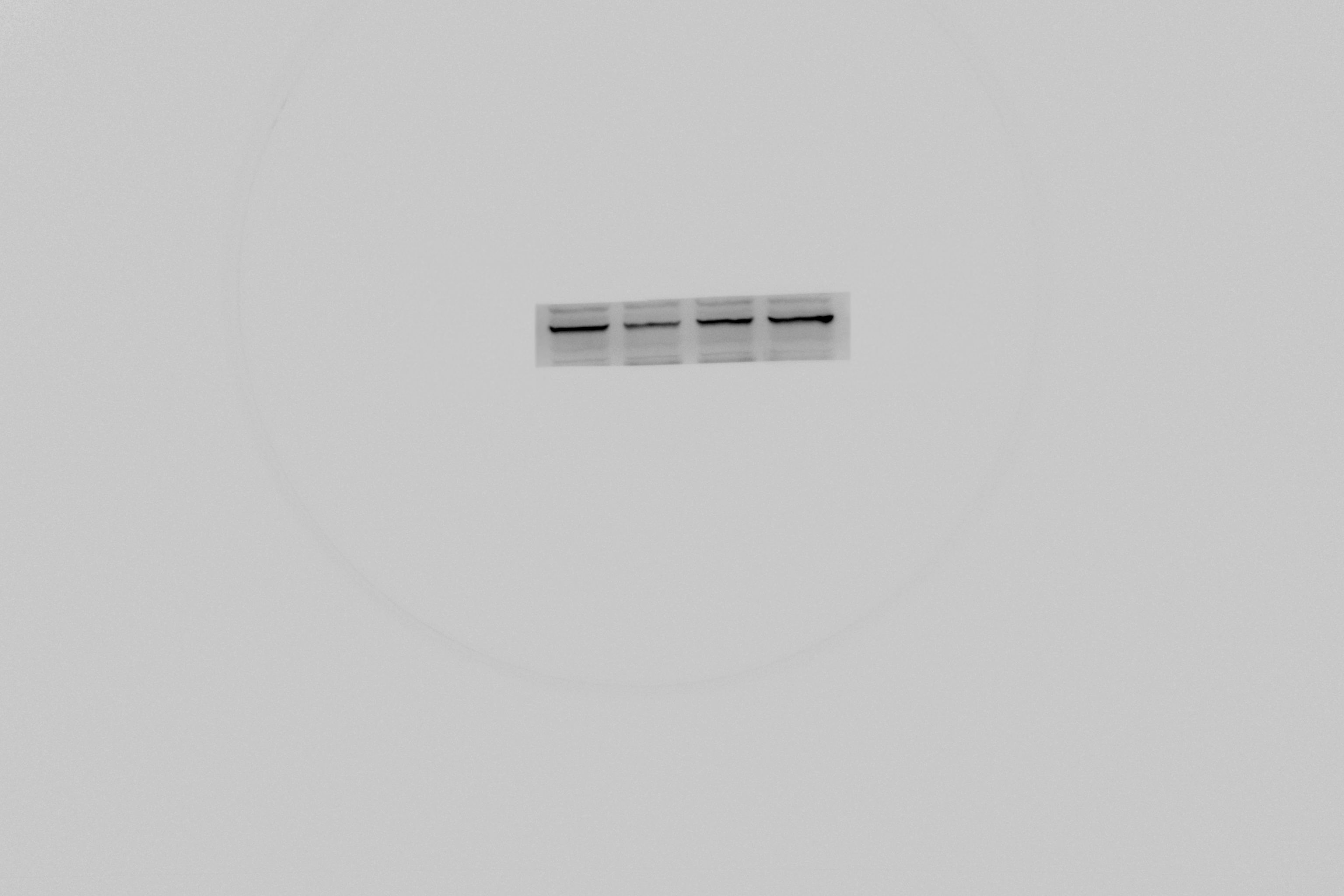

Supplement: S26 Fig — (TIF) [file pone.0153919.s026.tif]

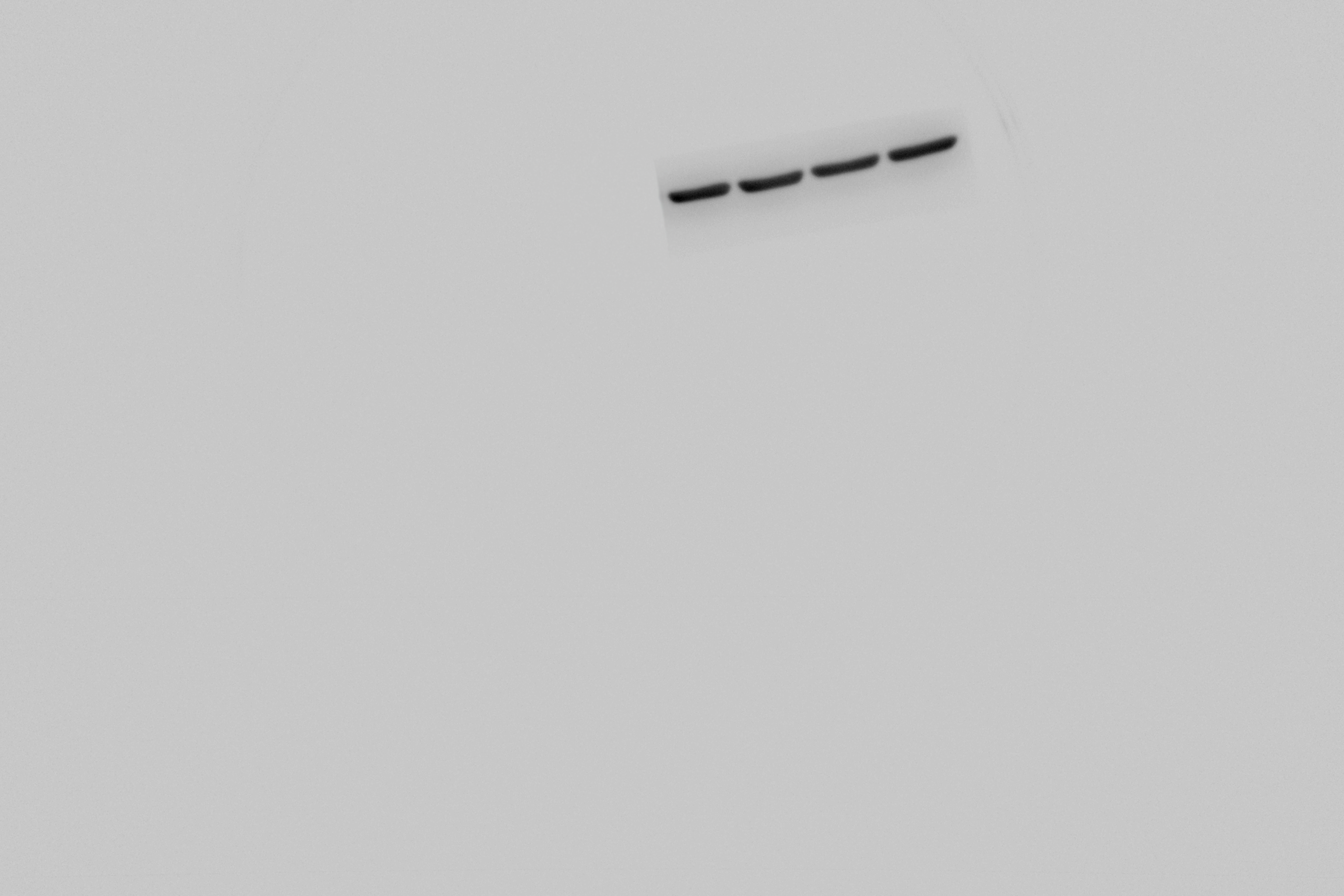

Supplement: S27 Fig — (TIF) [file pone.0153919.s027.tif]

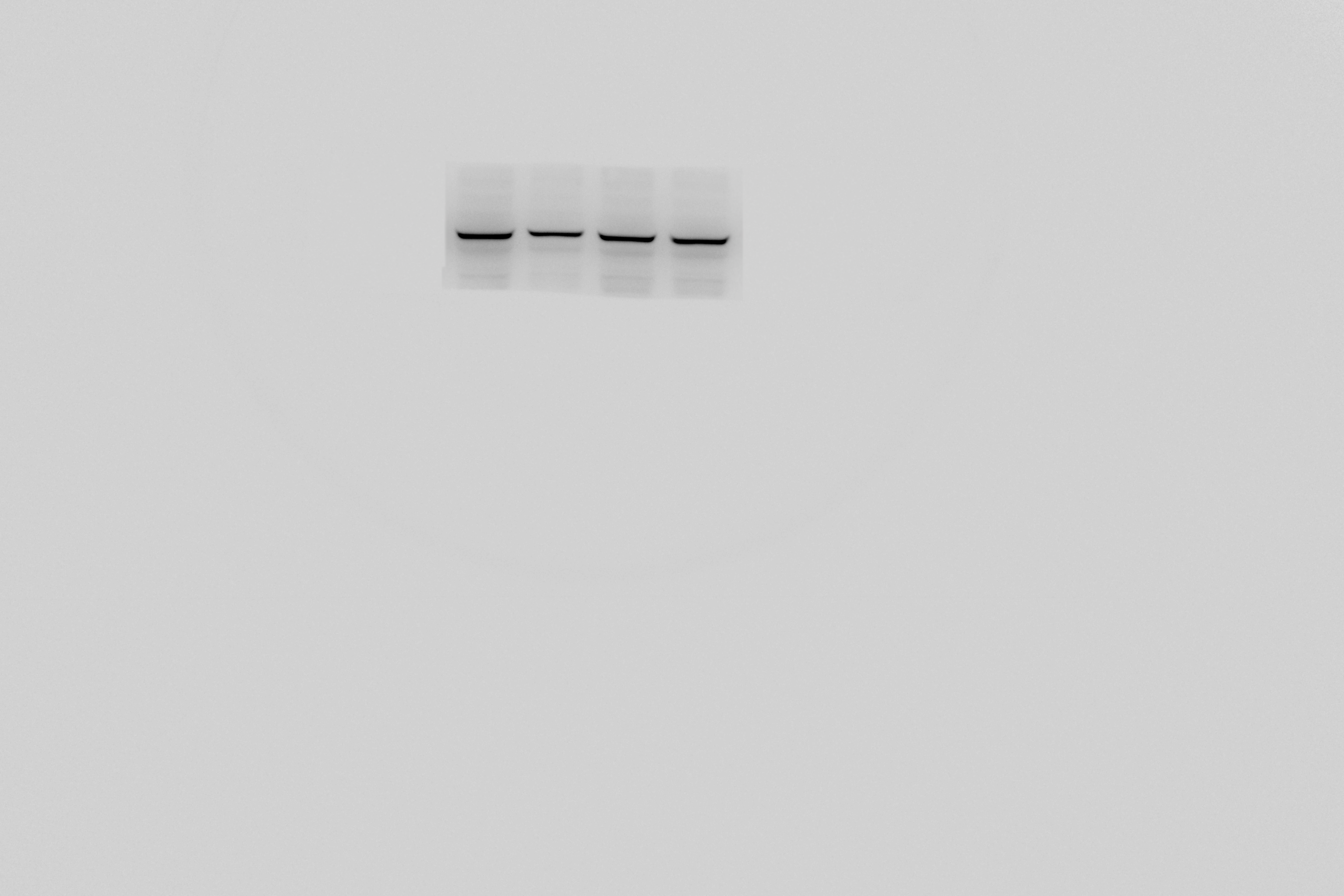

Supplement: S28 Fig — (TIF) [file pone.0153919.s028.tif]

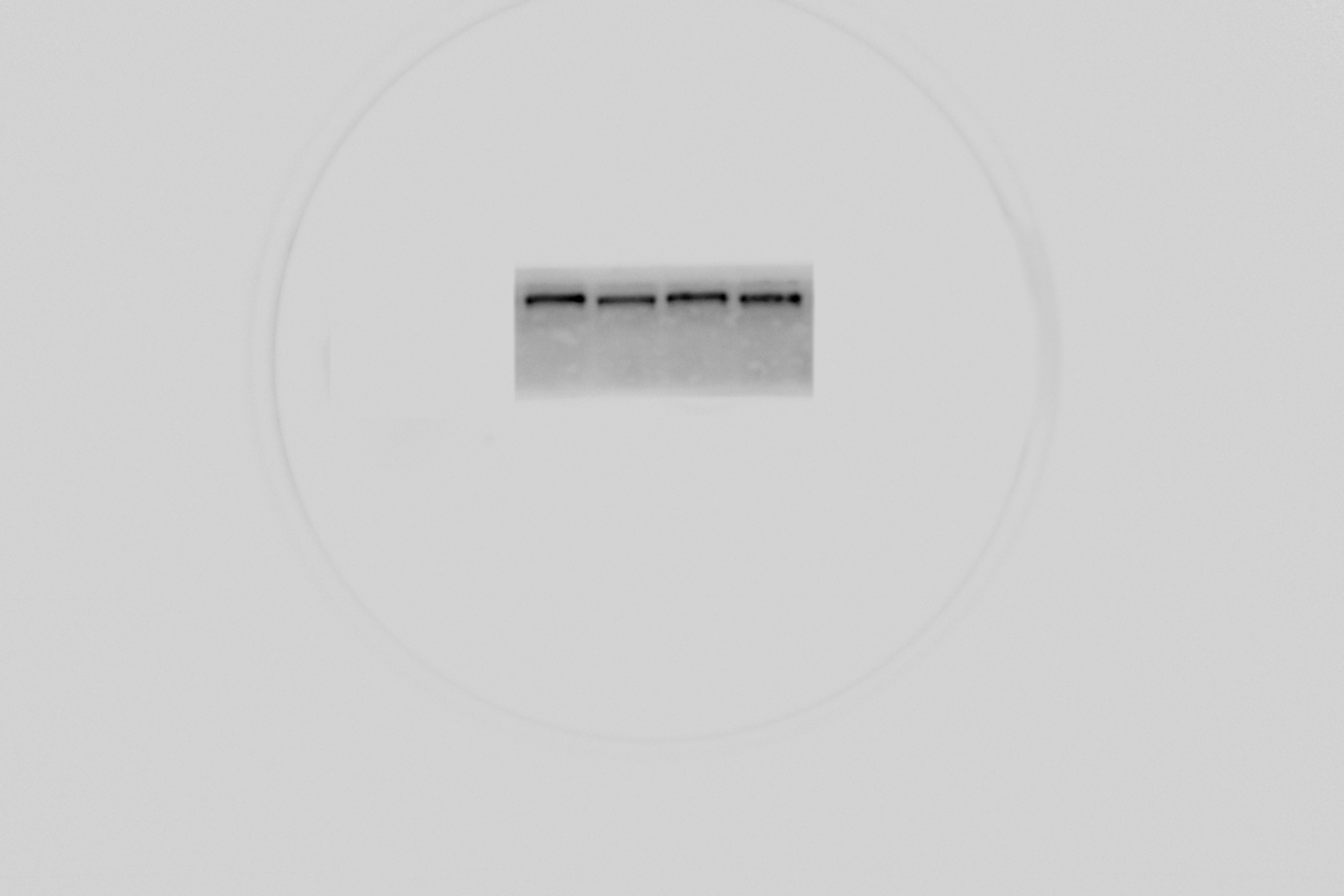

Supplement: S29 Fig — (TIF) [file pone.0153919.s029.tif]

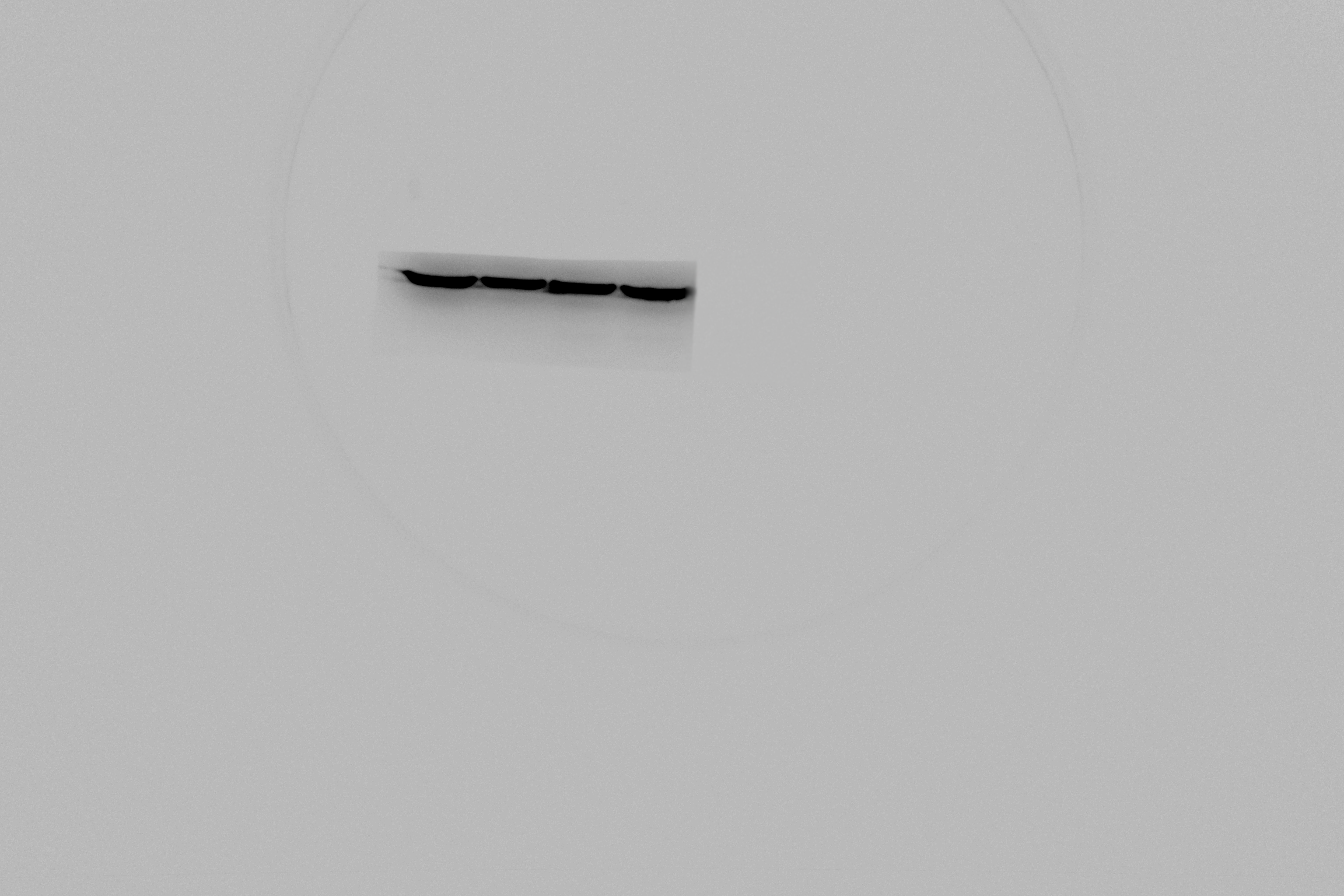

Supplement: S31 Fig — (TIF) [file pone.0153919.s031.tif]

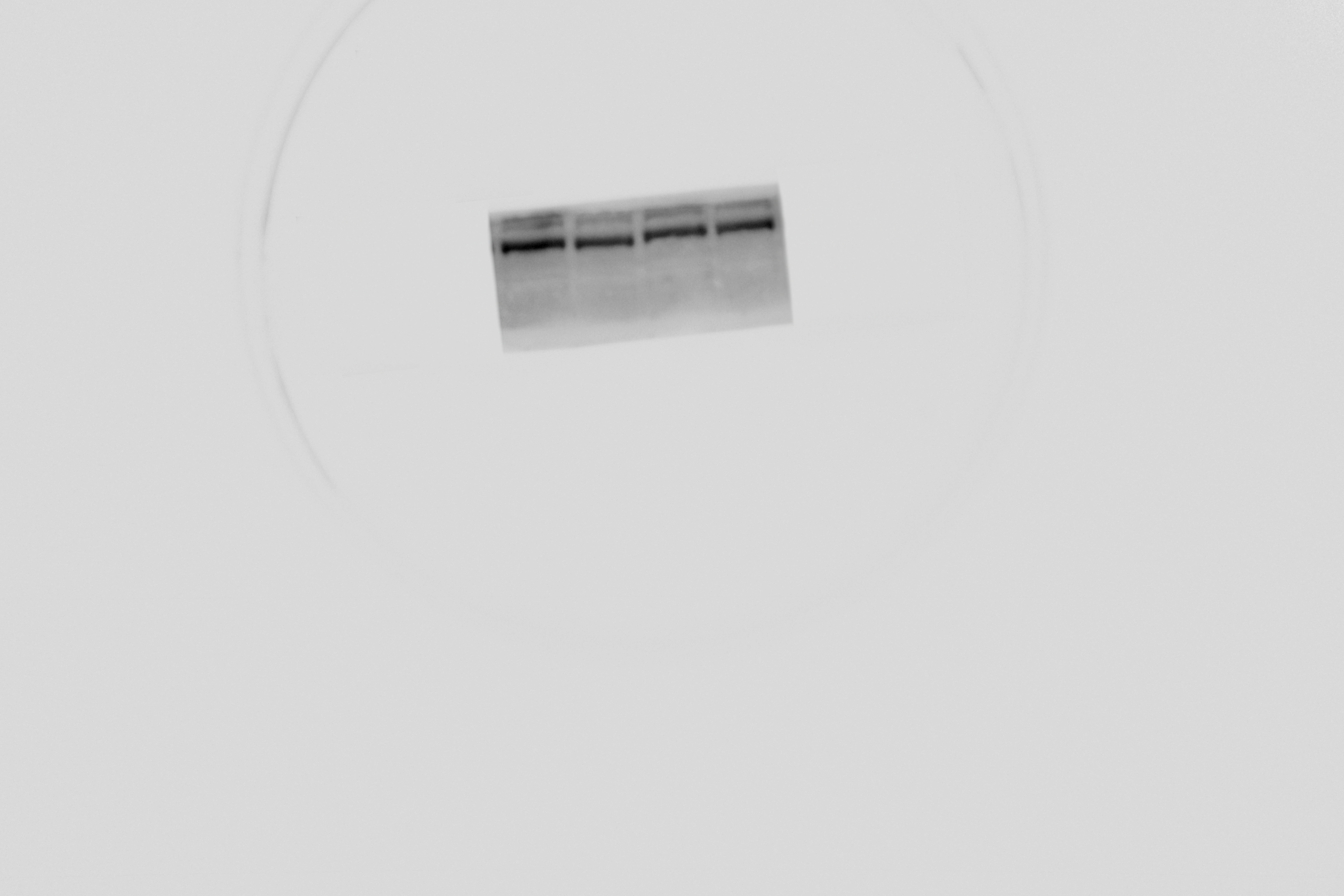

Supplement: S32 Fig — (TIF) [file pone.0153919.s032.tif]

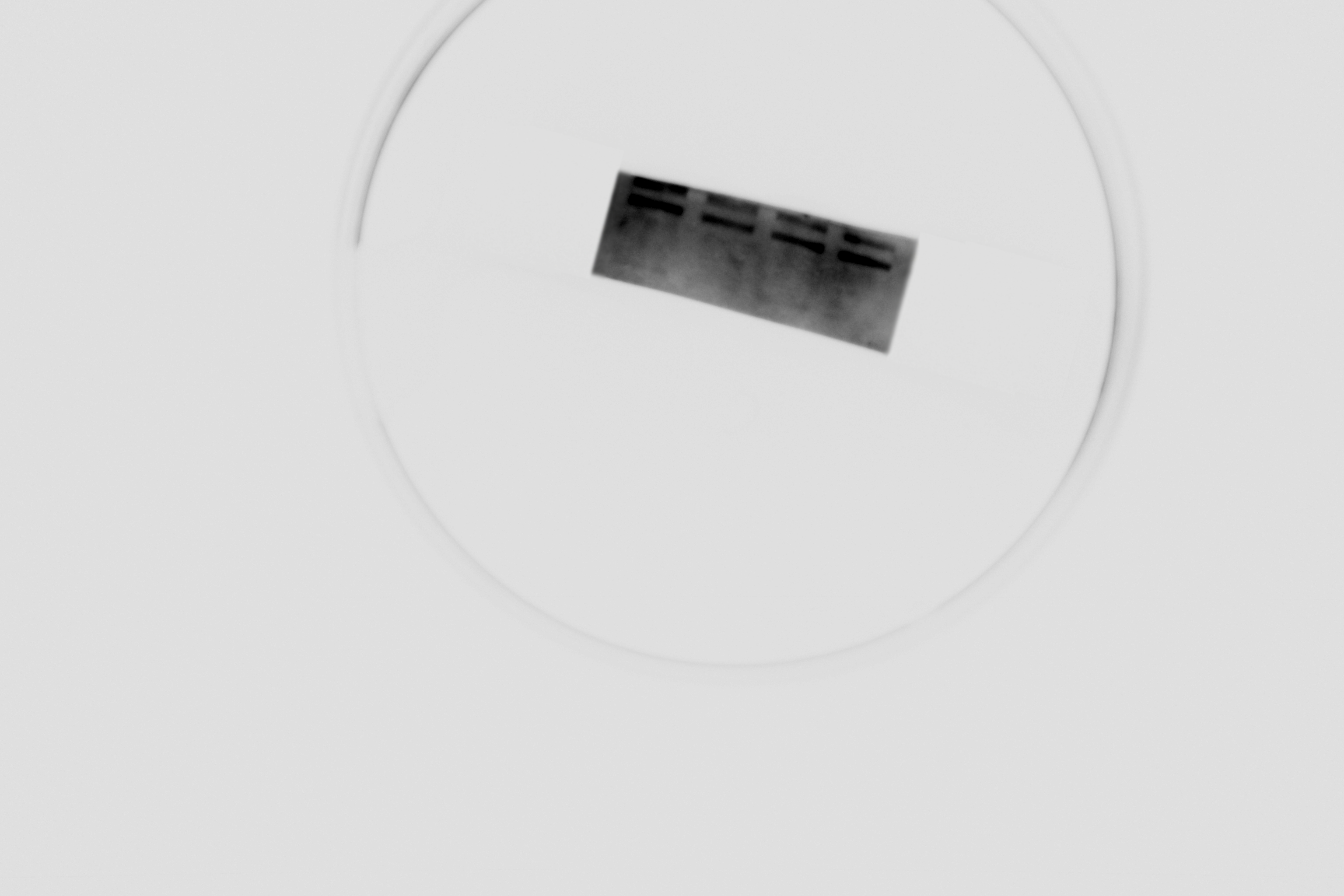

Supplement: S33 Fig — (TIF) [file pone.0153919.s033.tif]

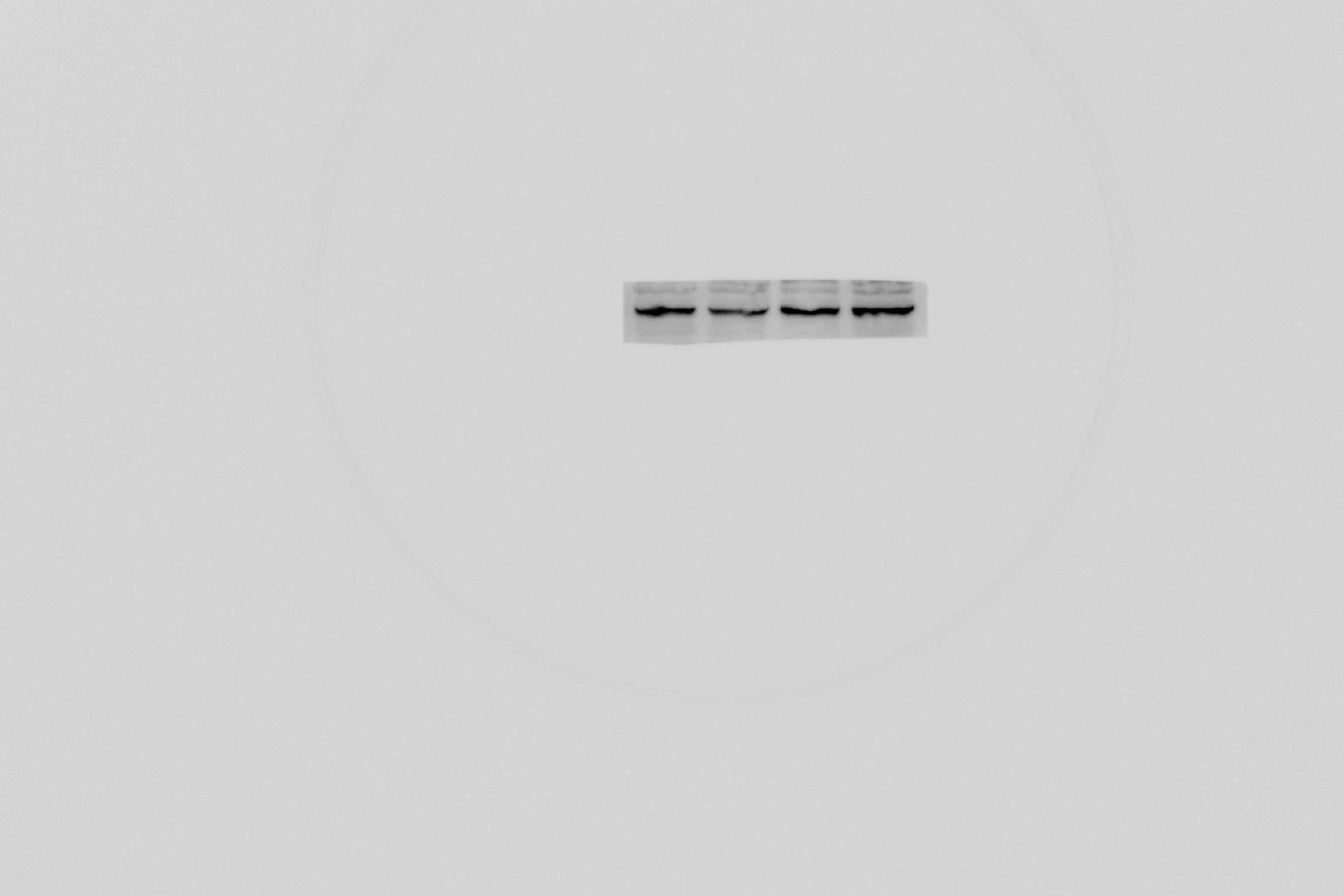

Supplement: S34 Fig — (TIF) [file pone.0153919.s034.tif]

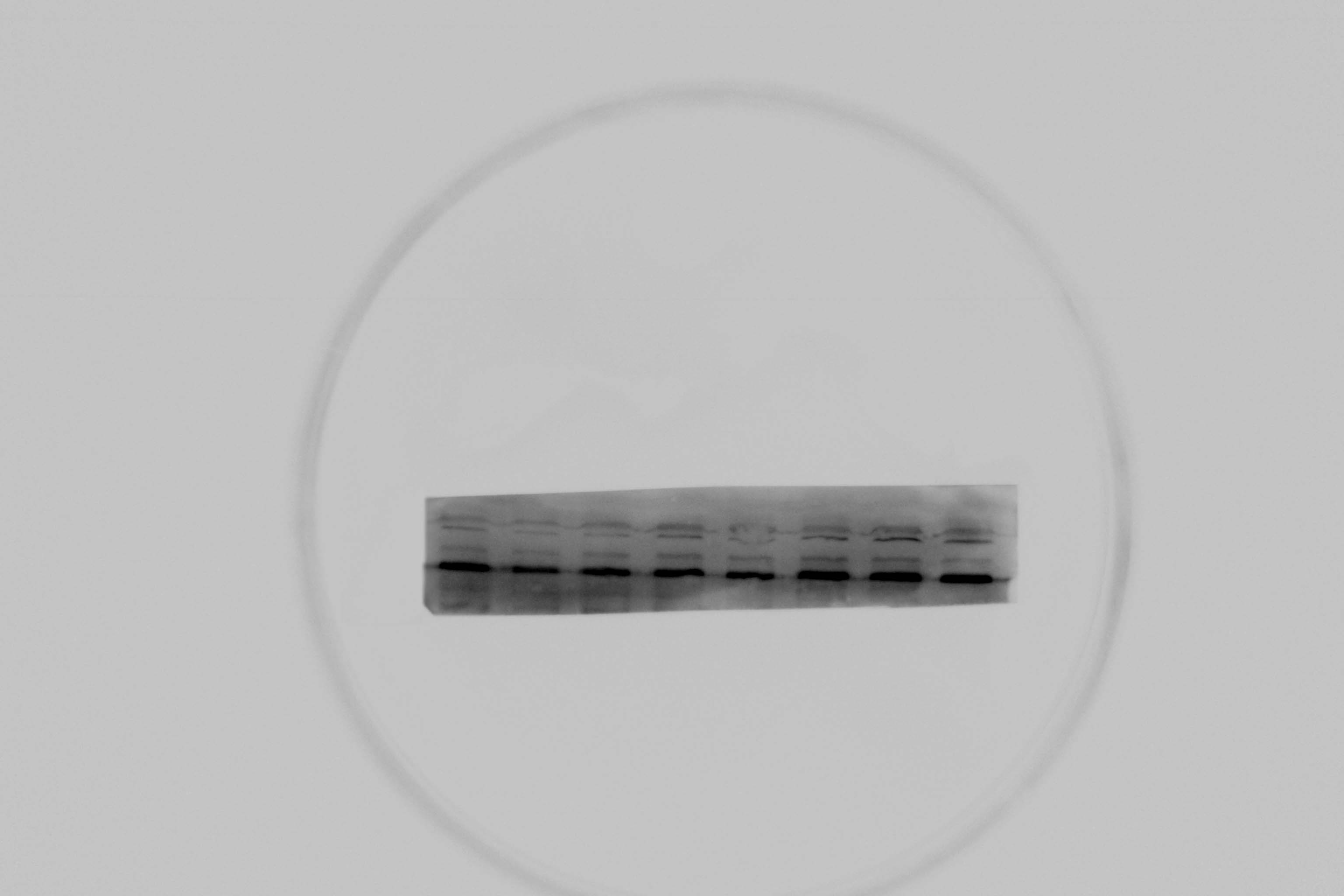

Supplement: S36 Fig — (TIF) [file pone.0153919.s036.tif]

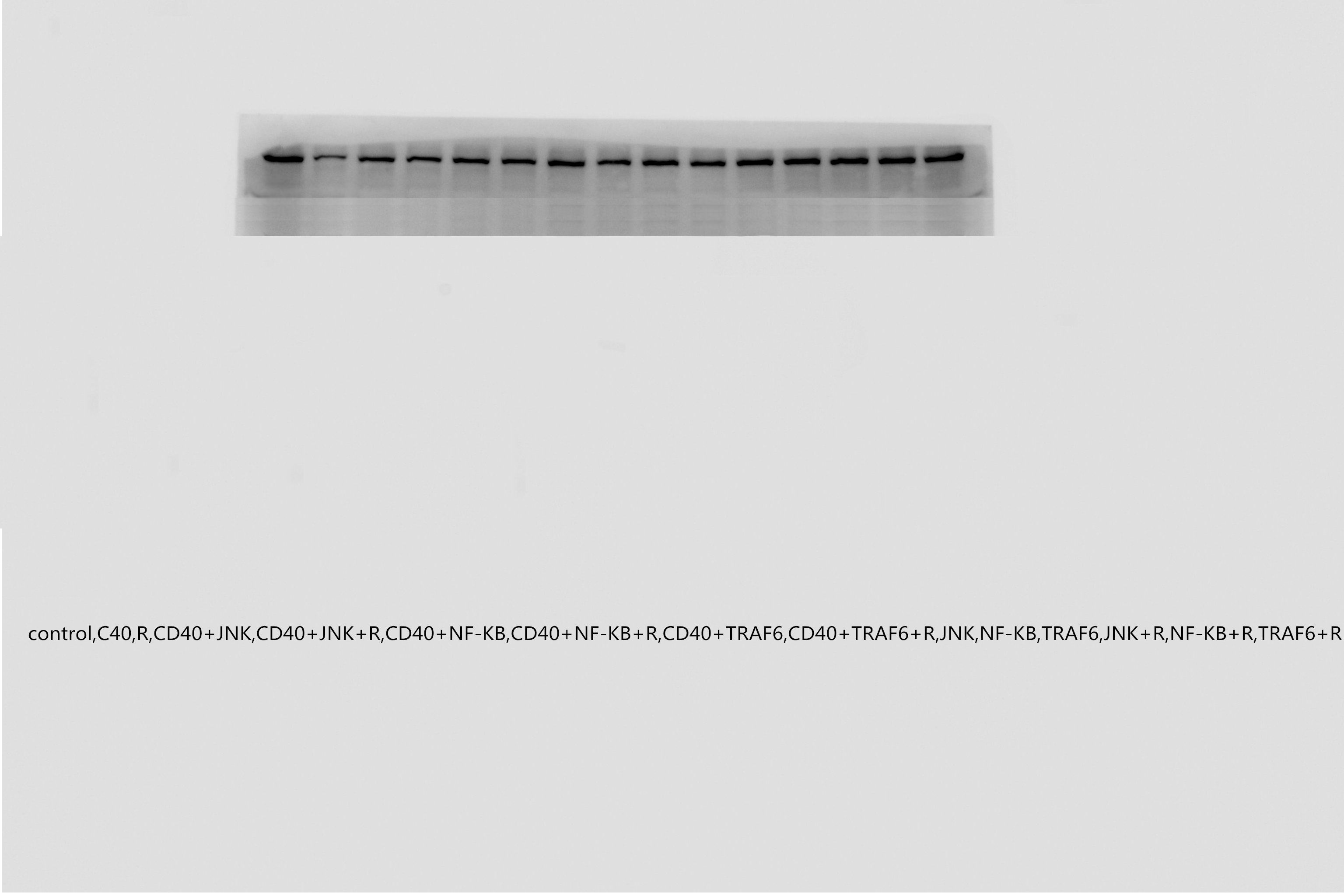

Supplement: S38 Fig — (TIF) [file pone.0153919.s038.tif]

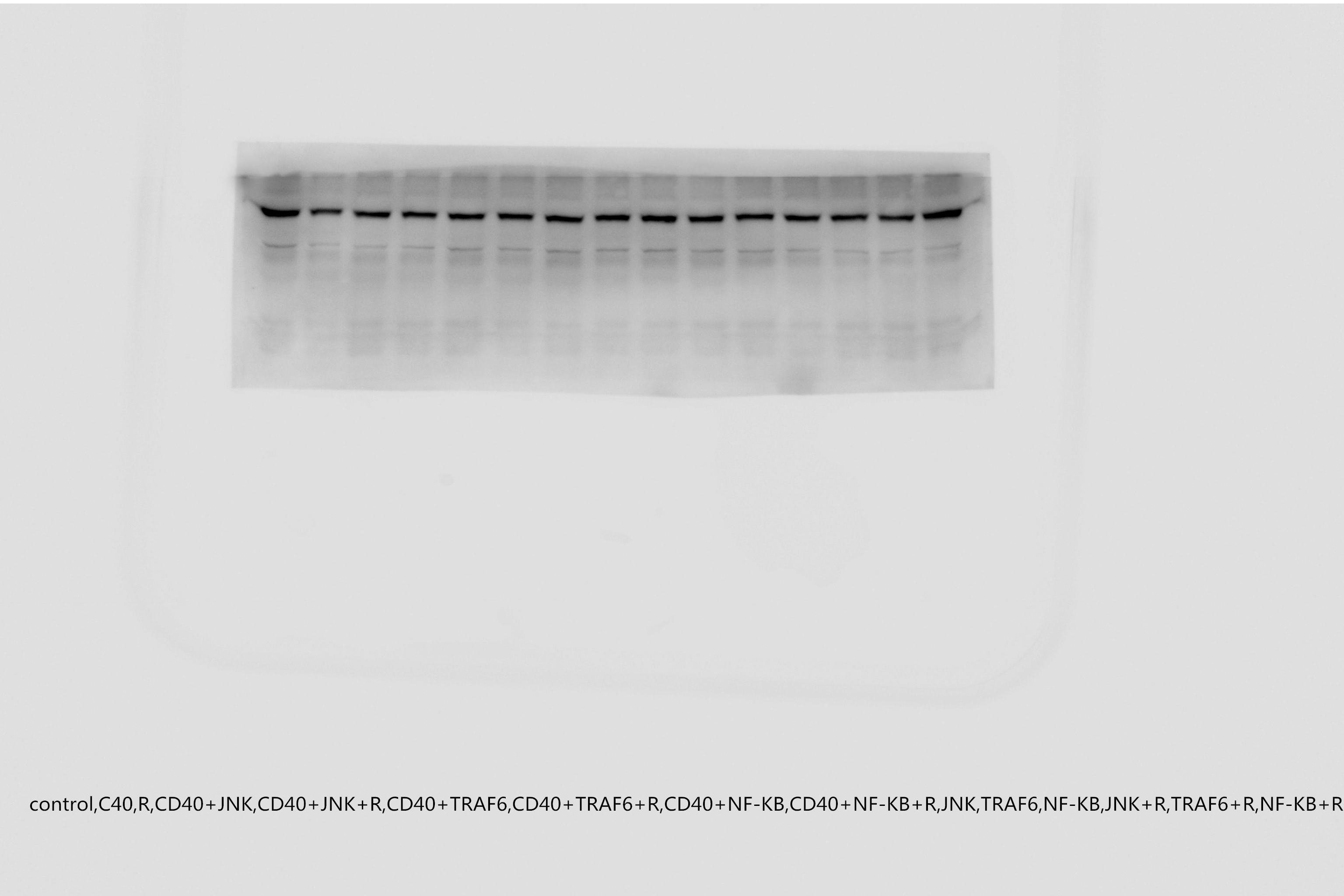

Supplement: S40 Fig — (TIF) [file pone.0153919.s040.tif]

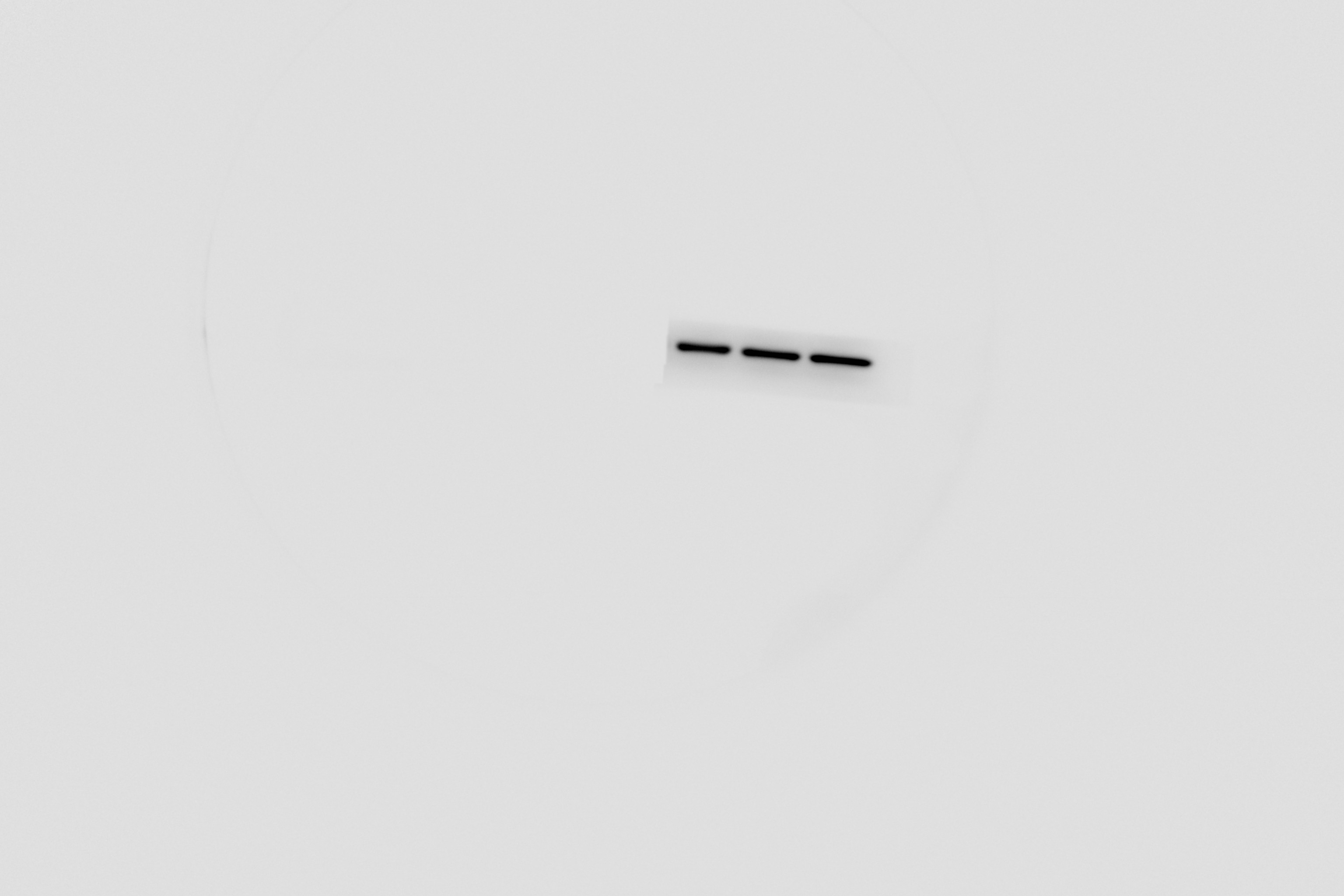

Supplement: S41 Fig — (TIF) [file pone.0153919.s041.tif]

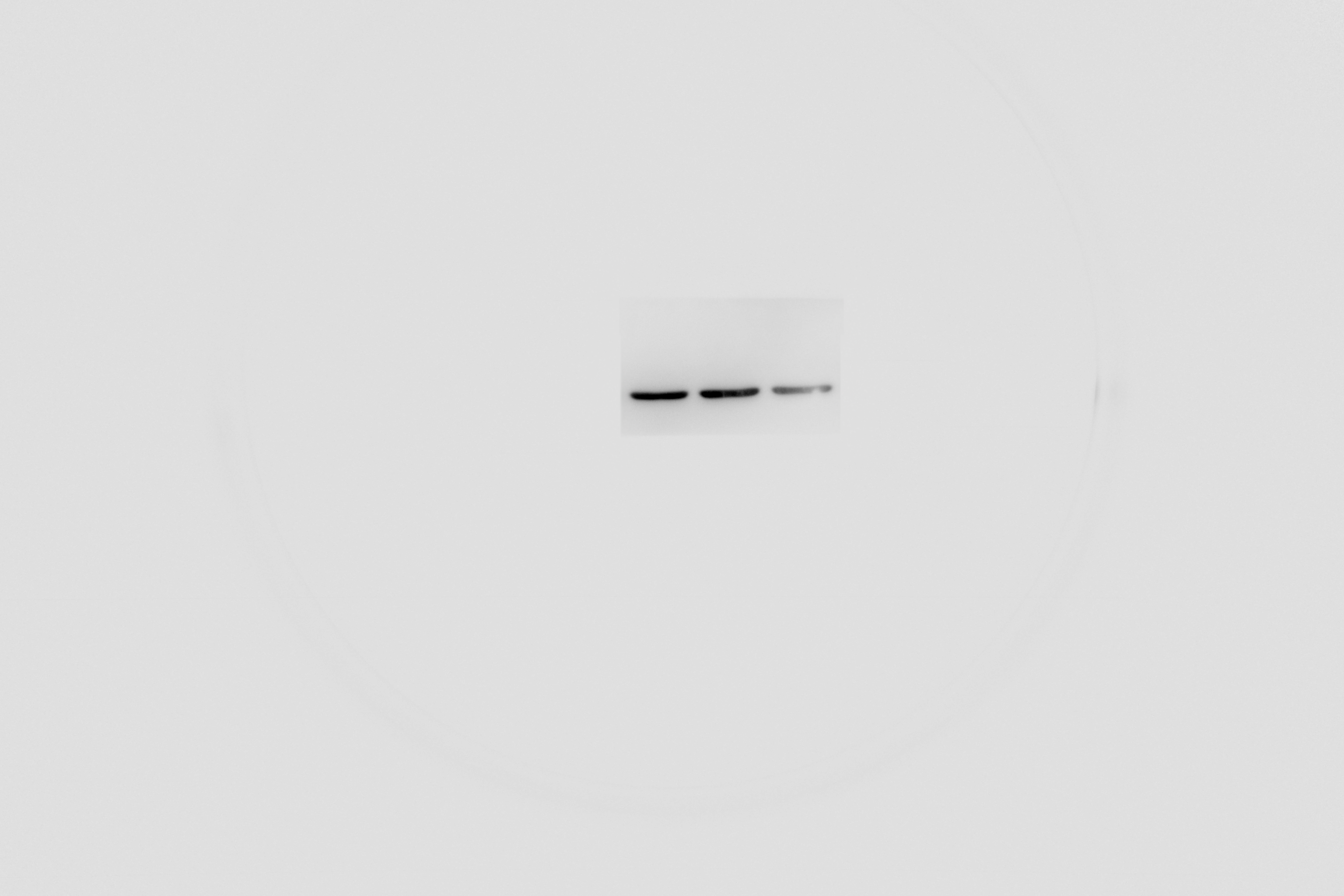

Supplement: S42 Fig — (TIF) [file pone.0153919.s042.tif]

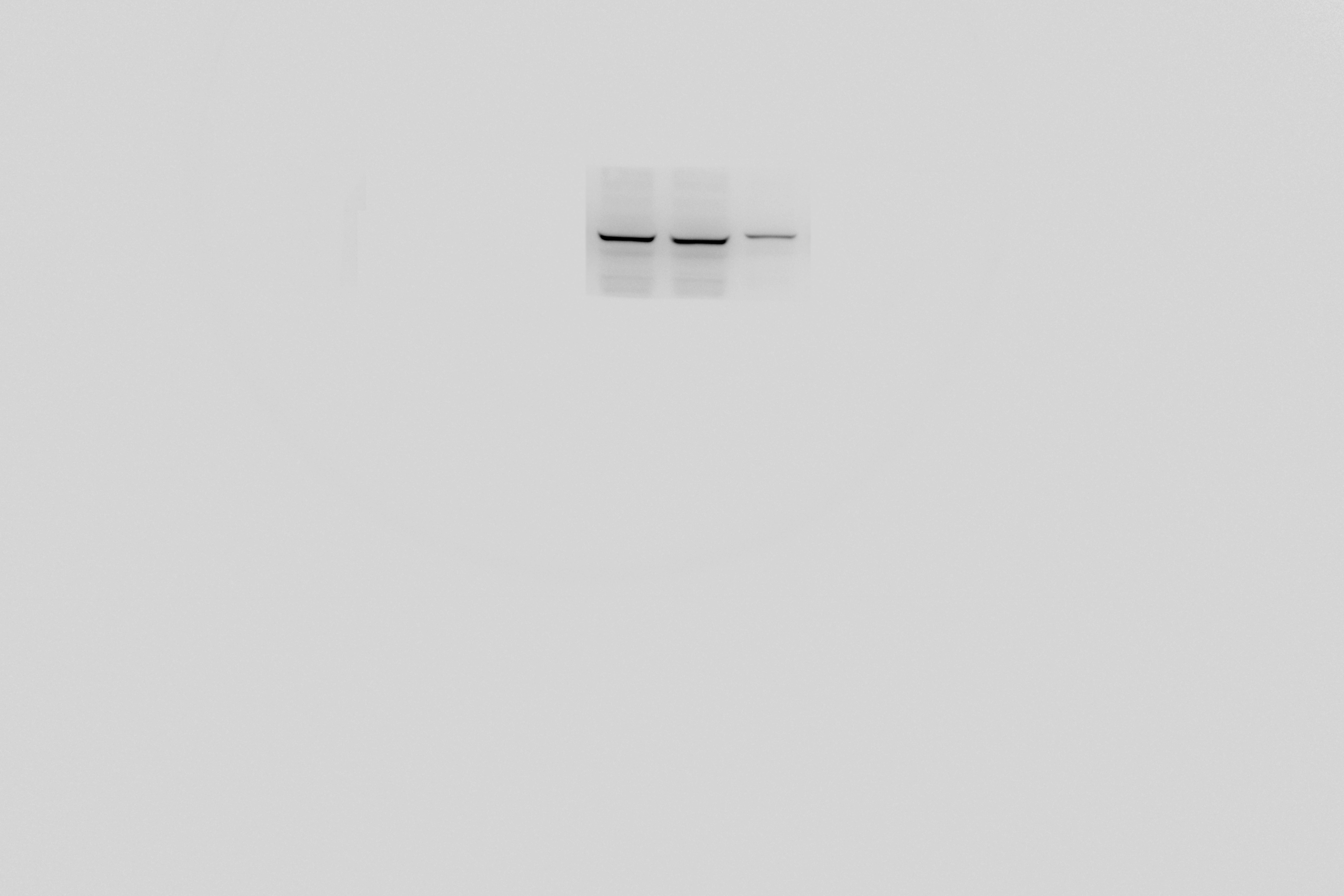

Supplement: S44 Fig — (TIF) [file pone.0153919.s044.tif]

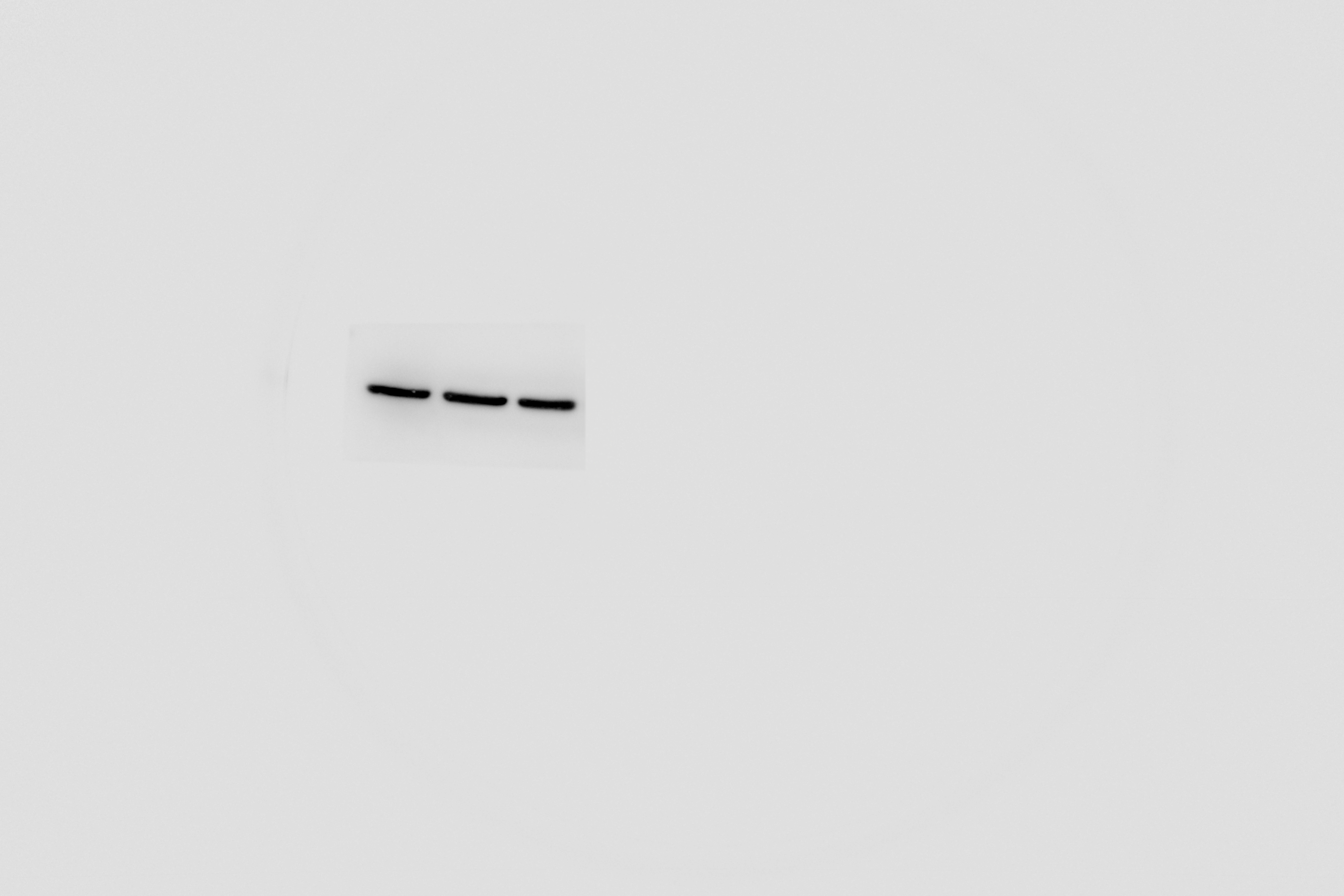

Supplement: S45 Fig — (TIF) [file pone.0153919.s045.tif]

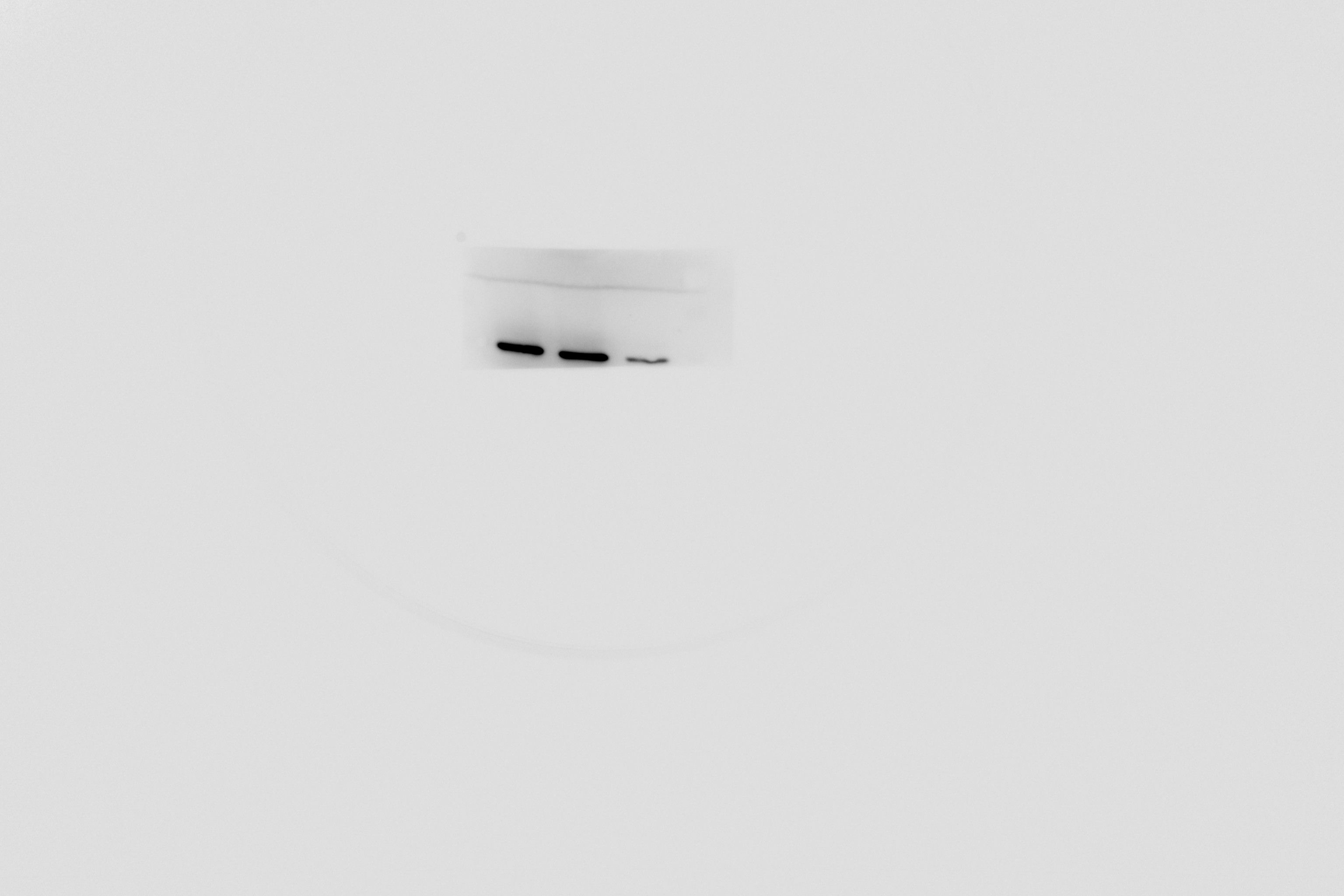

Supplement: S46 Fig — (TIF) [file pone.0153919.s046.tif]

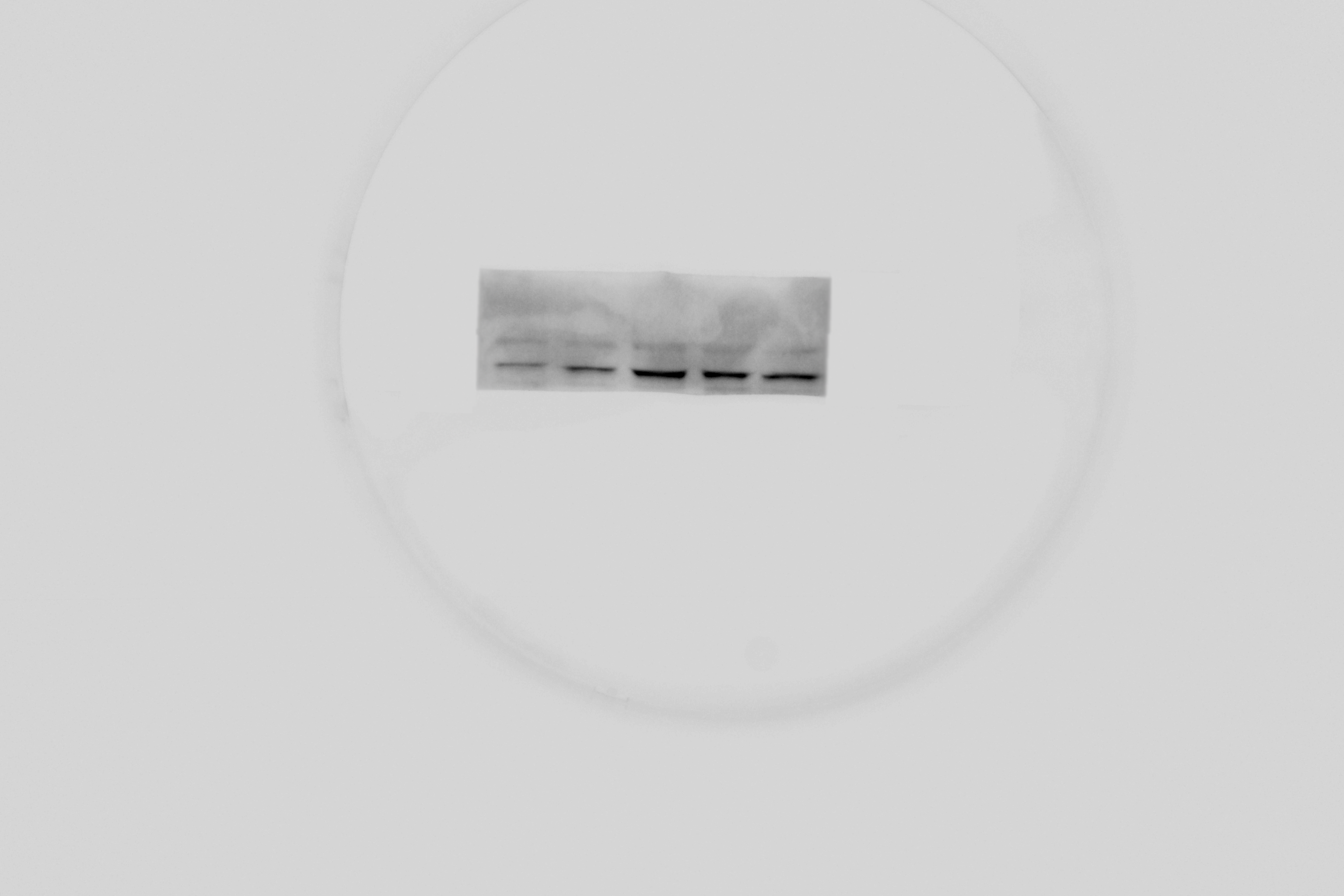

Supplement: S47 Fig — (TIF) [file pone.0153919.s047.tif]

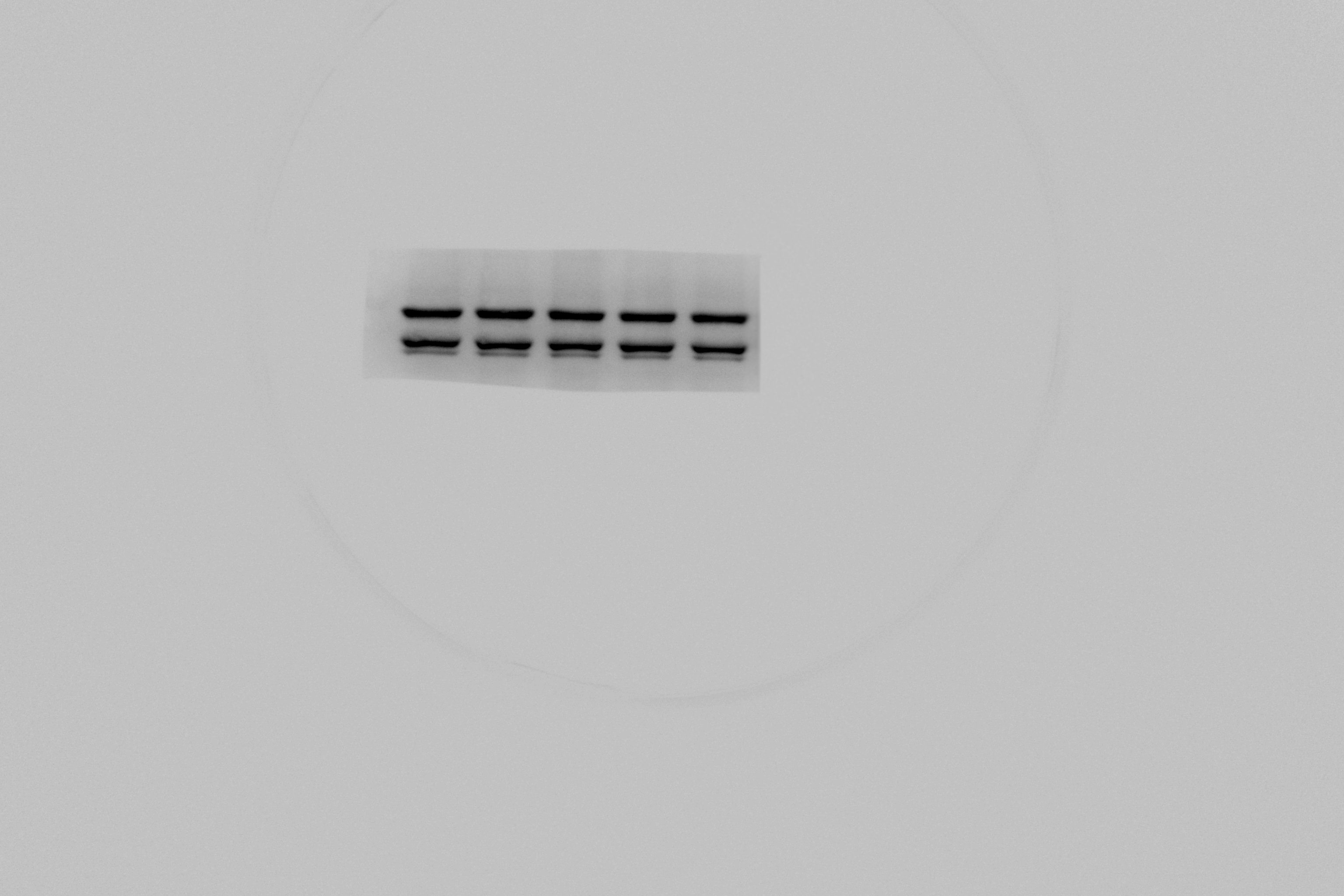

Supplement: S48 Fig — (TIF) [file pone.0153919.s048.tif]

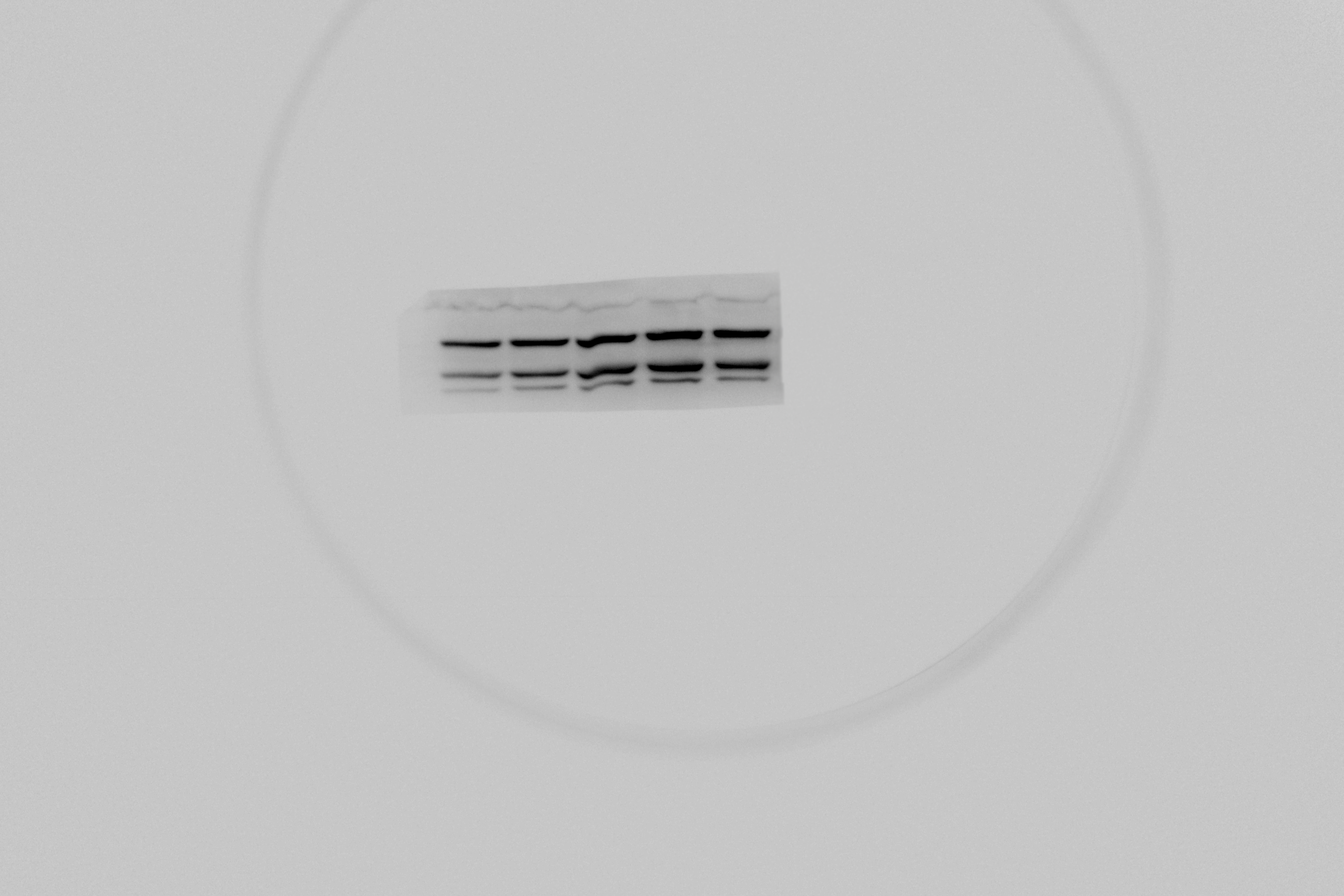

Supplement: S49 Fig — (TIF) [file pone.0153919.s049.tif]

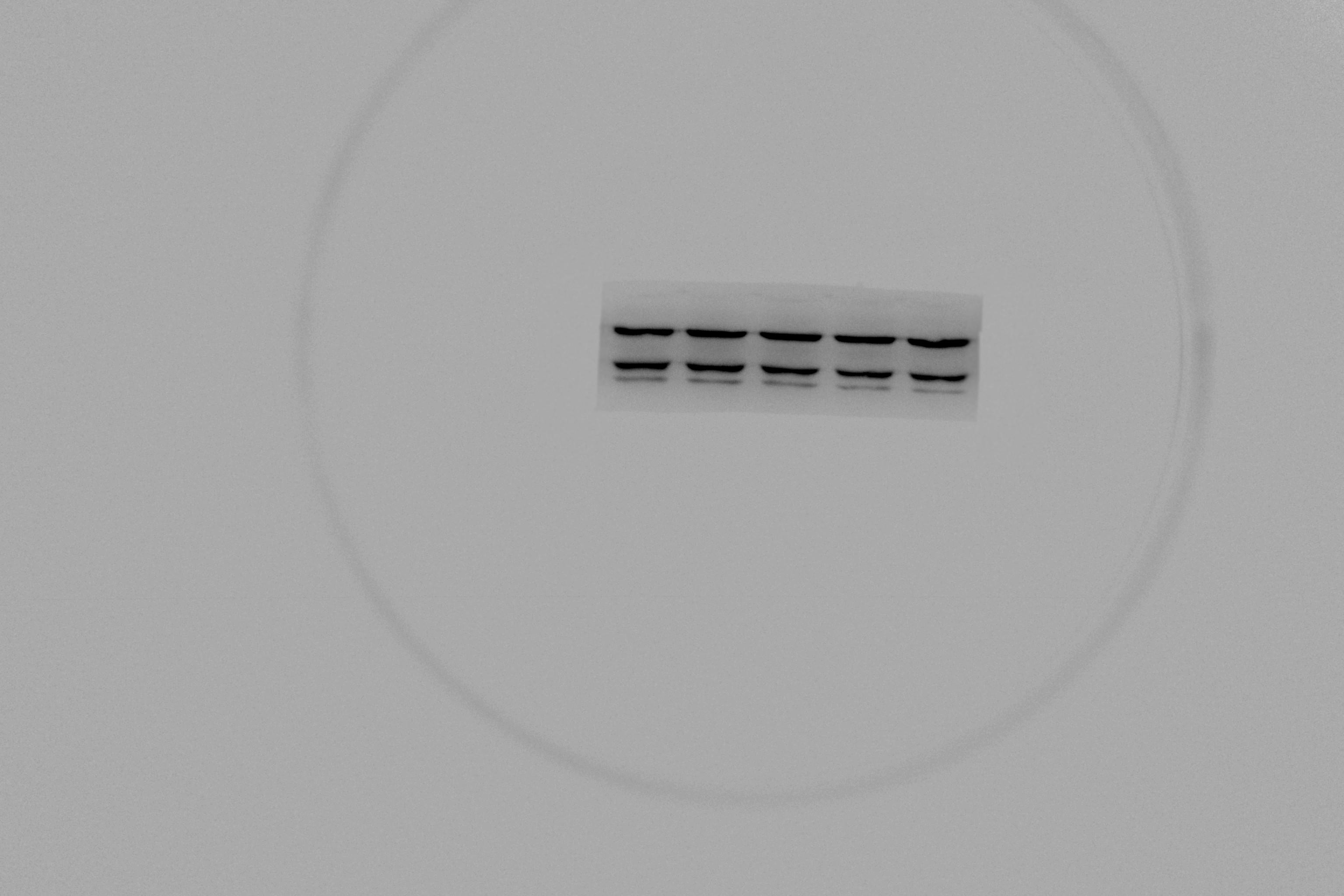

Supplement: S50 Fig — (TIF) [file pone.0153919.s050.tif]

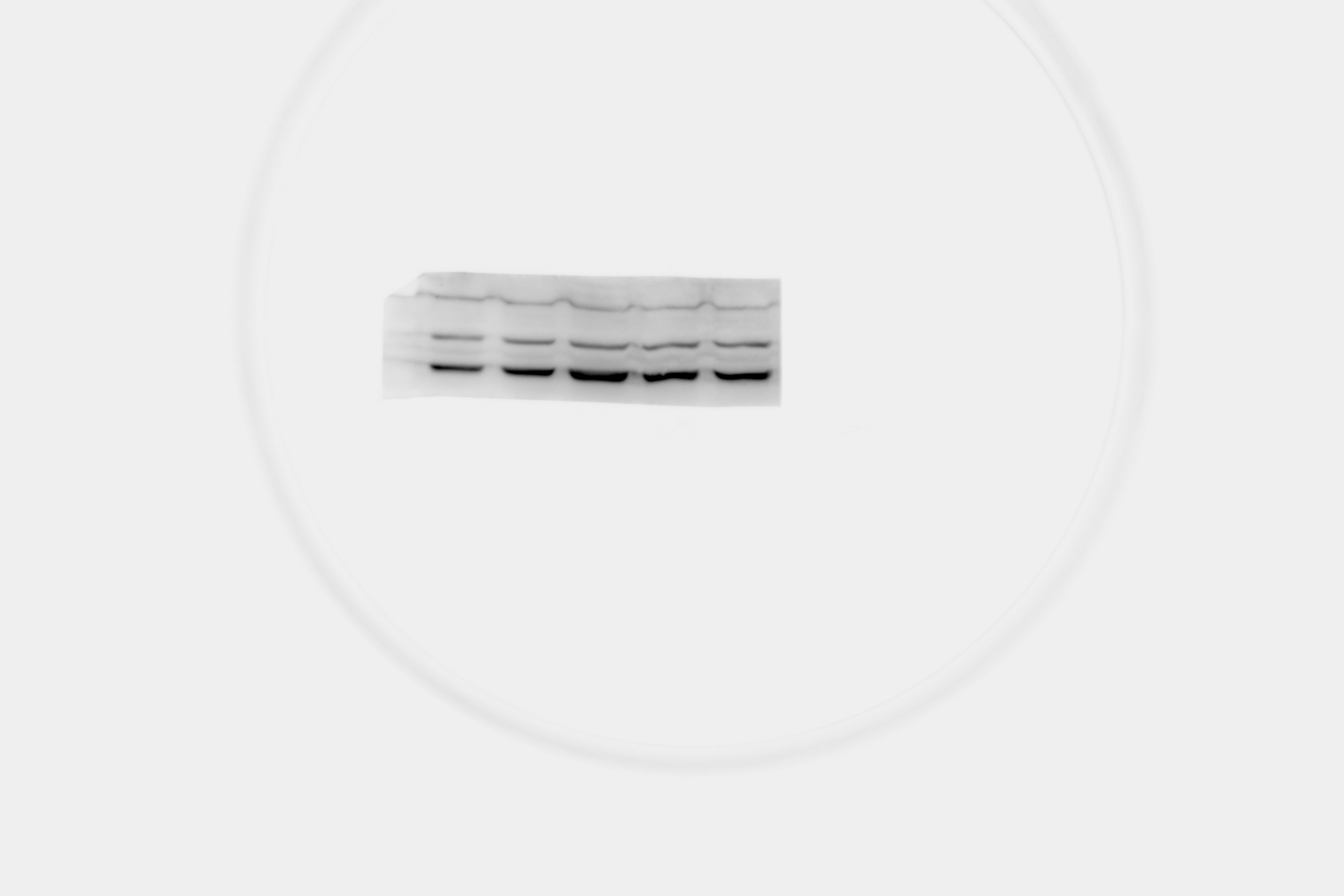

Supplement: S51 Fig — (TIF) [file pone.0153919.s051.tif]

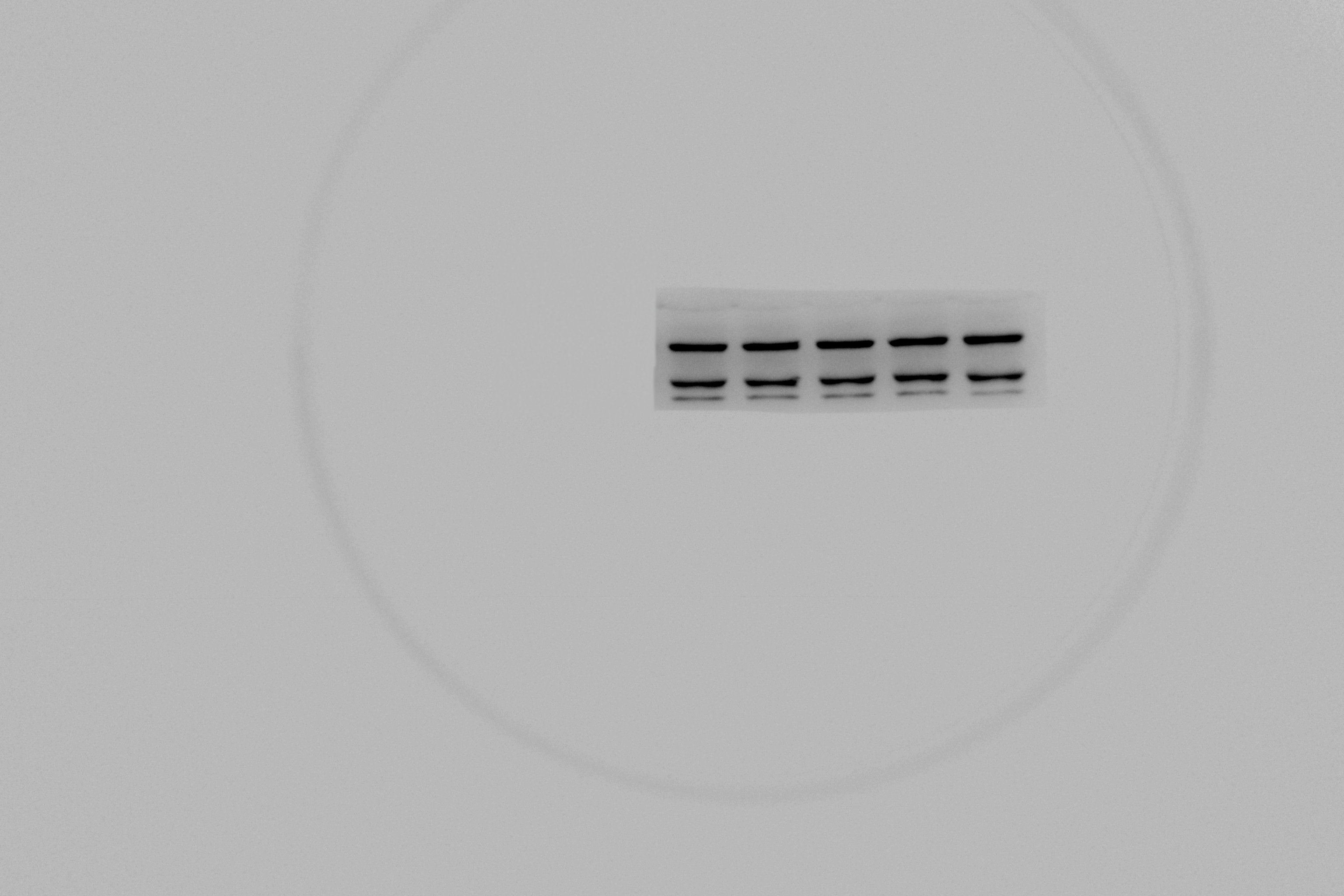

Supplement: S52 Fig — (TIF) [file pone.0153919.s052.tif]

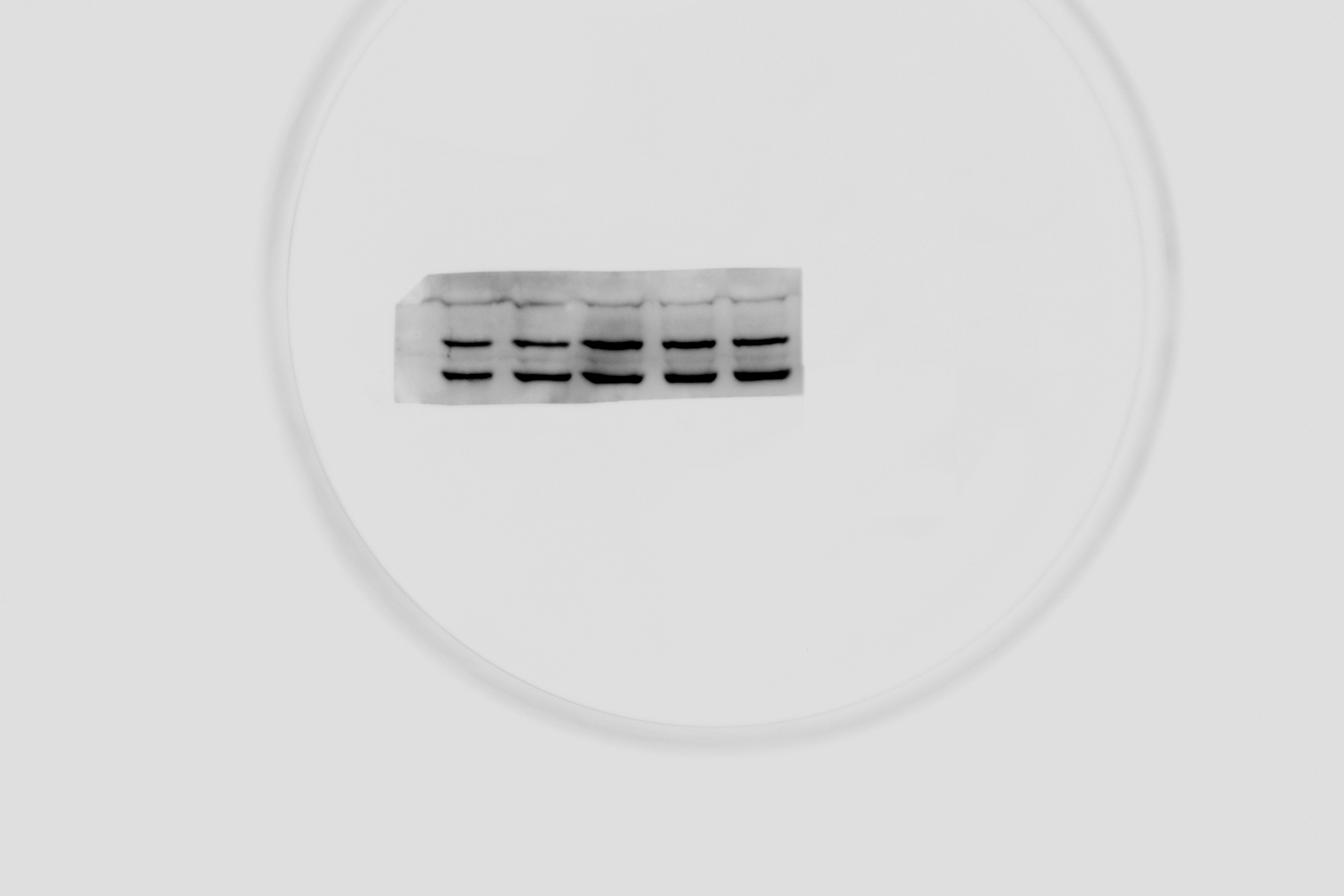

Supplement: S53 Fig — (TIF) [file pone.0153919.s053.tif]

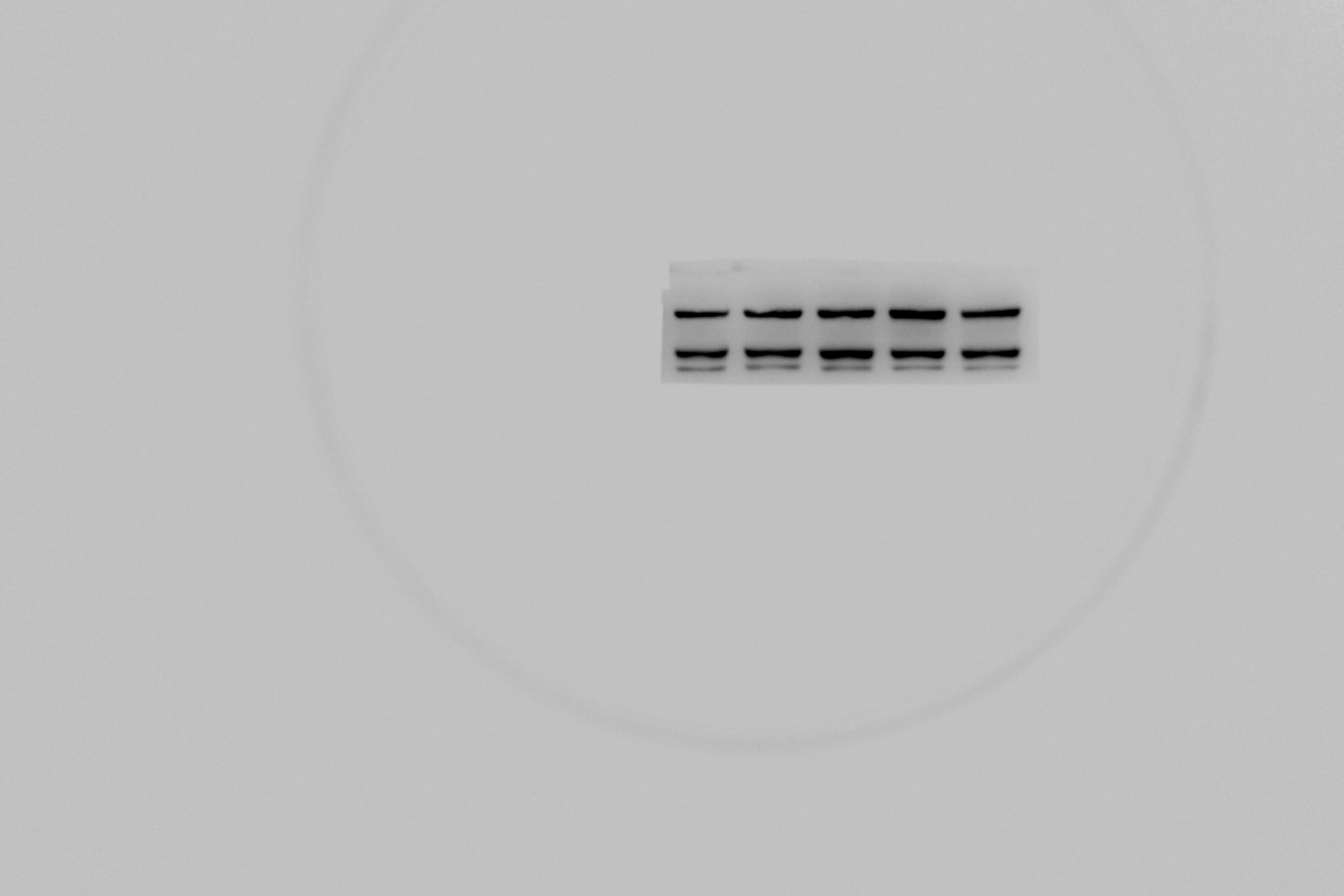

Supplement: S54 Fig — (TIF) [file pone.0153919.s054.tif]

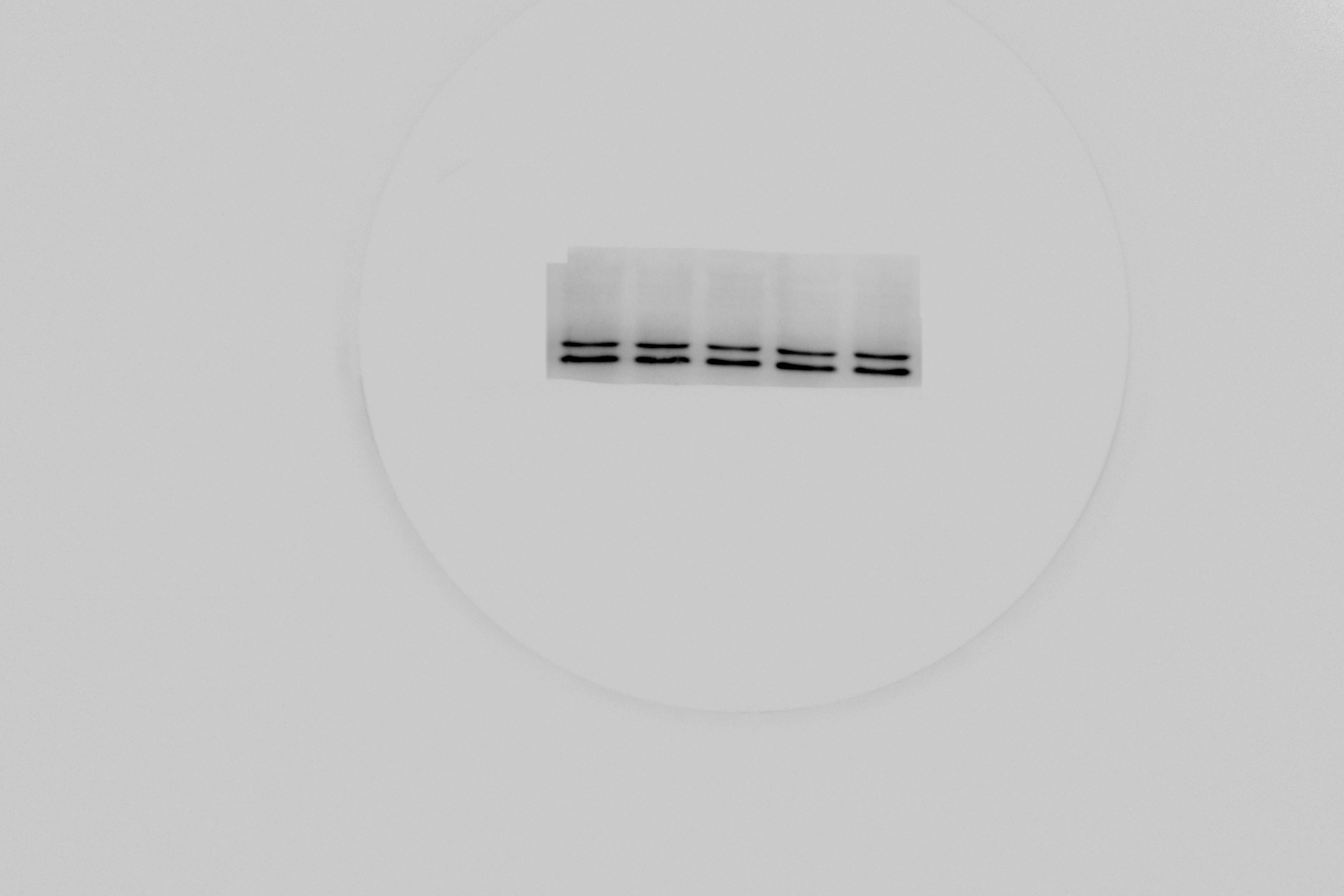

Supplement: S55 Fig — (TIF) [file pone.0153919.s055.tif]

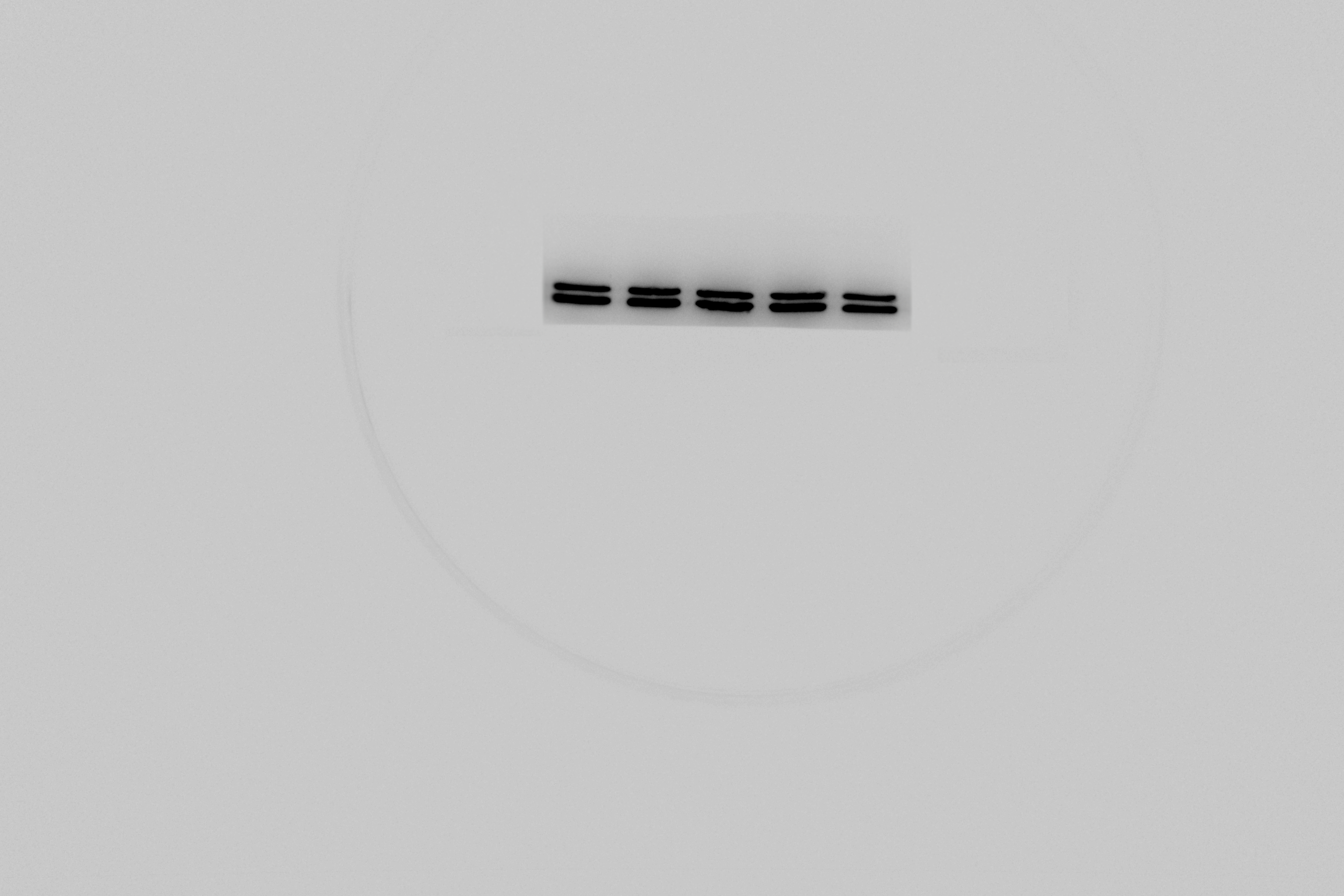

Supplement: S56 Fig — (TIF) [file pone.0153919.s056.tif]

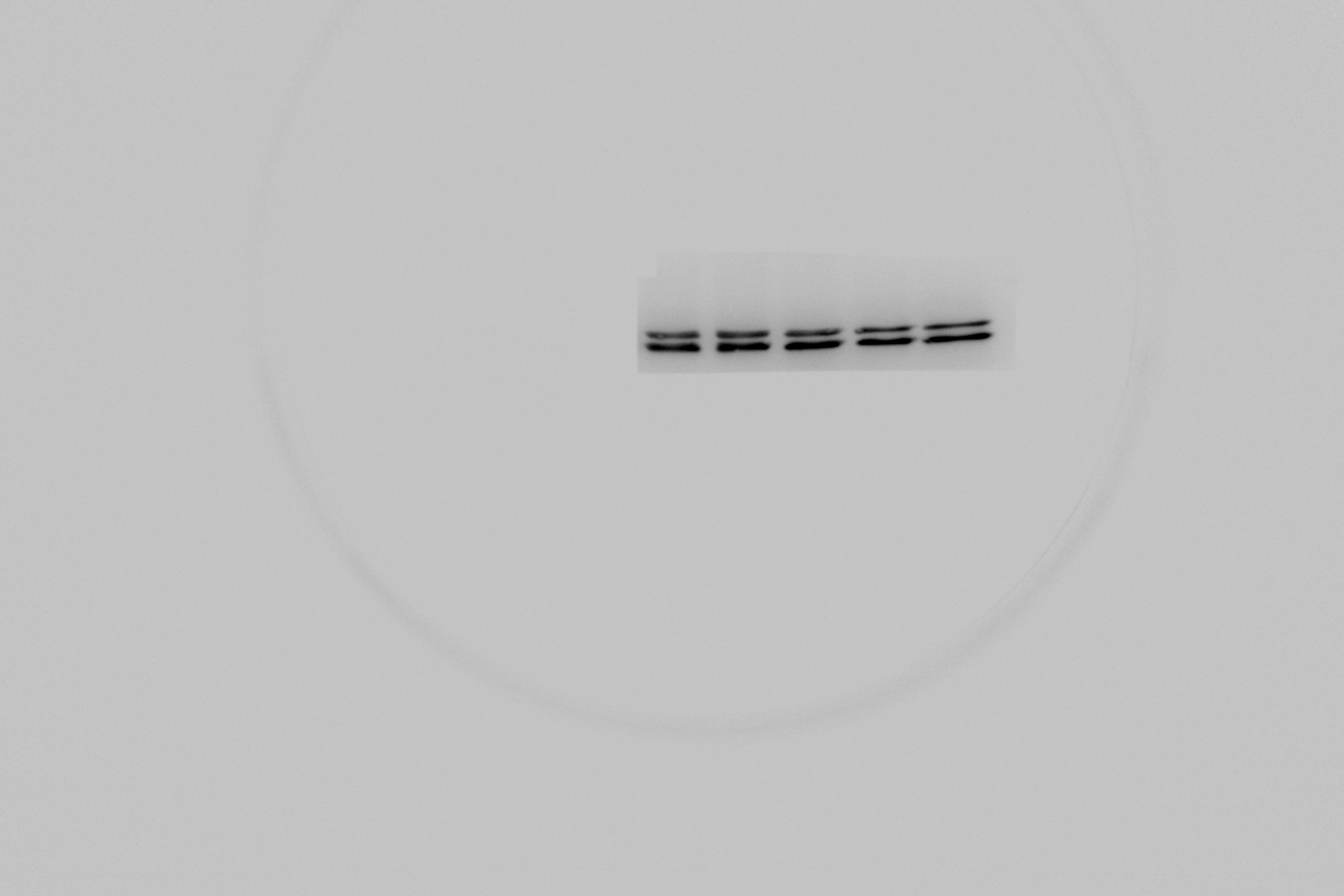

Supplement: S57 Fig — (TIF) [file pone.0153919.s057.tif]

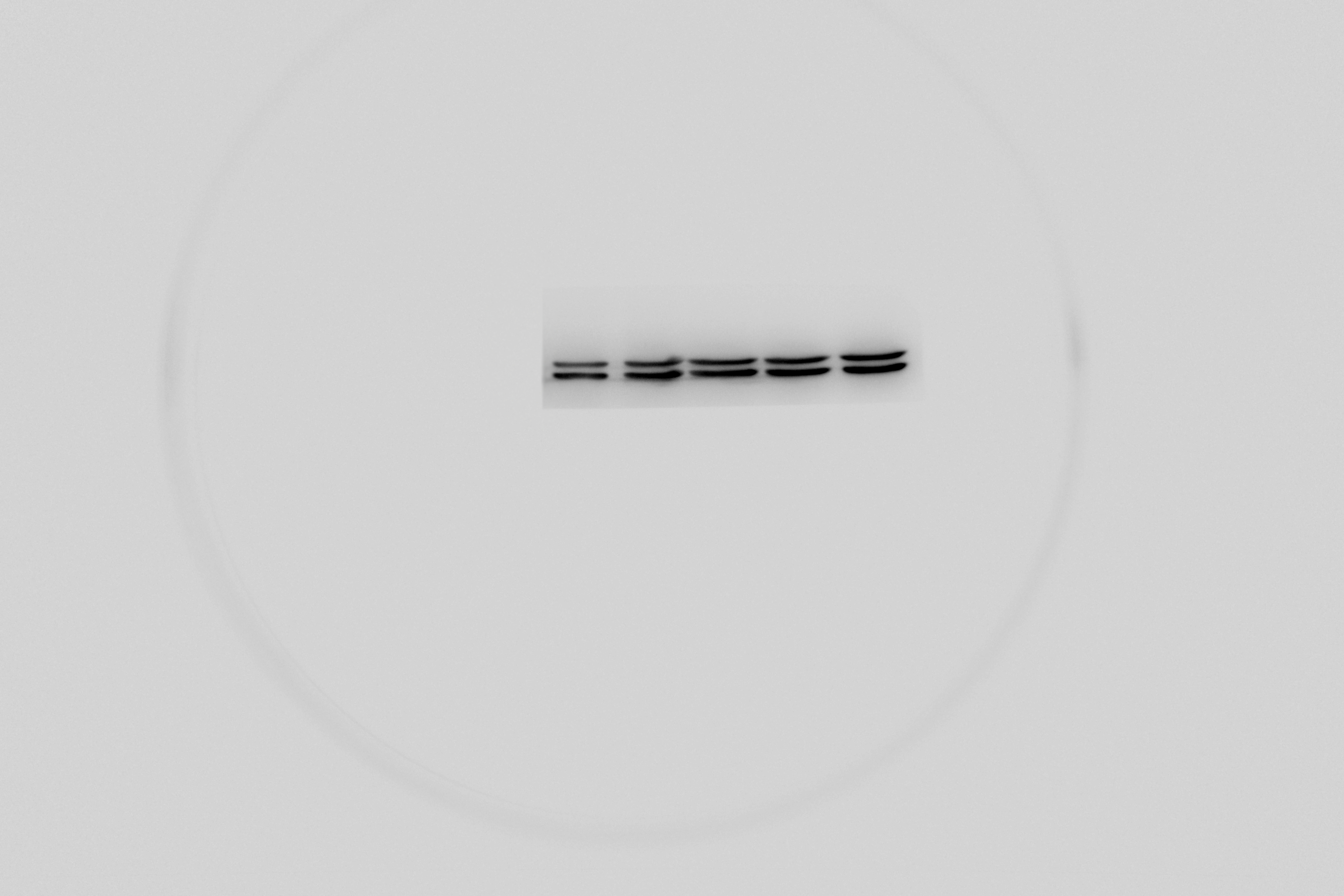

Supplement: S58 Fig — (TIF) [file pone.0153919.s058.tif]

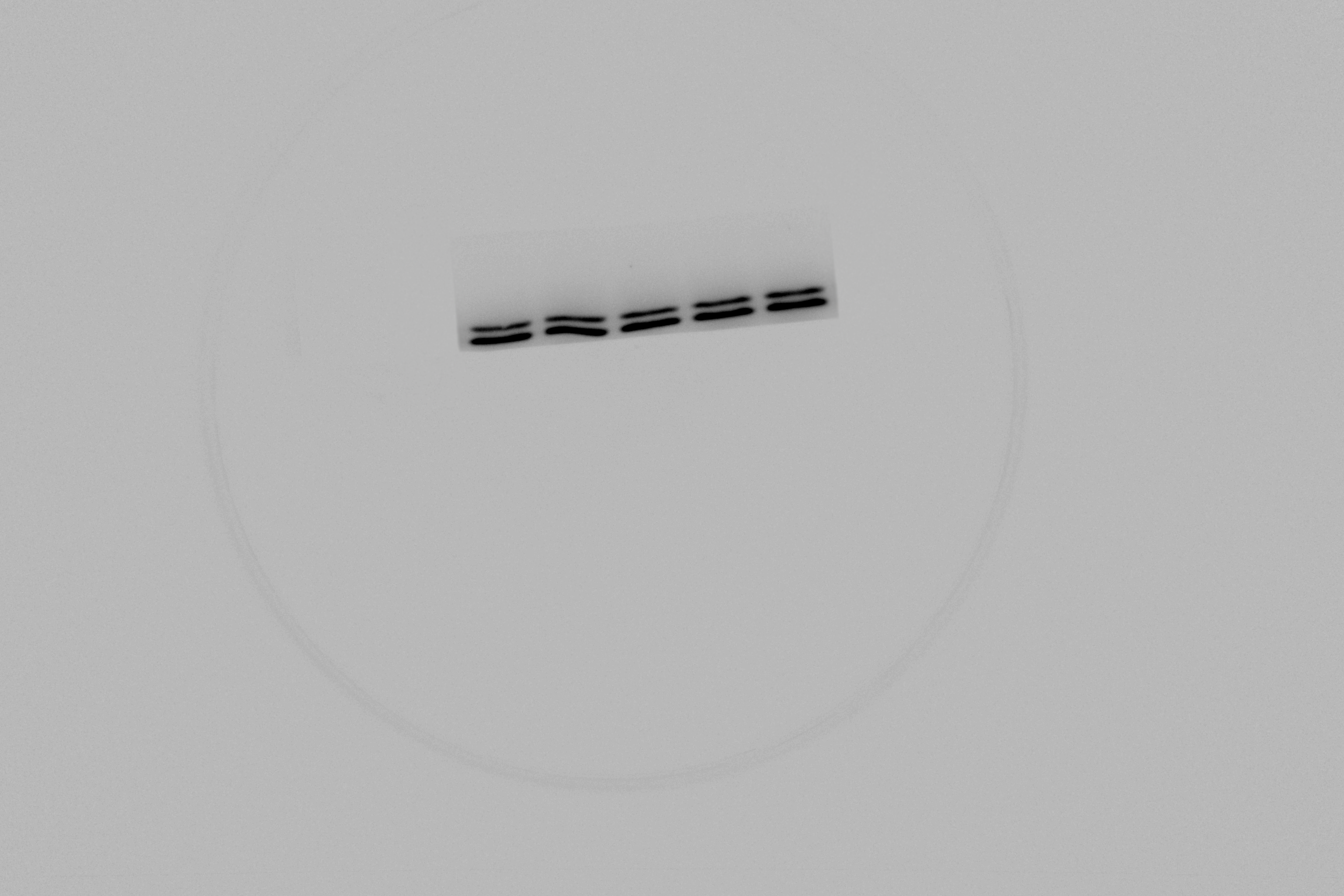

Supplement: S59 Fig — (TIF) [file pone.0153919.s059.tif]

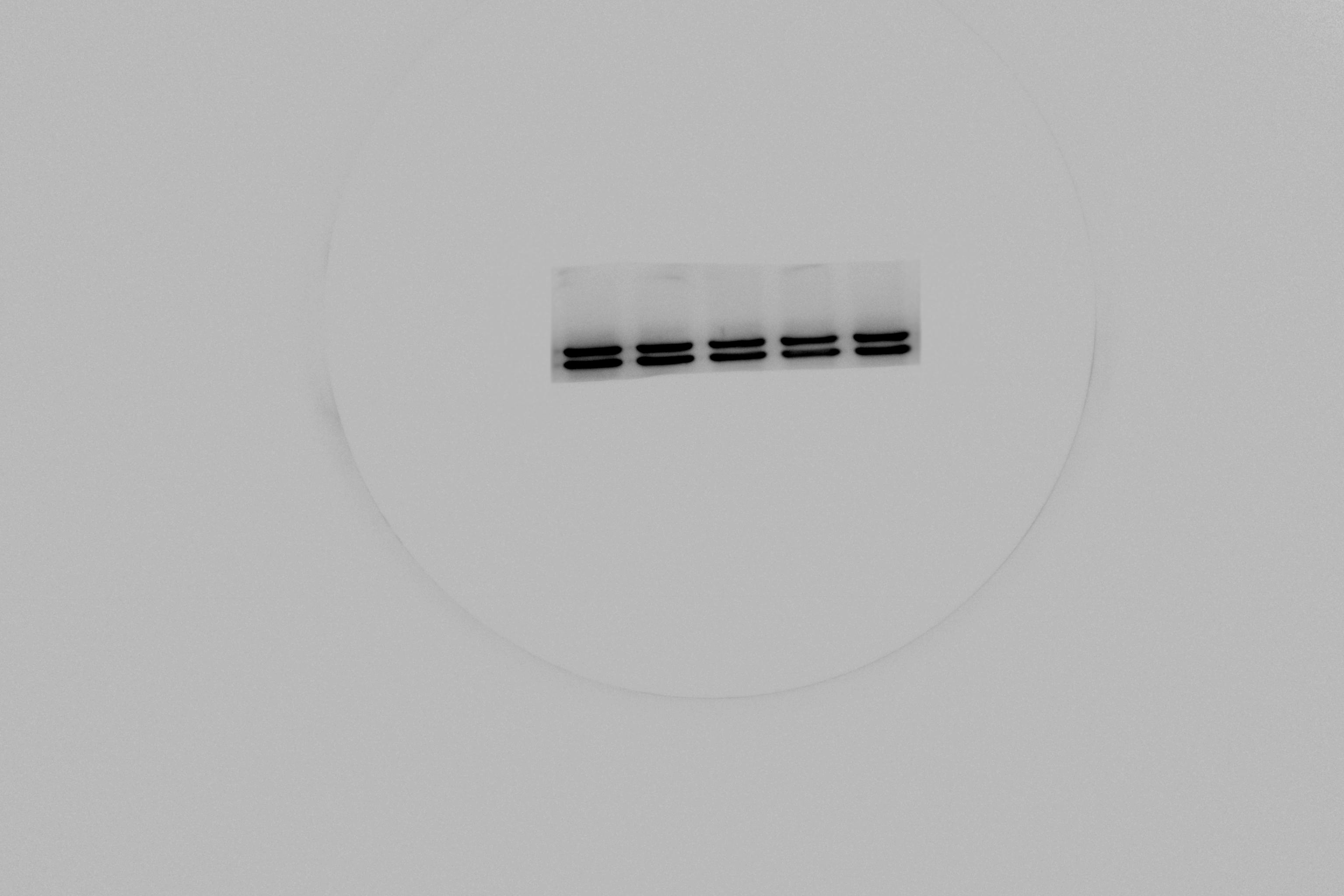

Supplement: S60 Fig — (TIF) [file pone.0153919.s060.tif]

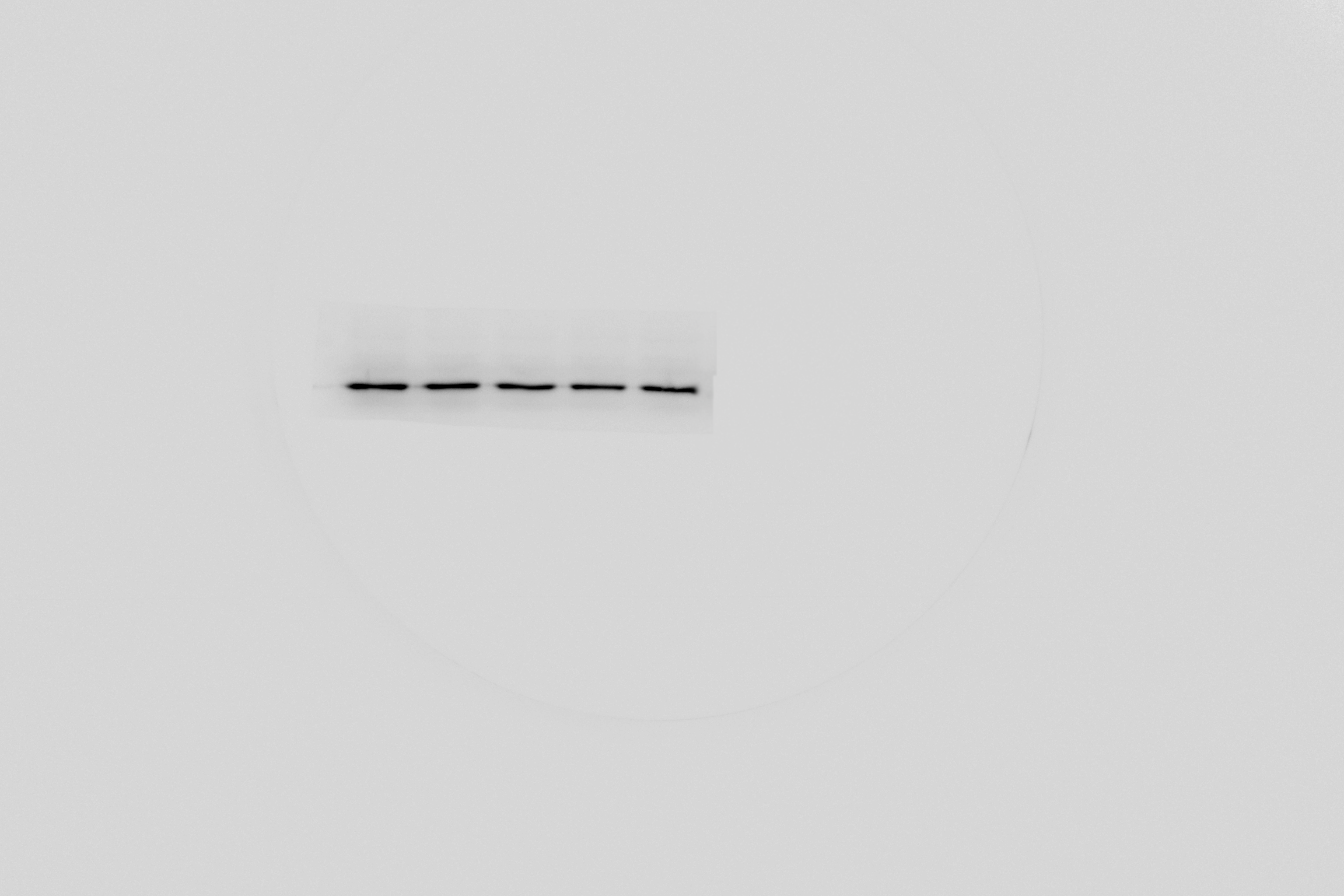

Supplement: S61 Fig — (TIF) [file pone.0153919.s061.tif]

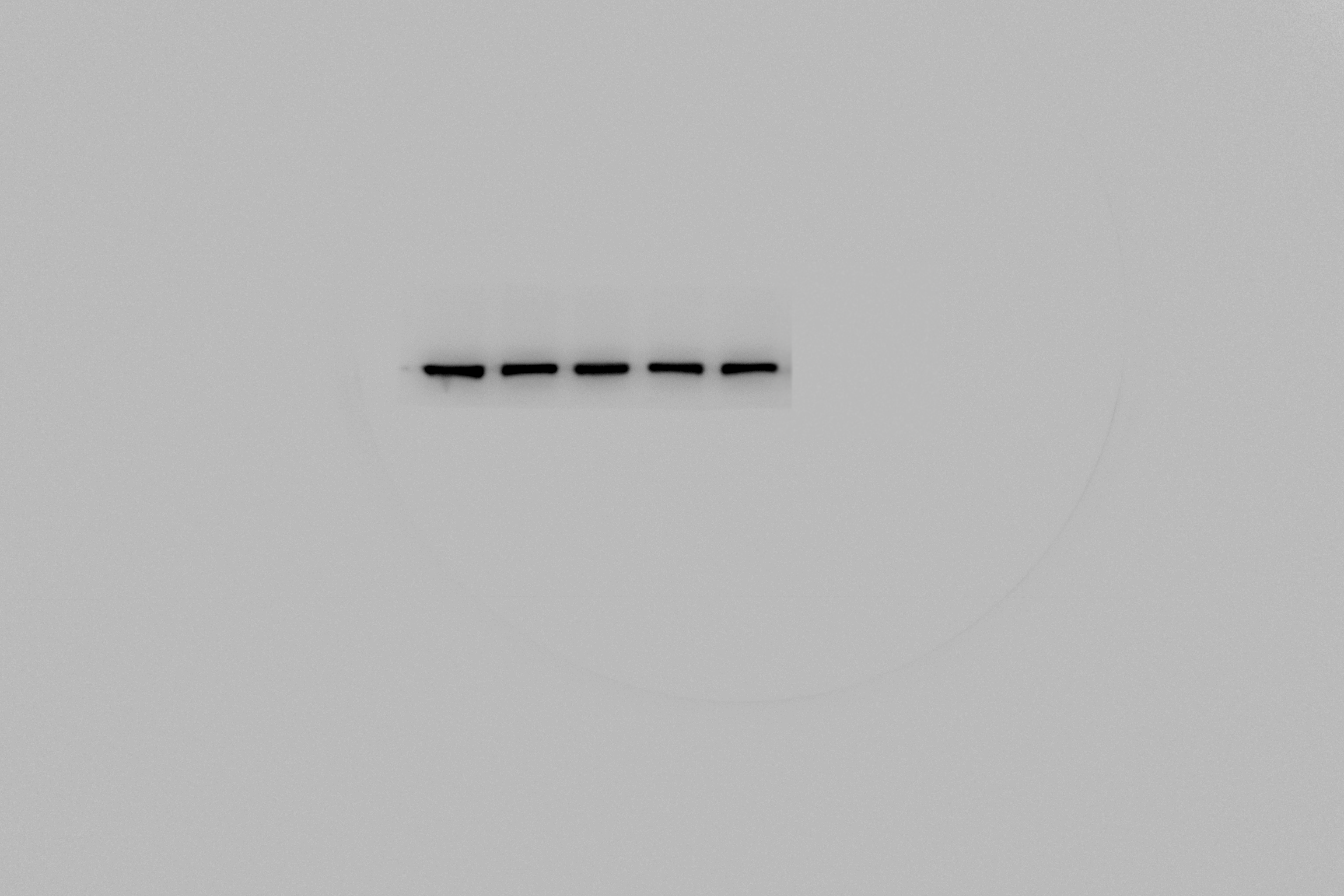

Supplement: S62 Fig — (TIF) [file pone.0153919.s062.tif]

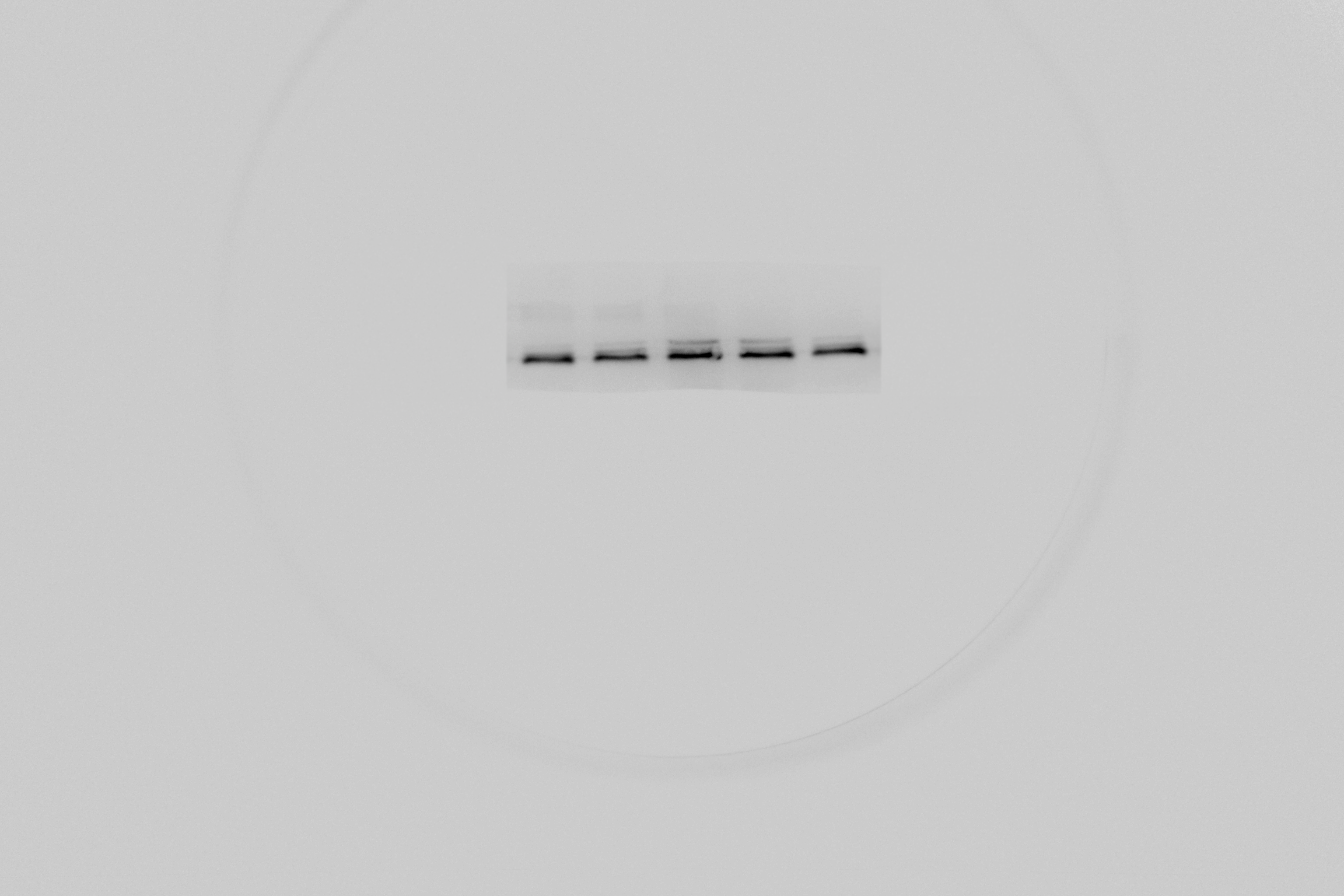

Supplement: S63 Fig — (TIF) [file pone.0153919.s063.tif]

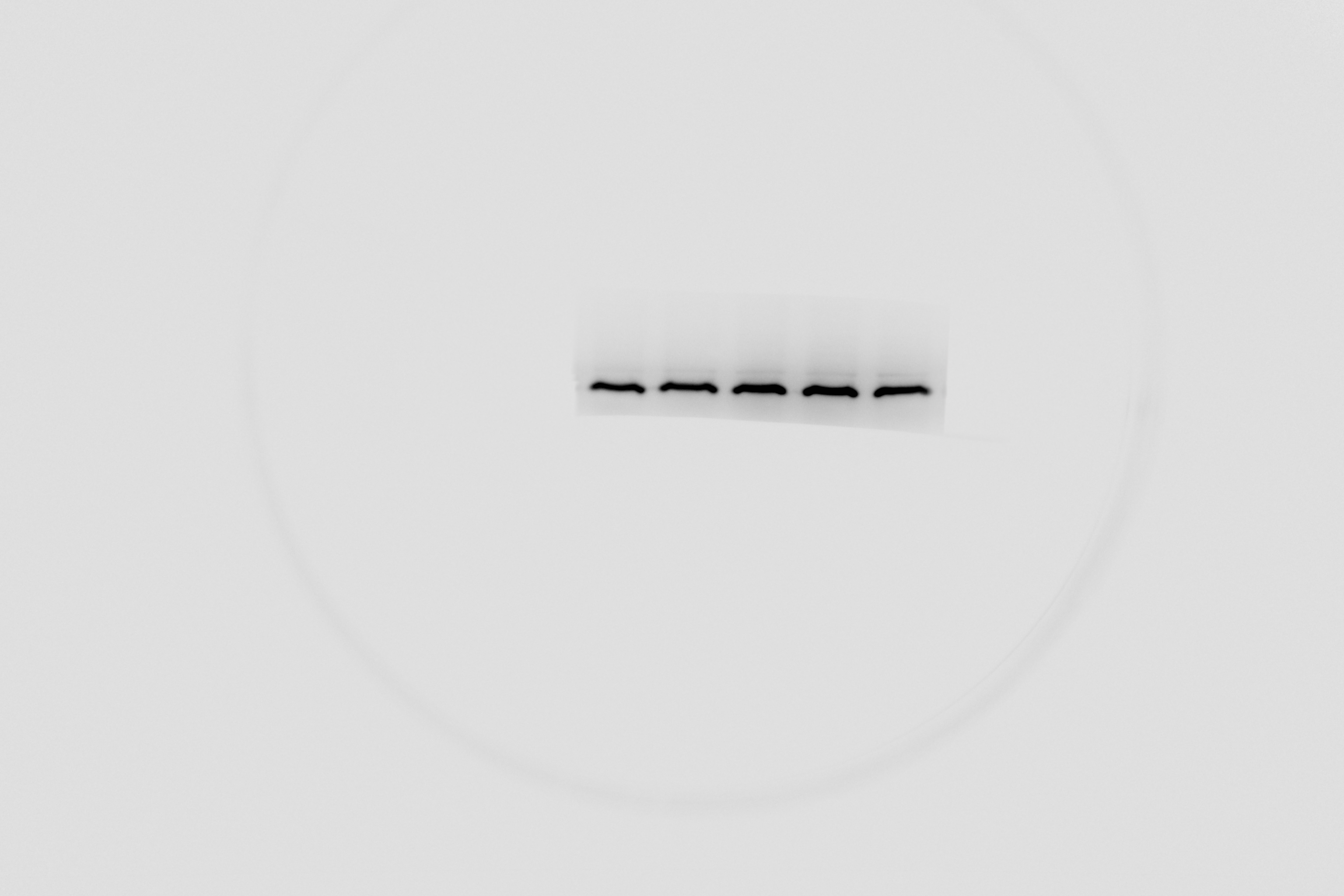

Supplement: S64 Fig — (TIF) [file pone.0153919.s064.tif]

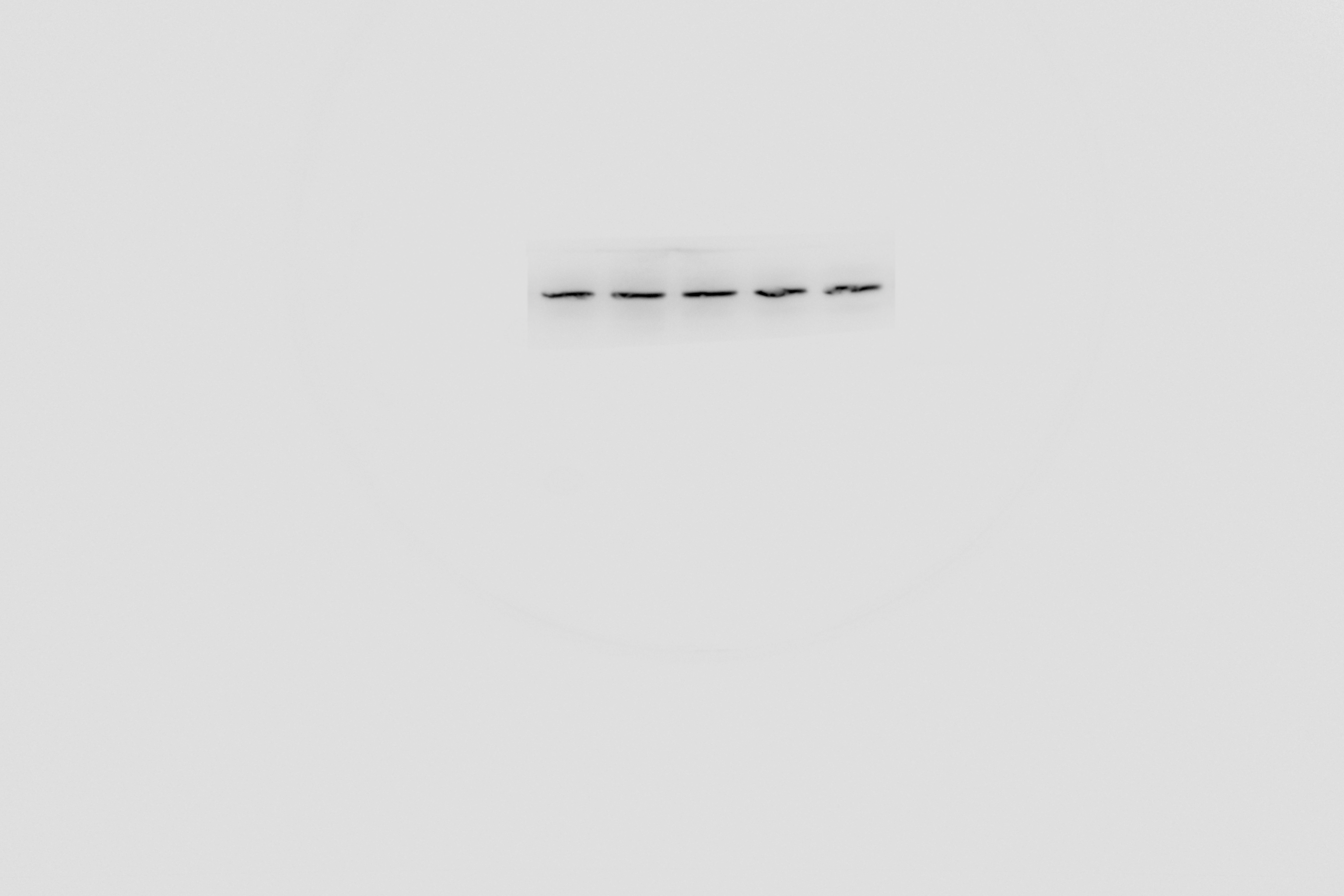

Supplement: S65 Fig — (TIF) [file pone.0153919.s065.tif]

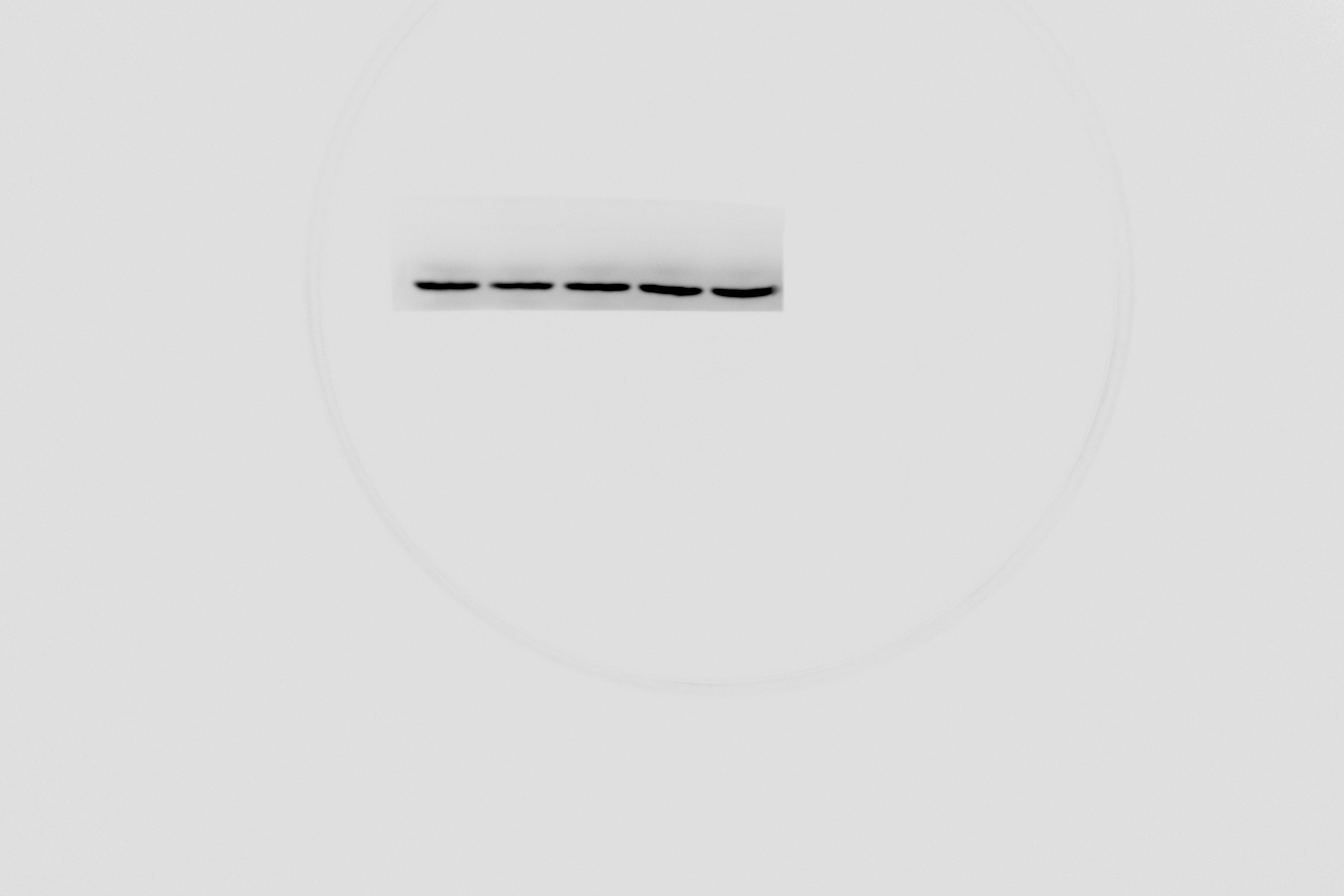

Supplement: S66 Fig — (TIF) [file pone.0153919.s066.tif]

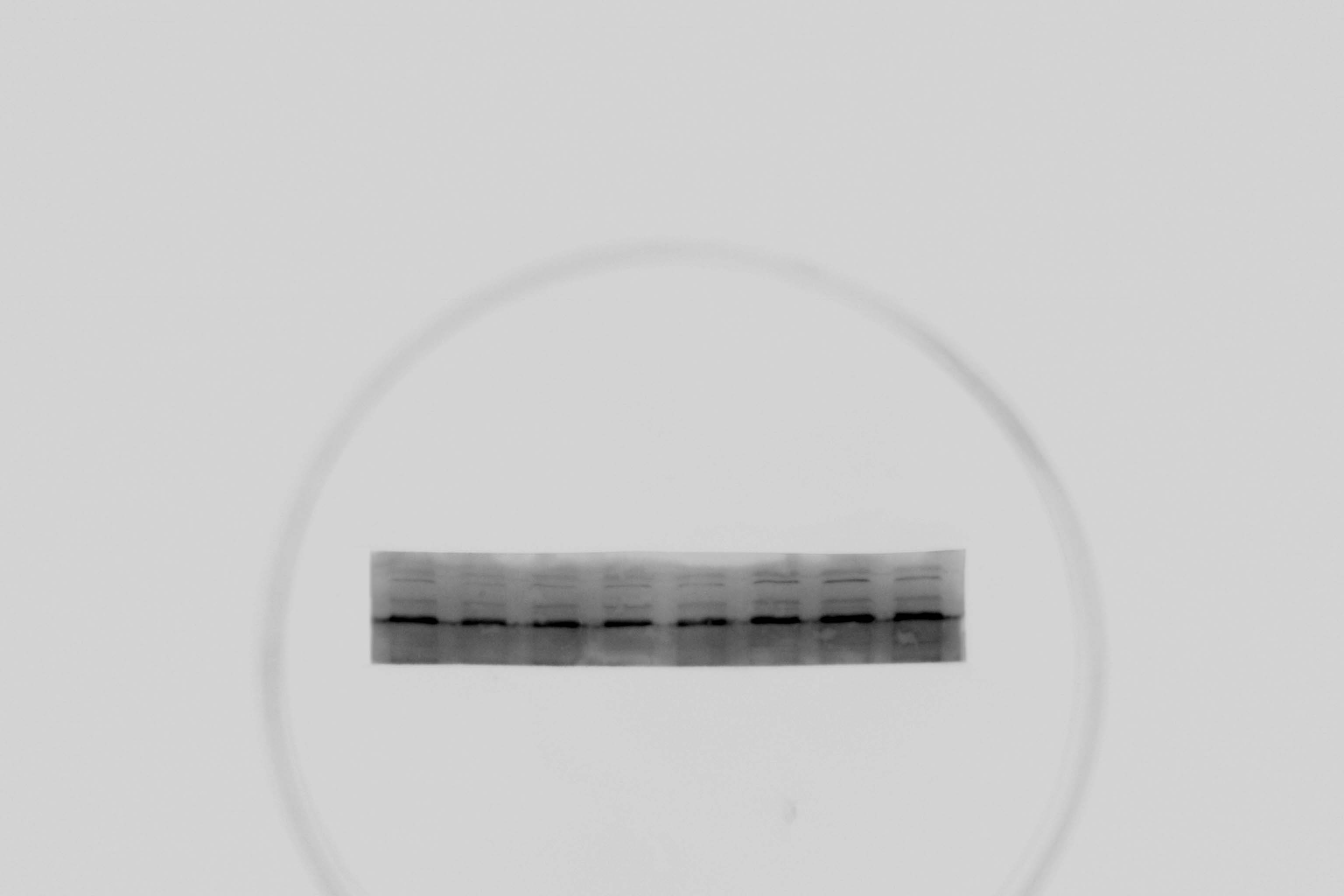

Supplement: S68 Fig — (TIF) [file pone.0153919.s068.tif]

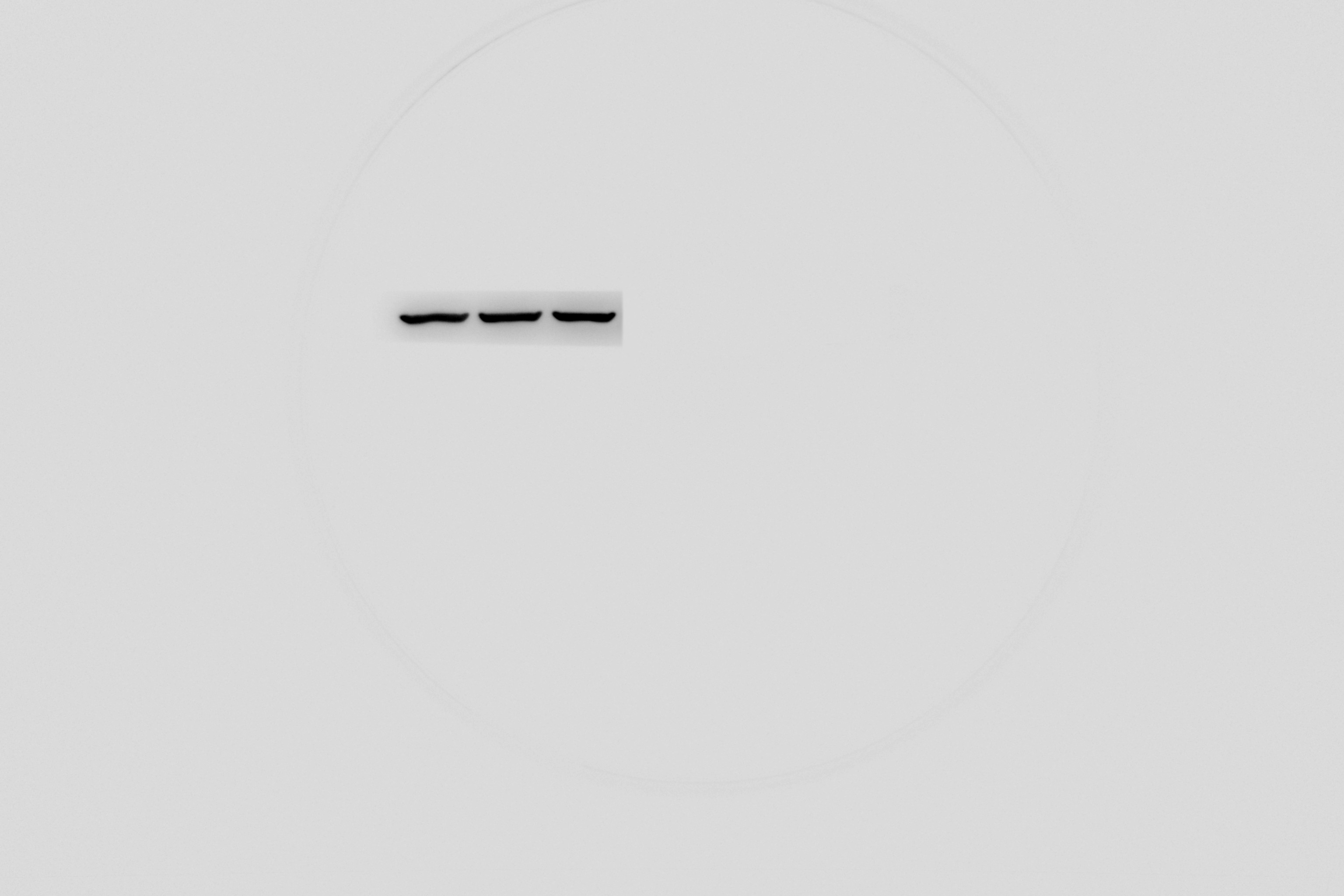

Supplement: S69 Fig — (TIF) [file pone.0153919.s069.tif]

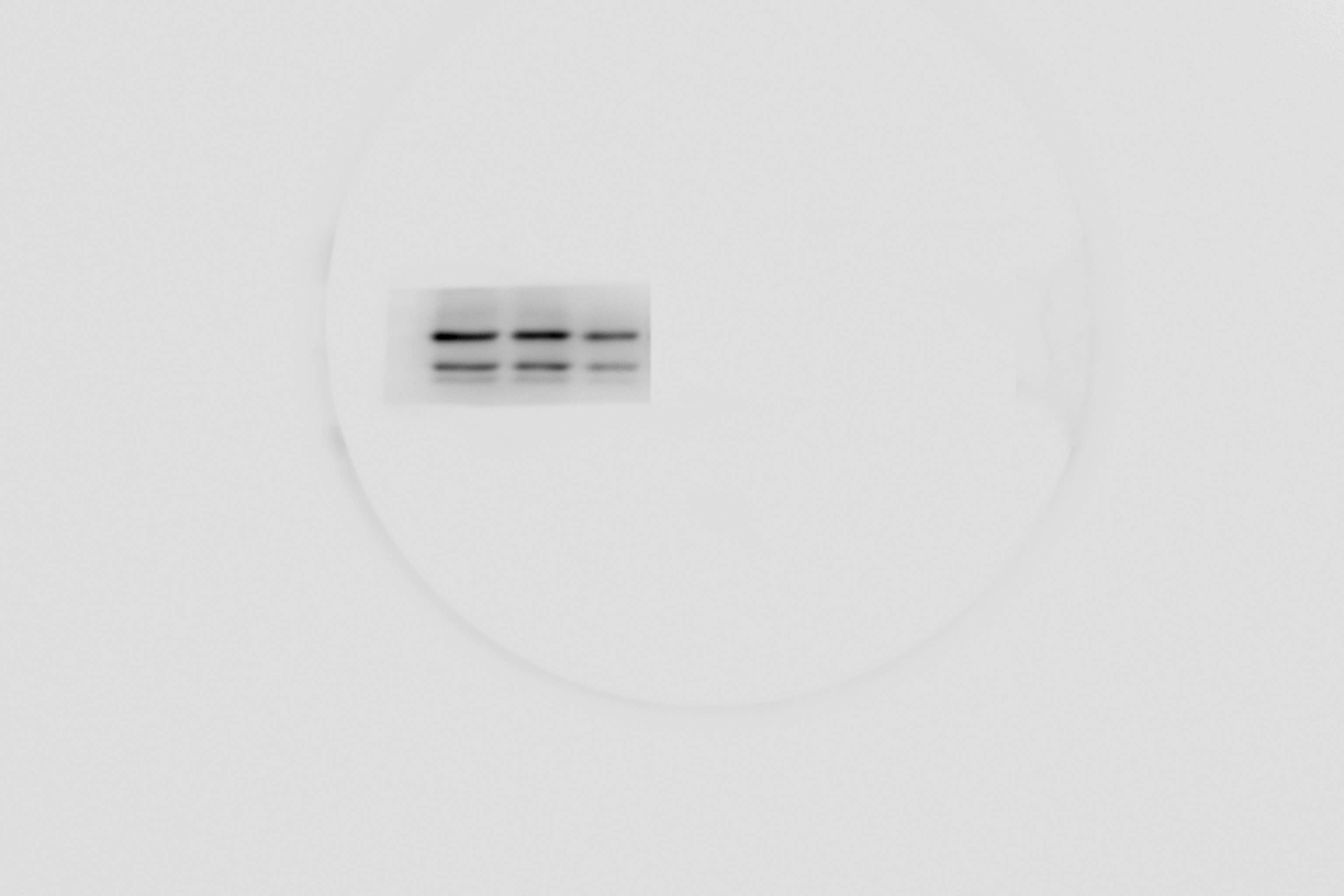

Supplement: S70 Fig — (TIF) [file pone.0153919.s070.tif]

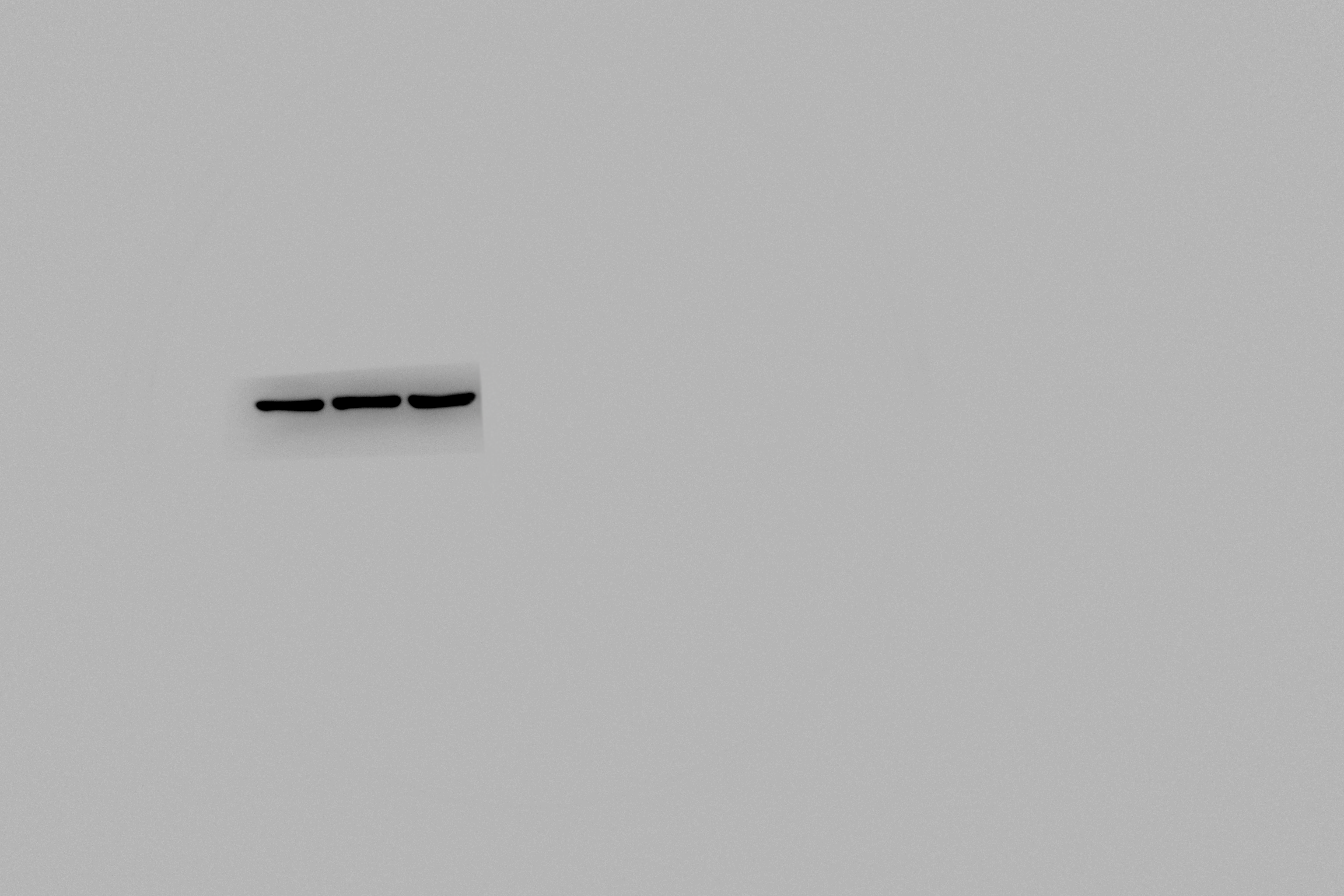

Supplement: S71 Fig — (TIF) [file pone.0153919.s071.tif]

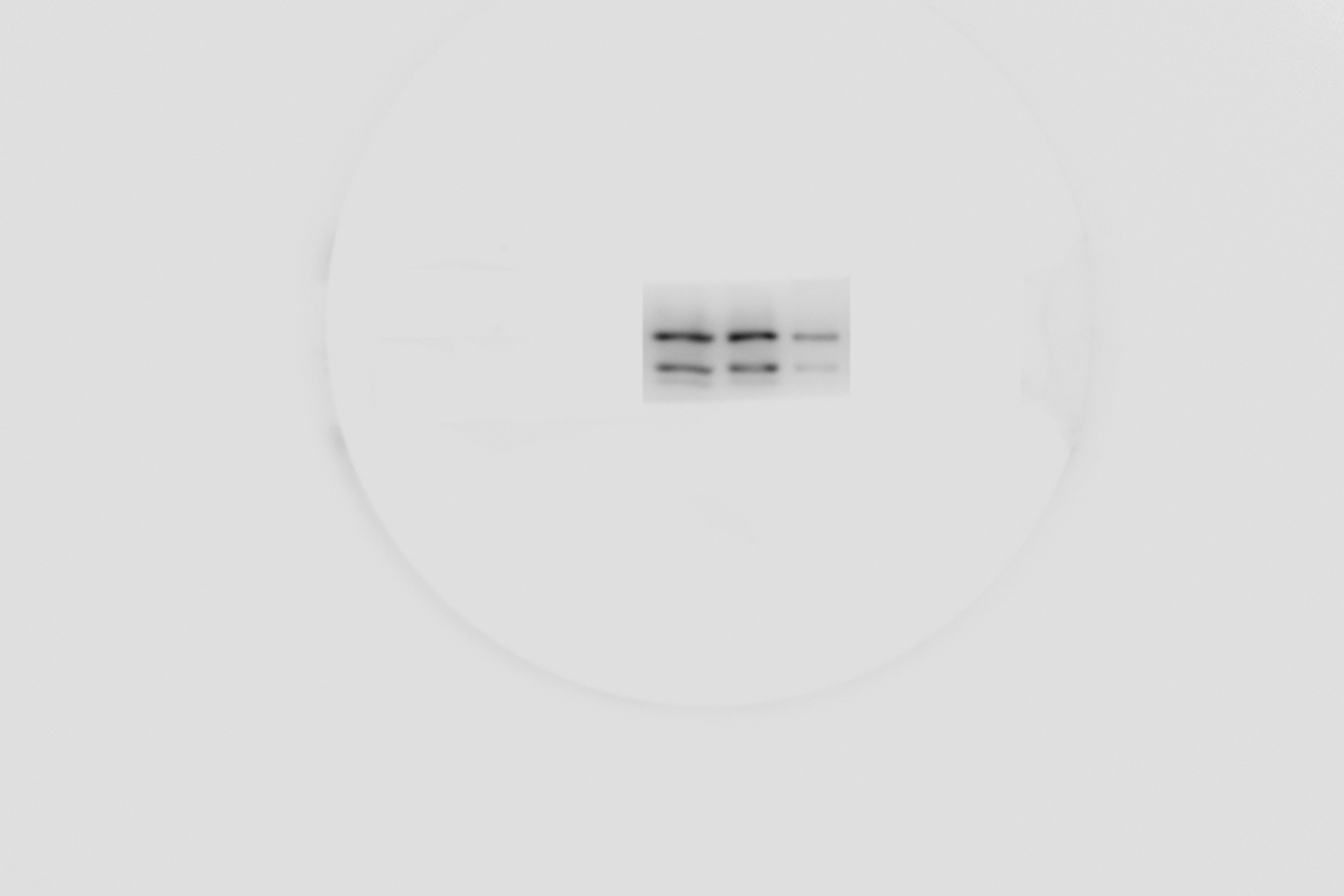

Supplement: S72 Fig — (TIF) [file pone.0153919.s072.tif]

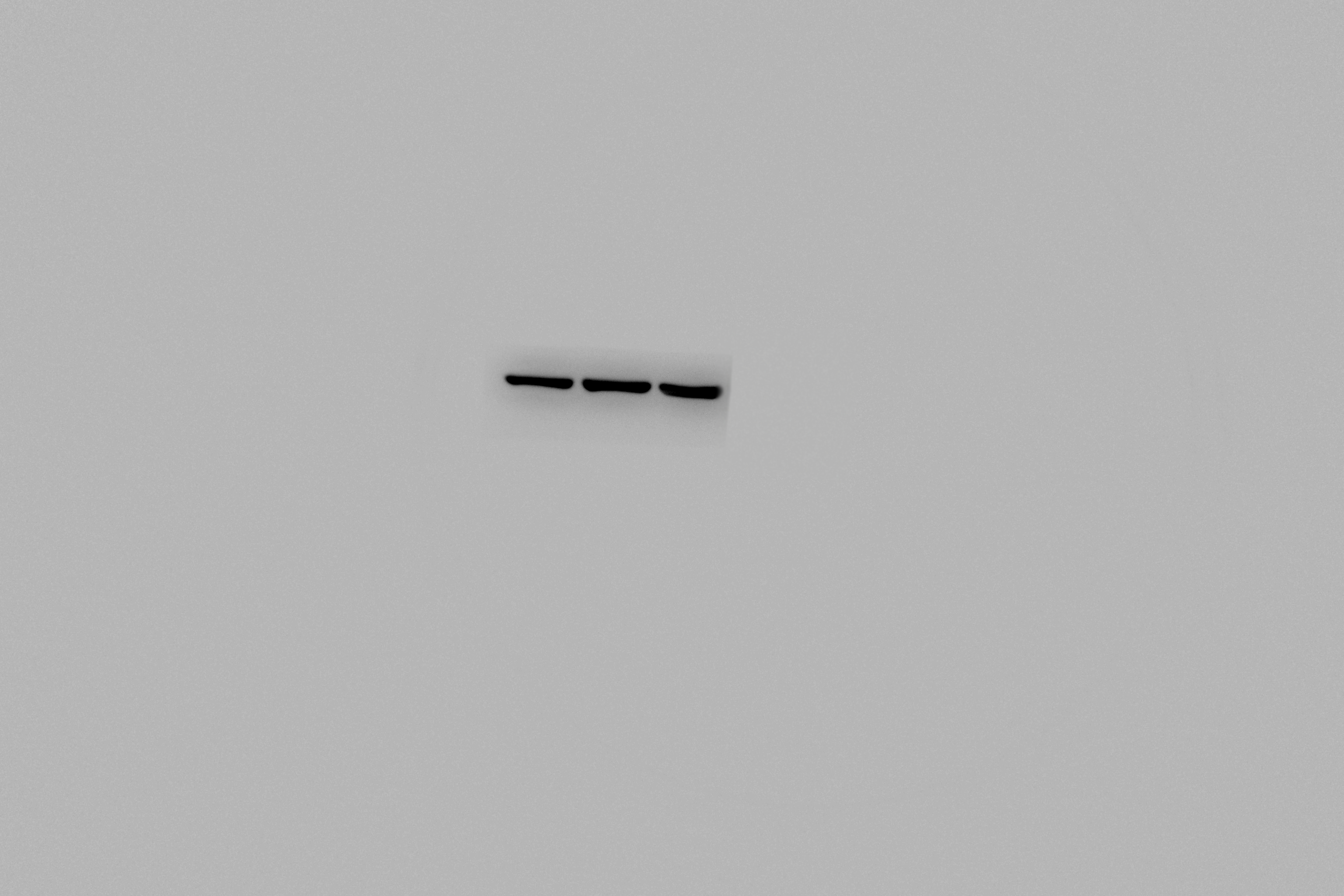

Supplement: S73 Fig — (TIF) [file pone.0153919.s073.tif]

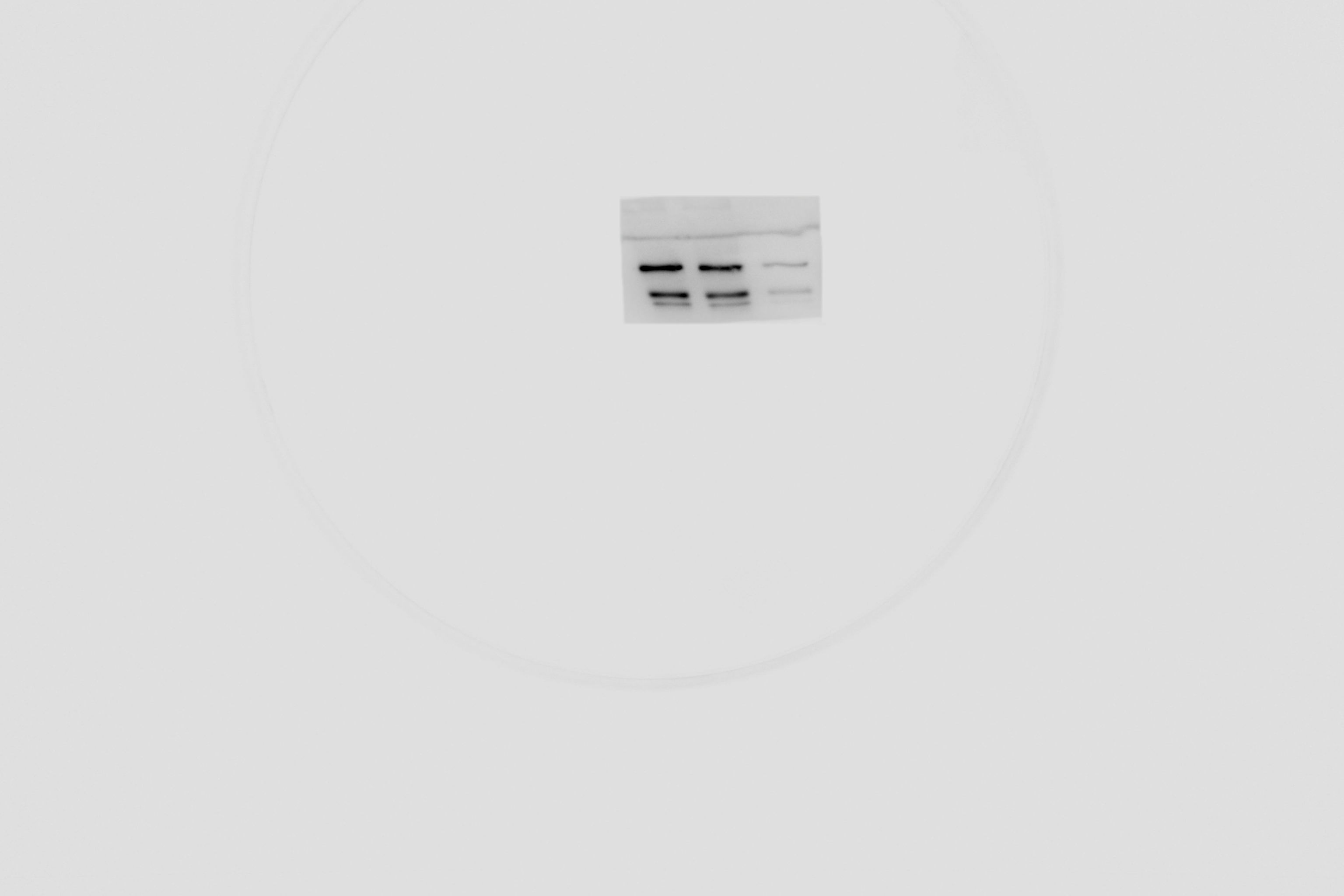

Supplement: S74 Fig — (TIF) [file pone.0153919.s074.tif]

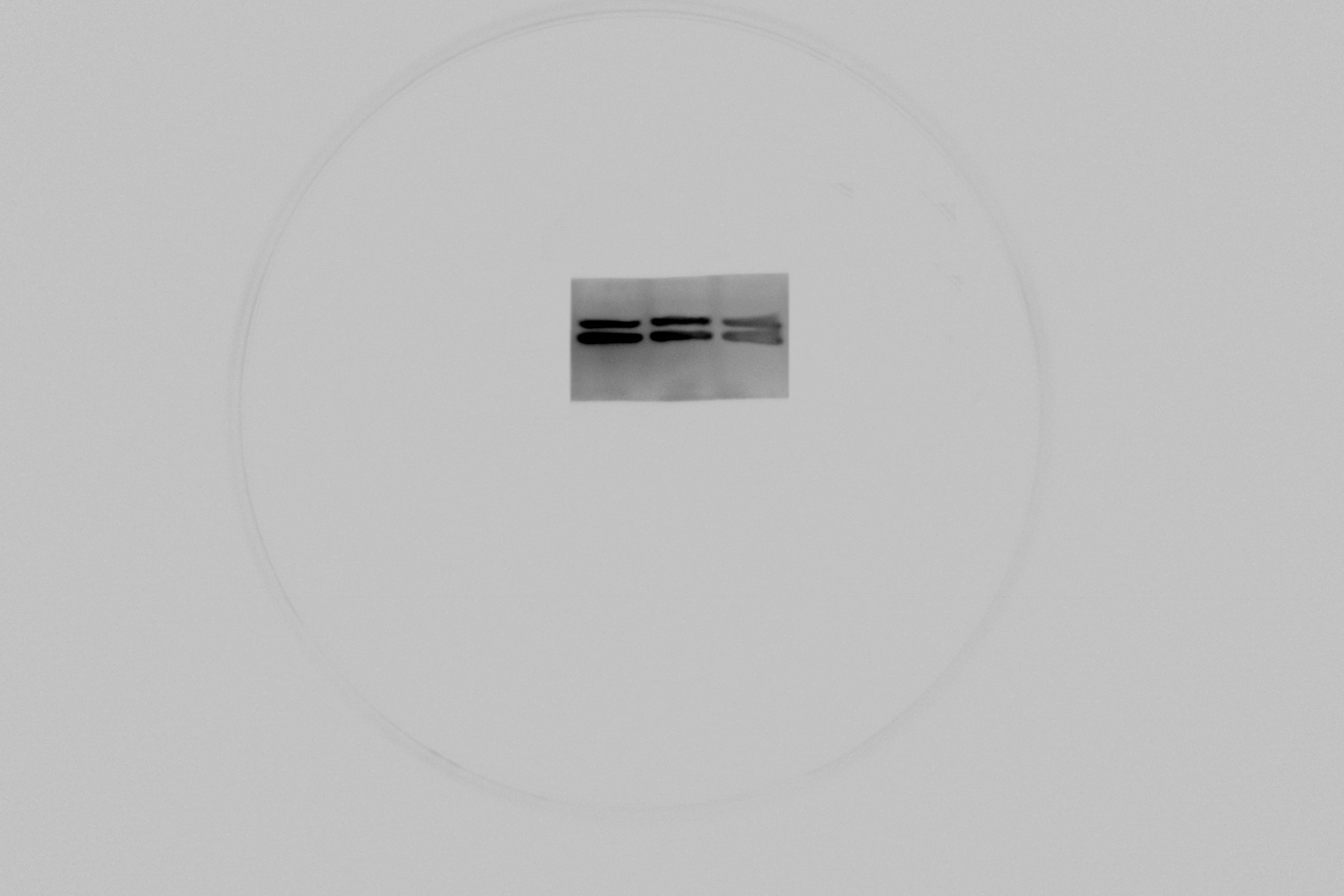

Supplement: S76 Fig — (TIF) [file pone.0153919.s076.tif]

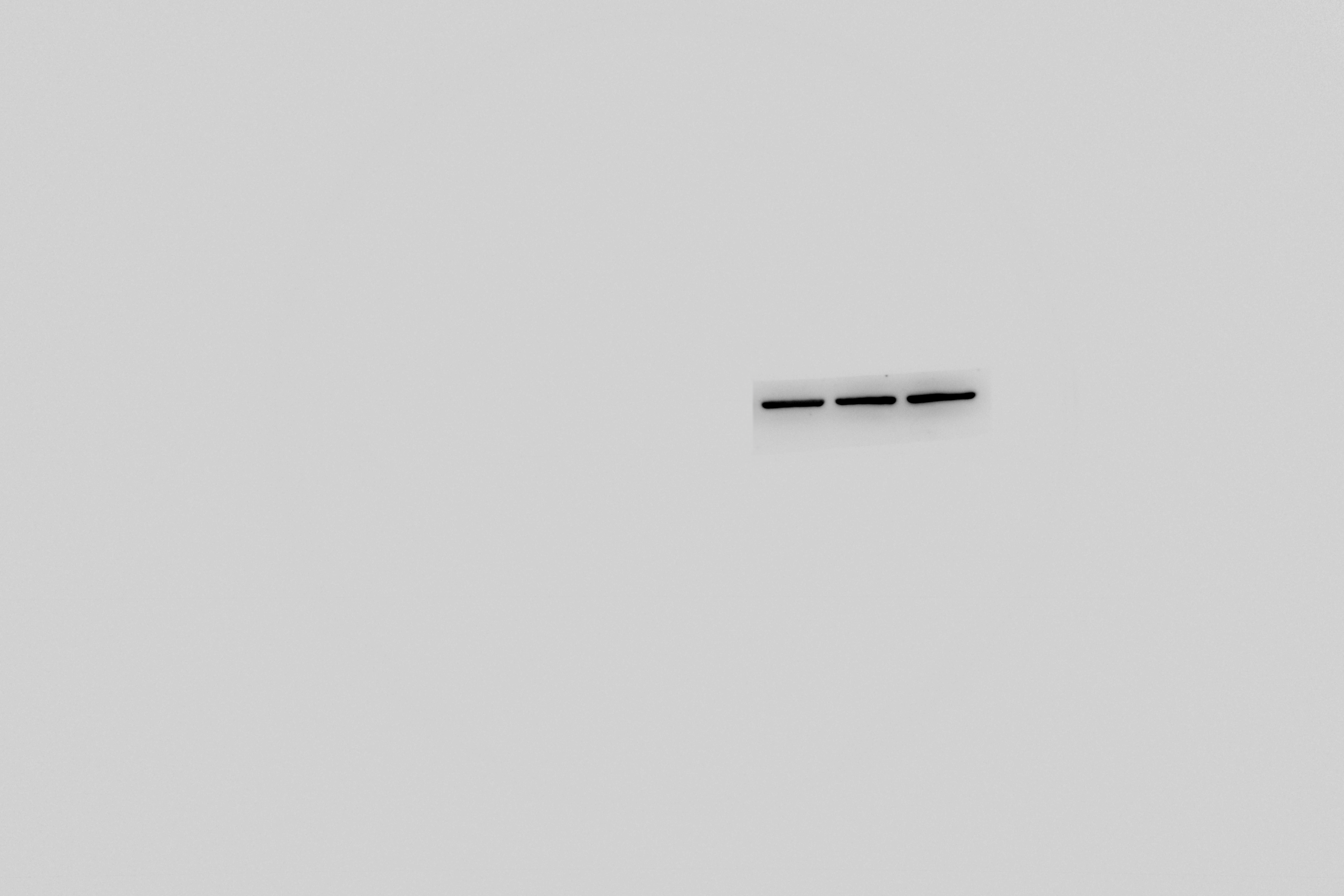

Supplement: S77 Fig — (TIF) [file pone.0153919.s077.tif]

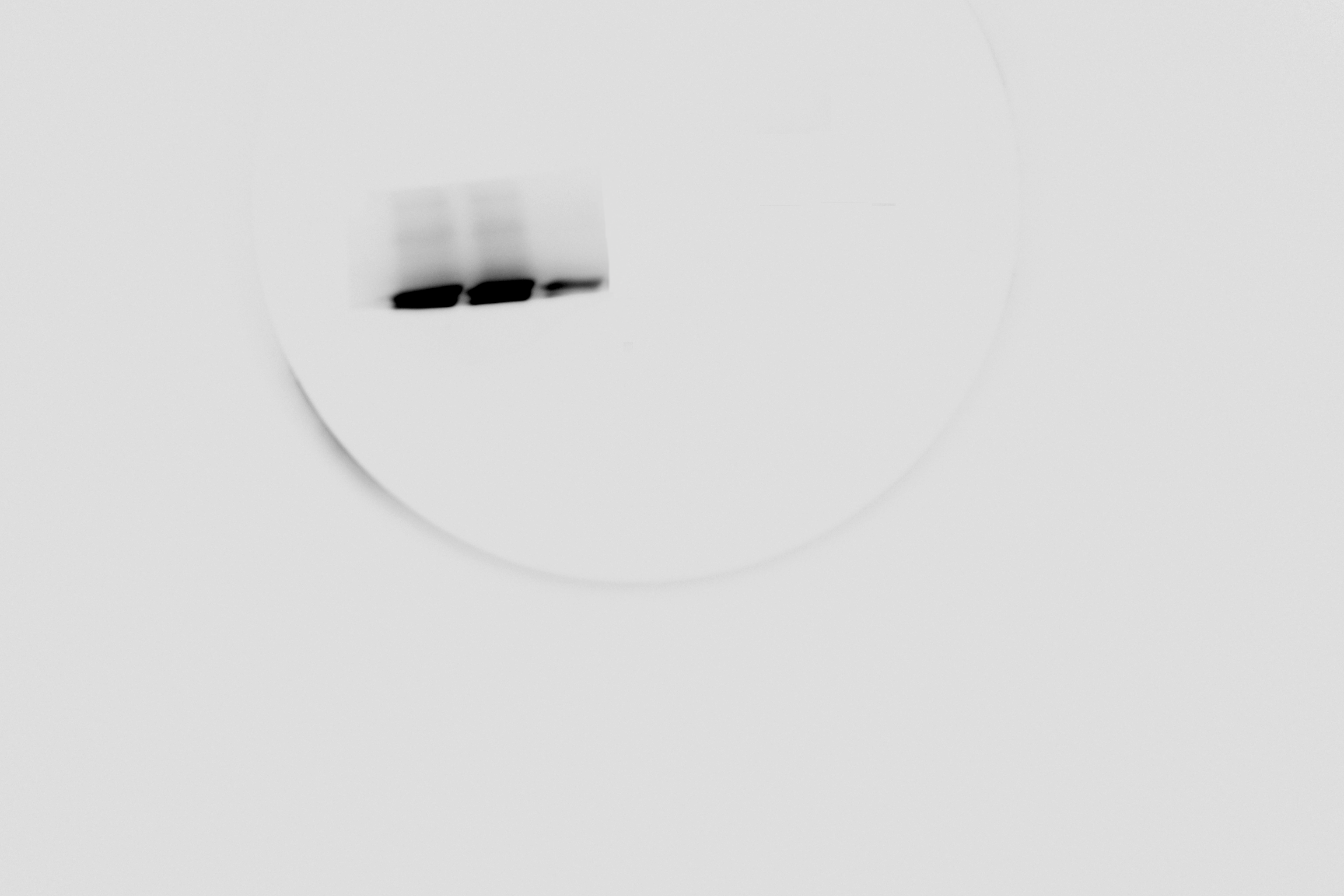

Supplement: S78 Fig — (TIF) [file pone.0153919.s078.tif]

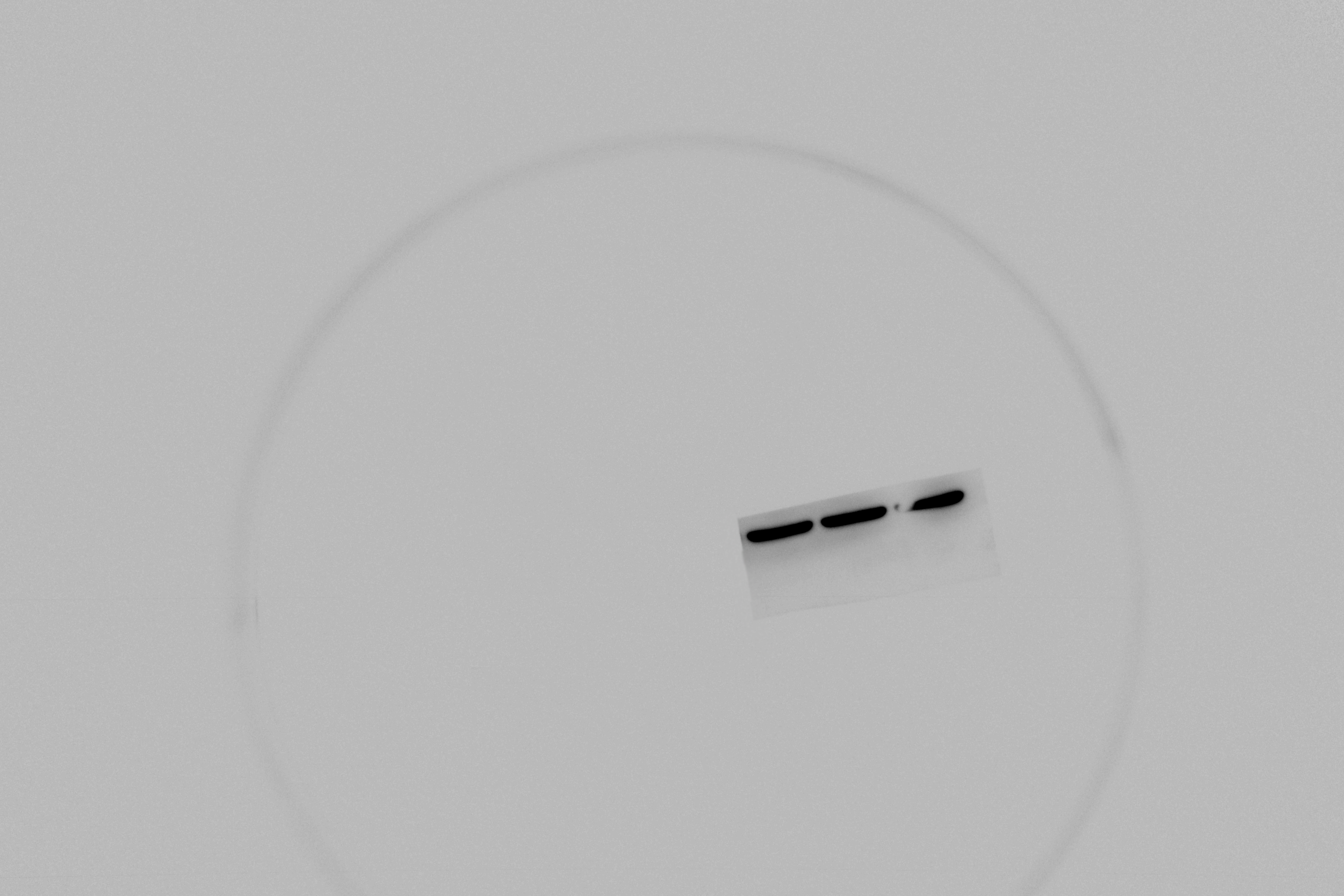

Supplement: S79 Fig — (TIF) [file pone.0153919.s079.tif]

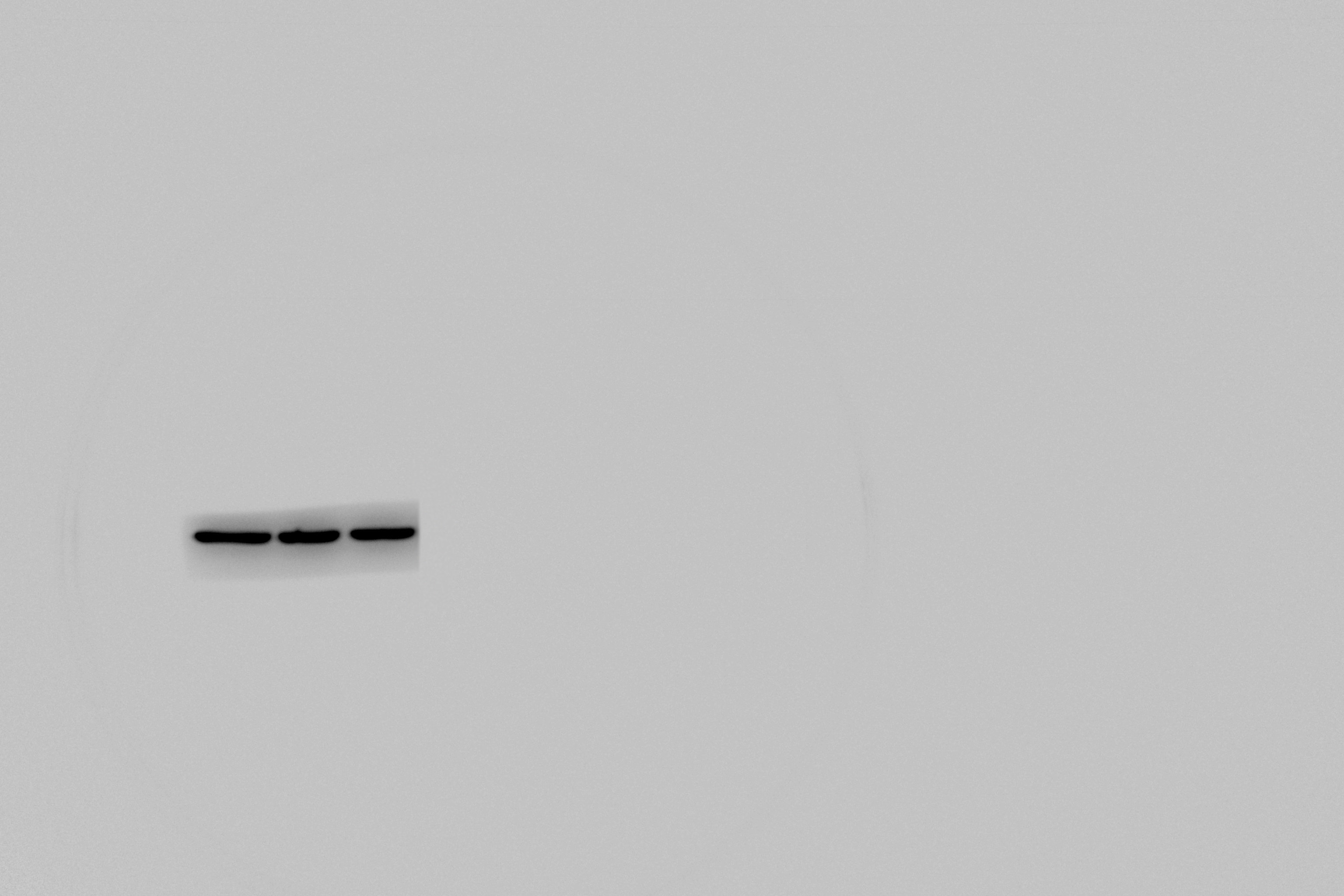

Supplement: S81 Fig — (TIF) [file pone.0153919.s081.tif]

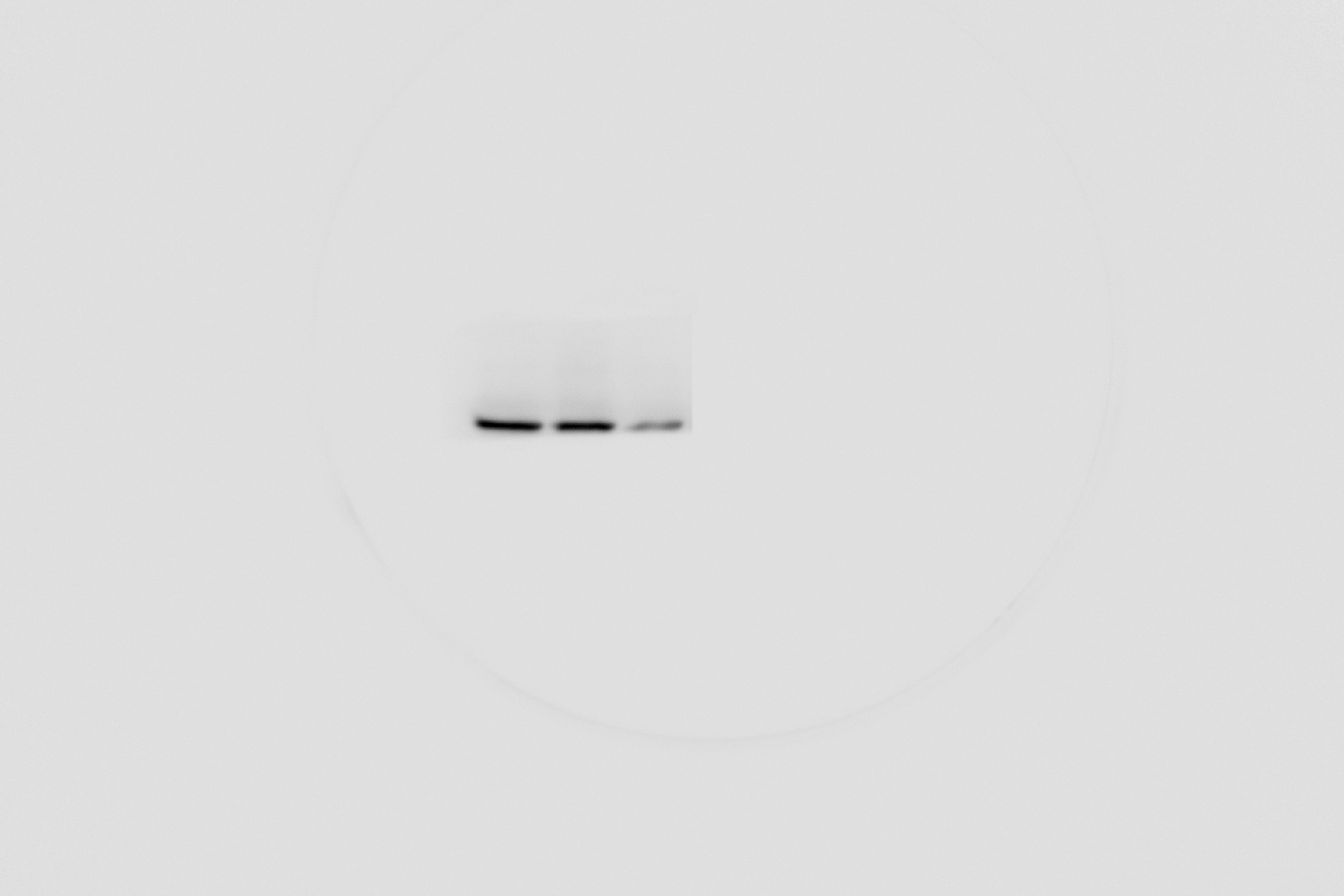

Supplement: S82 Fig — (TIF) [file pone.0153919.s082.tif]

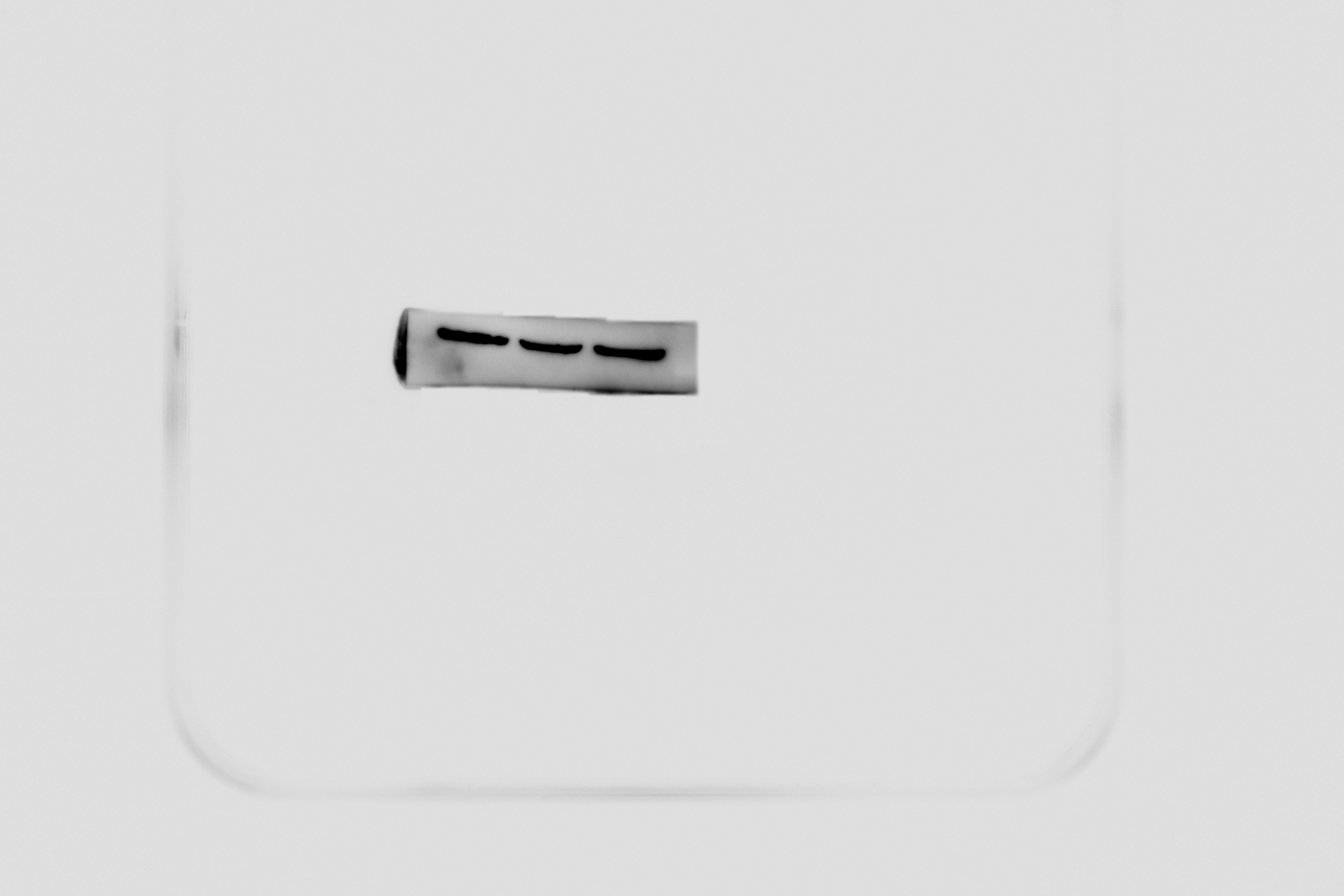

Supplement: S83 Fig — (TIF) [file pone.0153919.s083.tif]

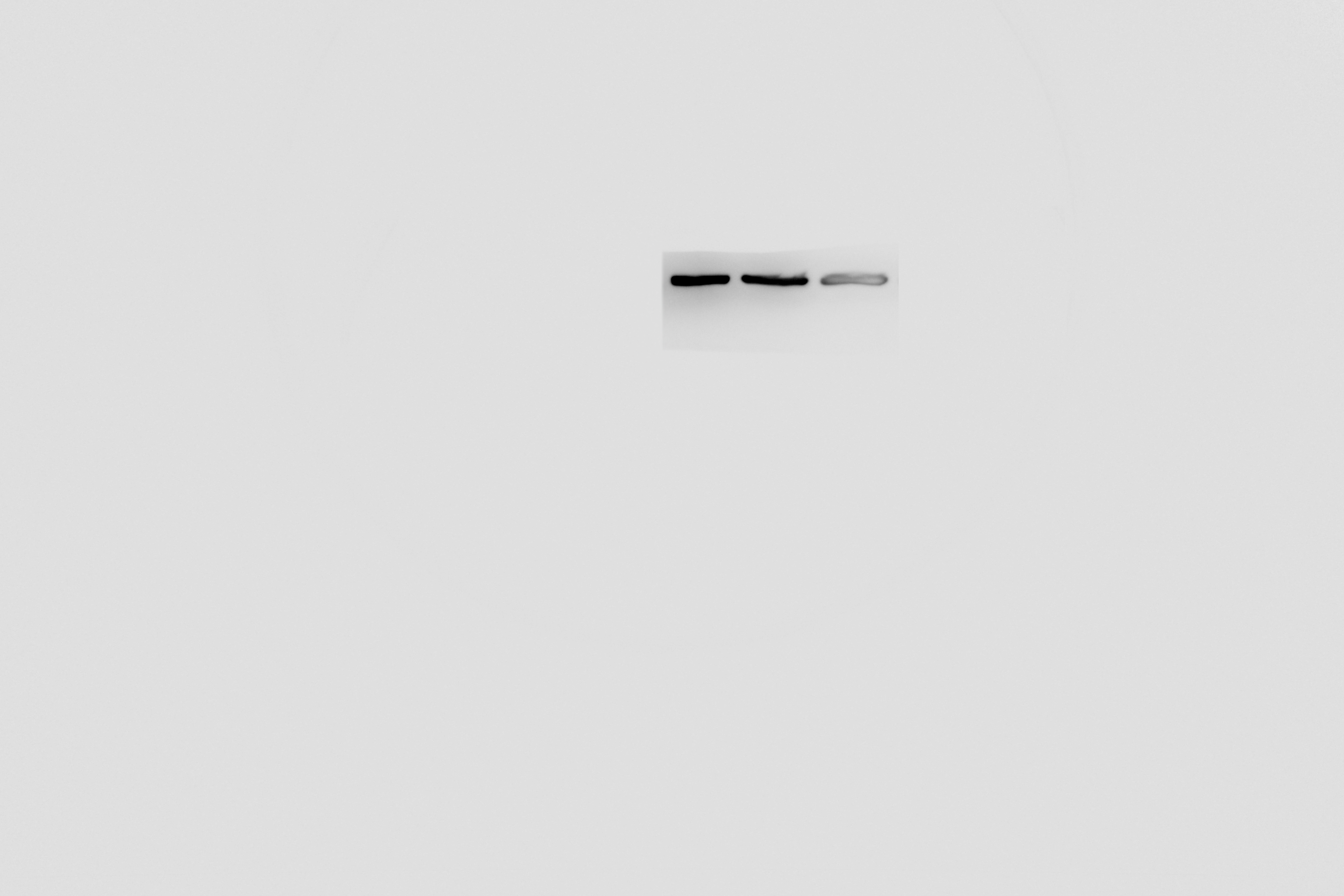

Supplement: S84 Fig — (TIF) [file pone.0153919.s084.tif]

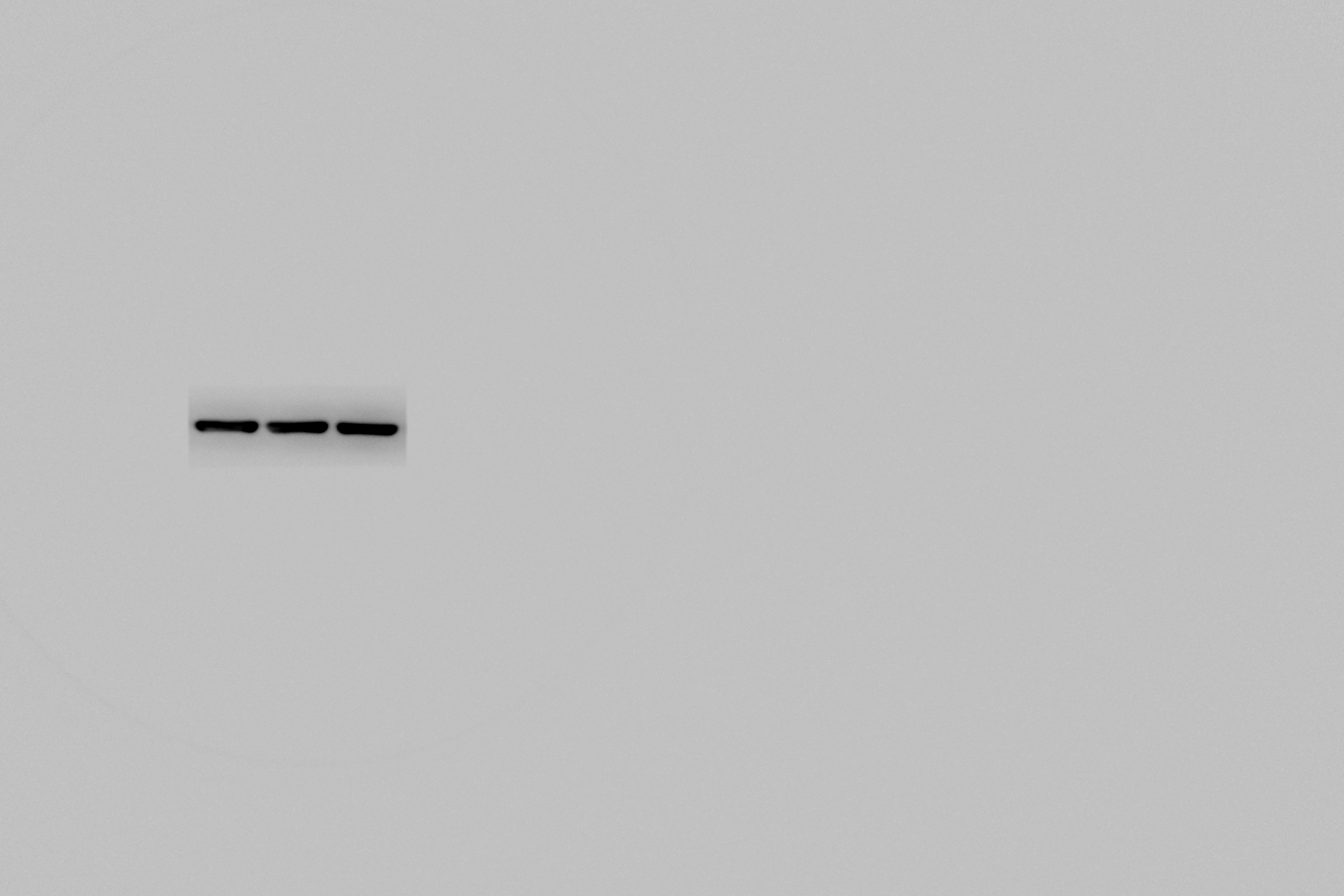

Supplement: S85 Fig — (TIF) [file pone.0153919.s085.tif]

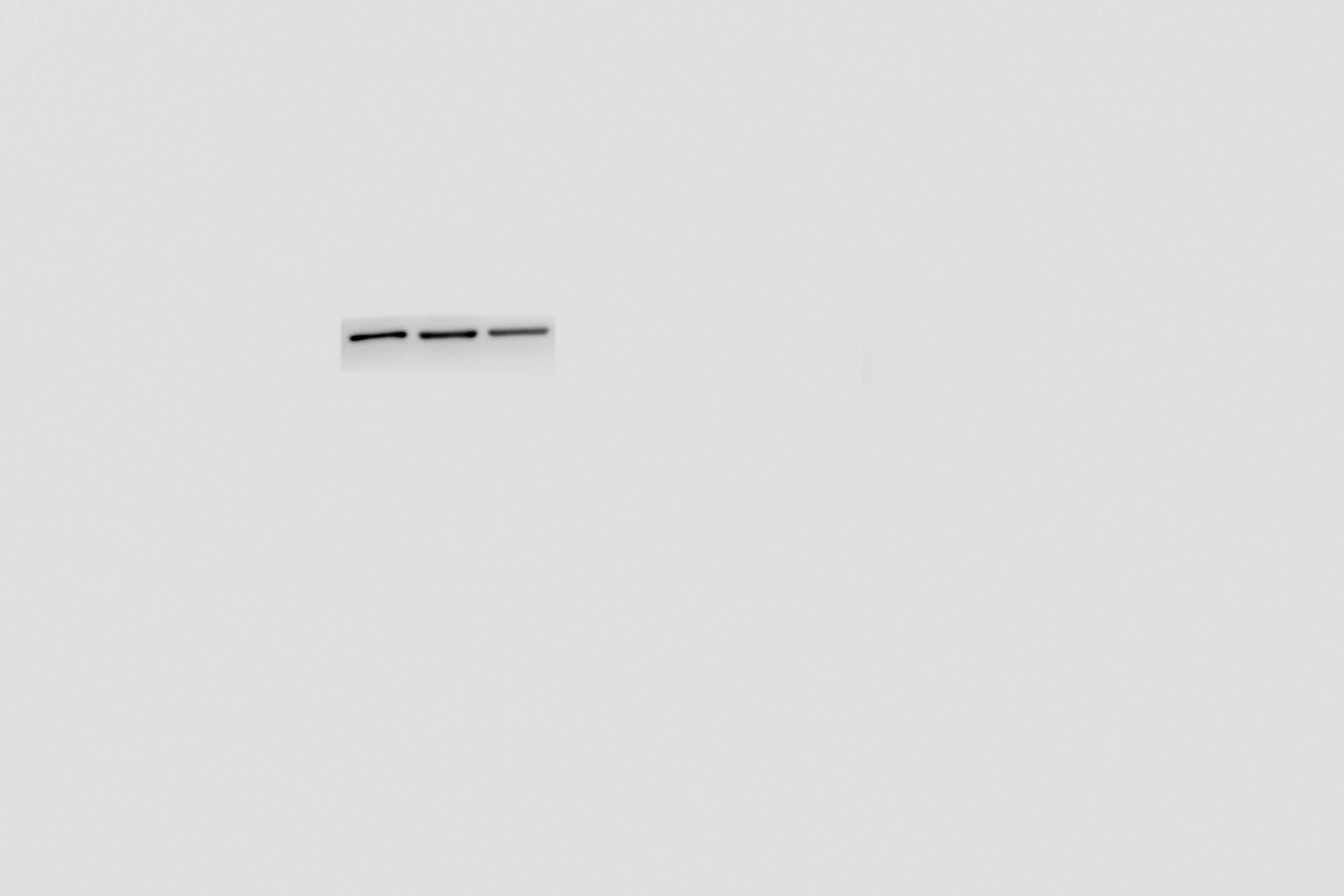

Supplement: S86 Fig — (TIF) [file pone.0153919.s086.tif]

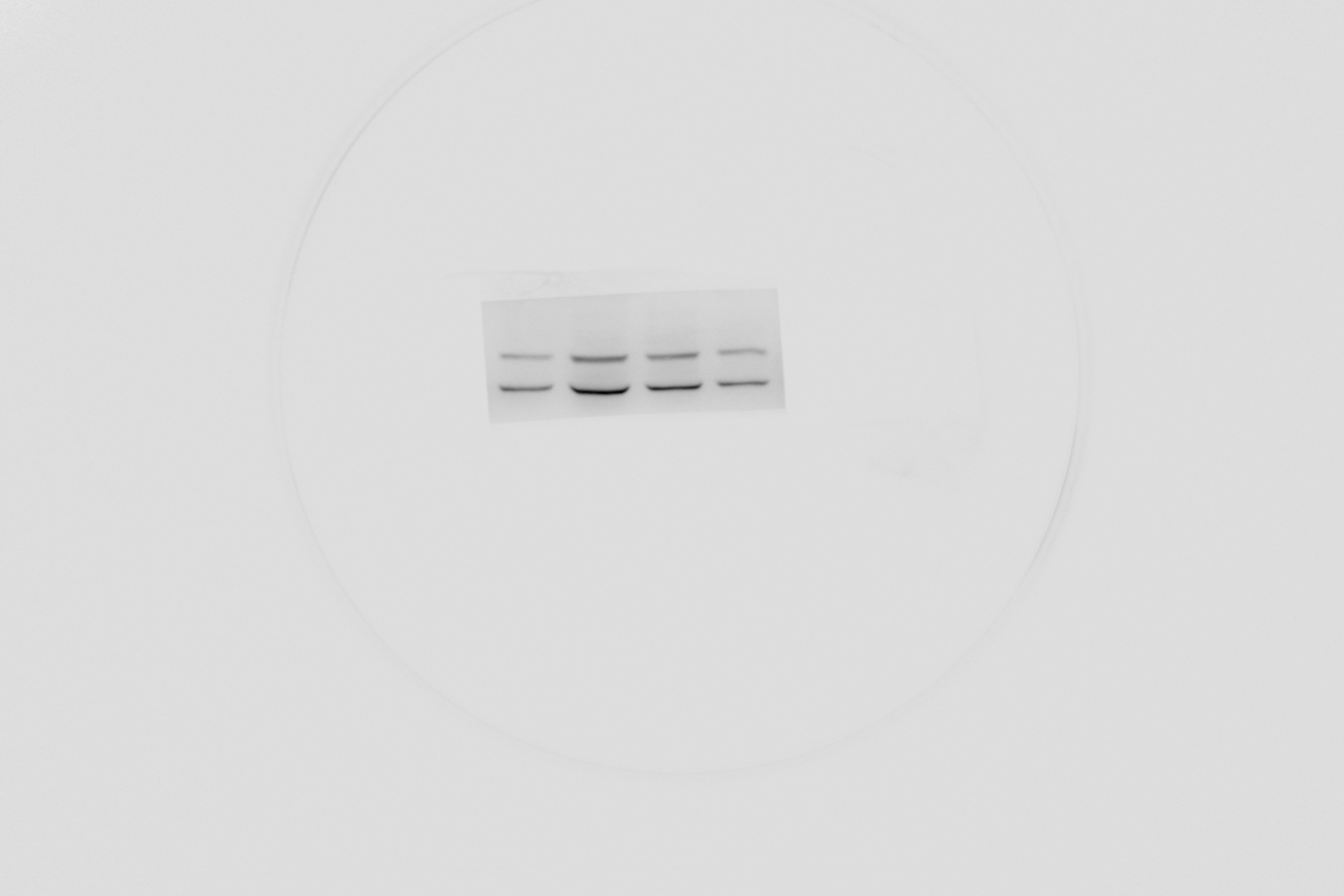

Supplement: S91 Fig — (TIF) [file pone.0153919.s091.tif]

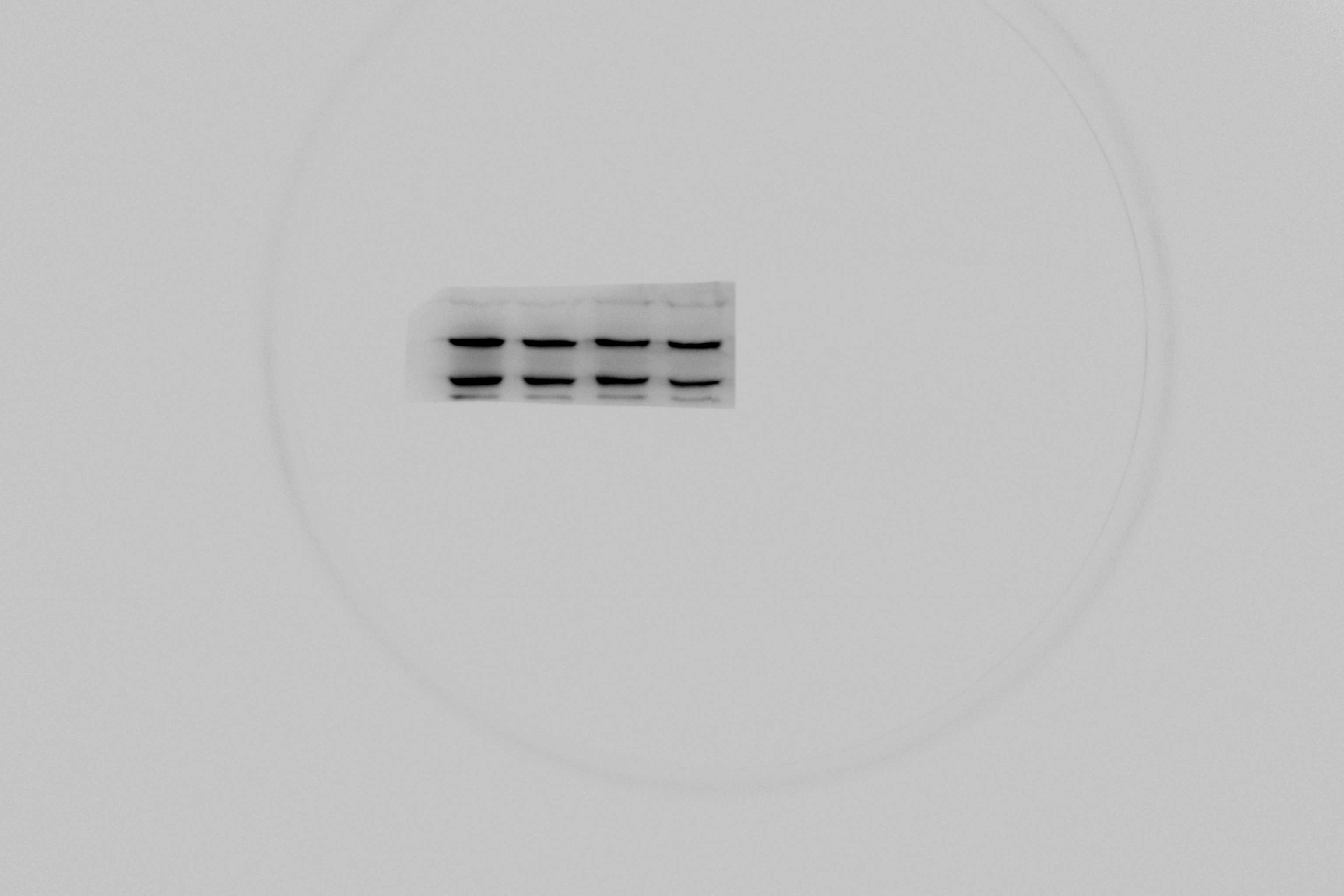

Supplement: S92 Fig — (TIF) [file pone.0153919.s092.tif]

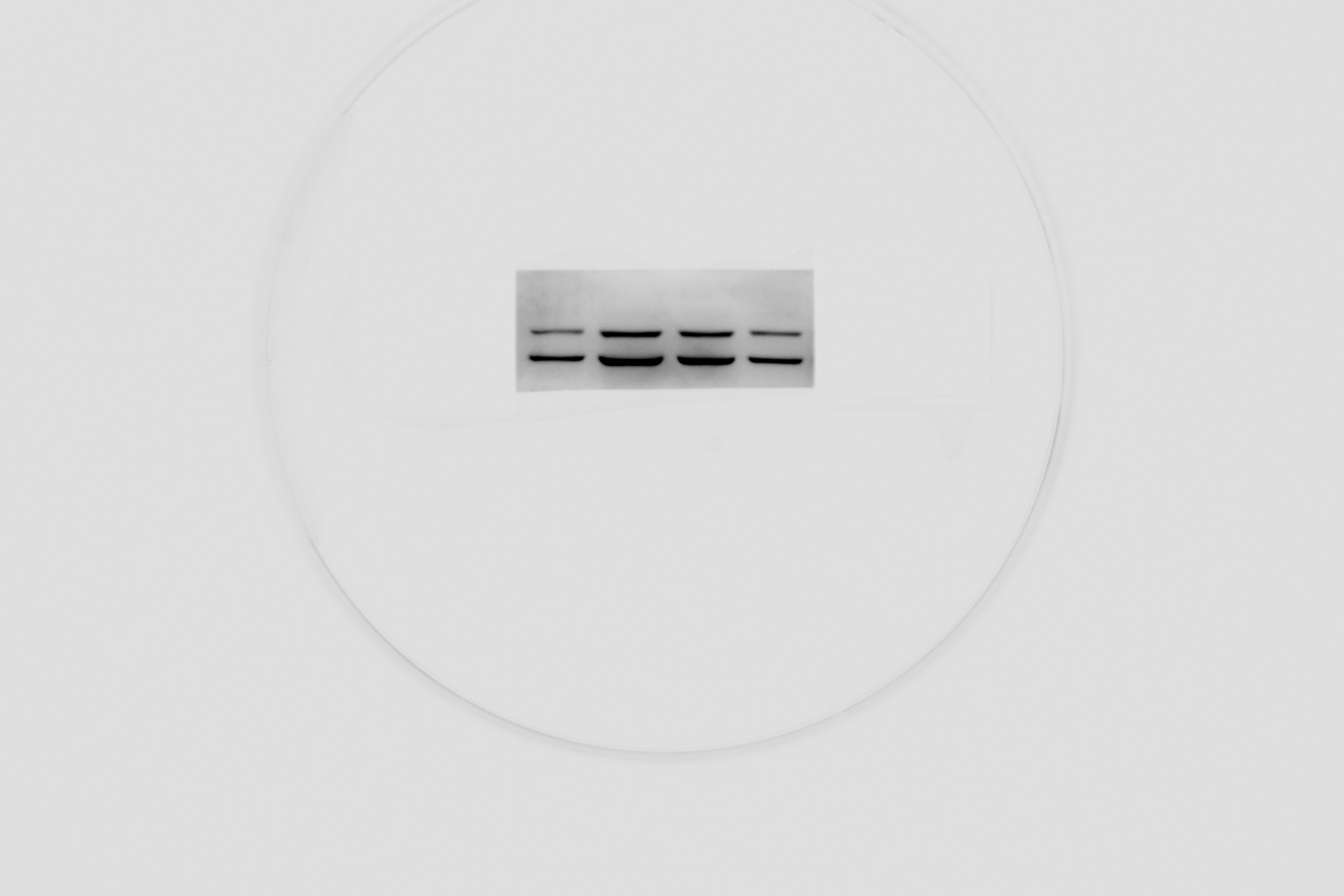

Supplement: S93 Fig — (TIF) [file pone.0153919.s093.tif]

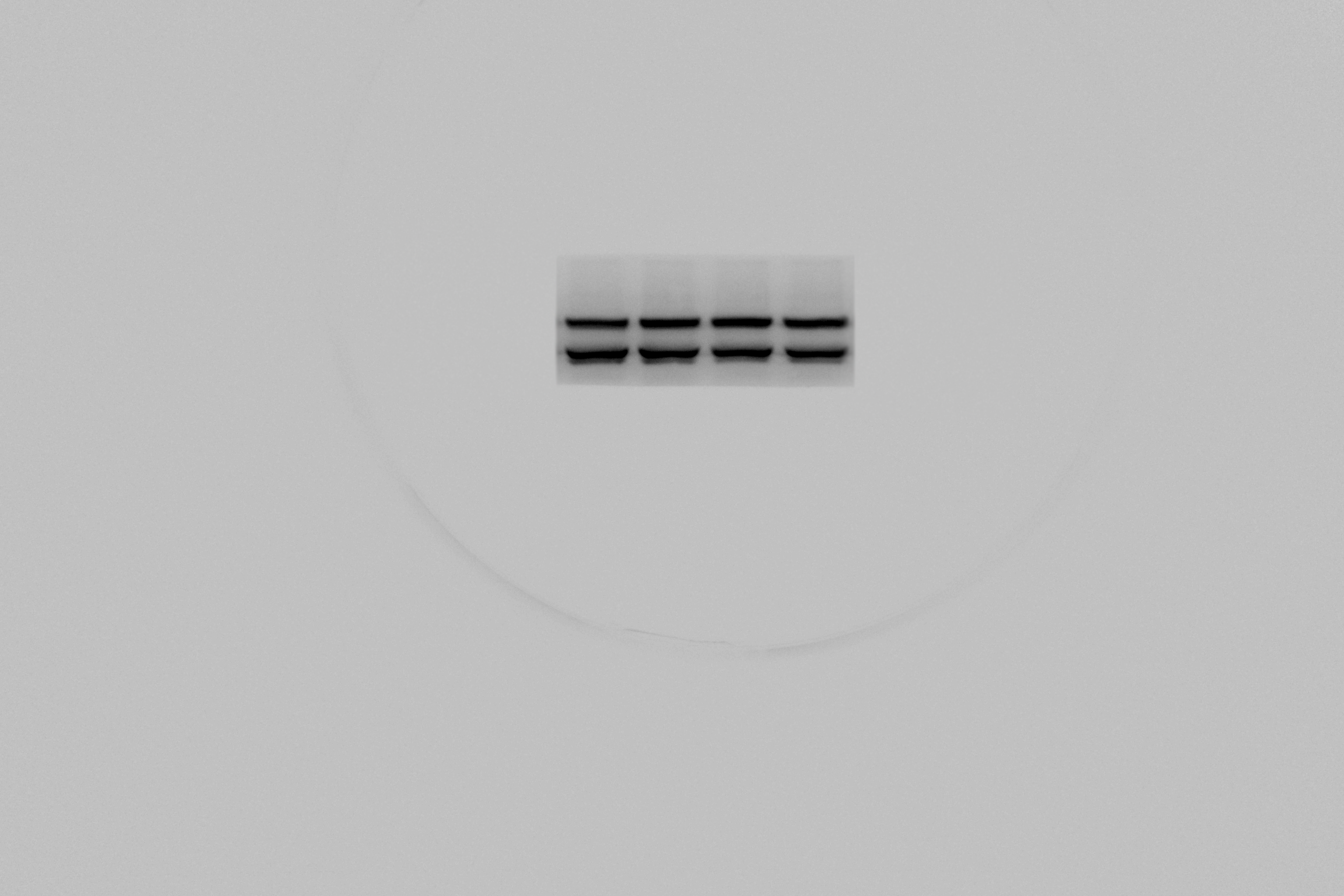

Supplement: S94 Fig — (TIF) [file pone.0153919.s094.tif]

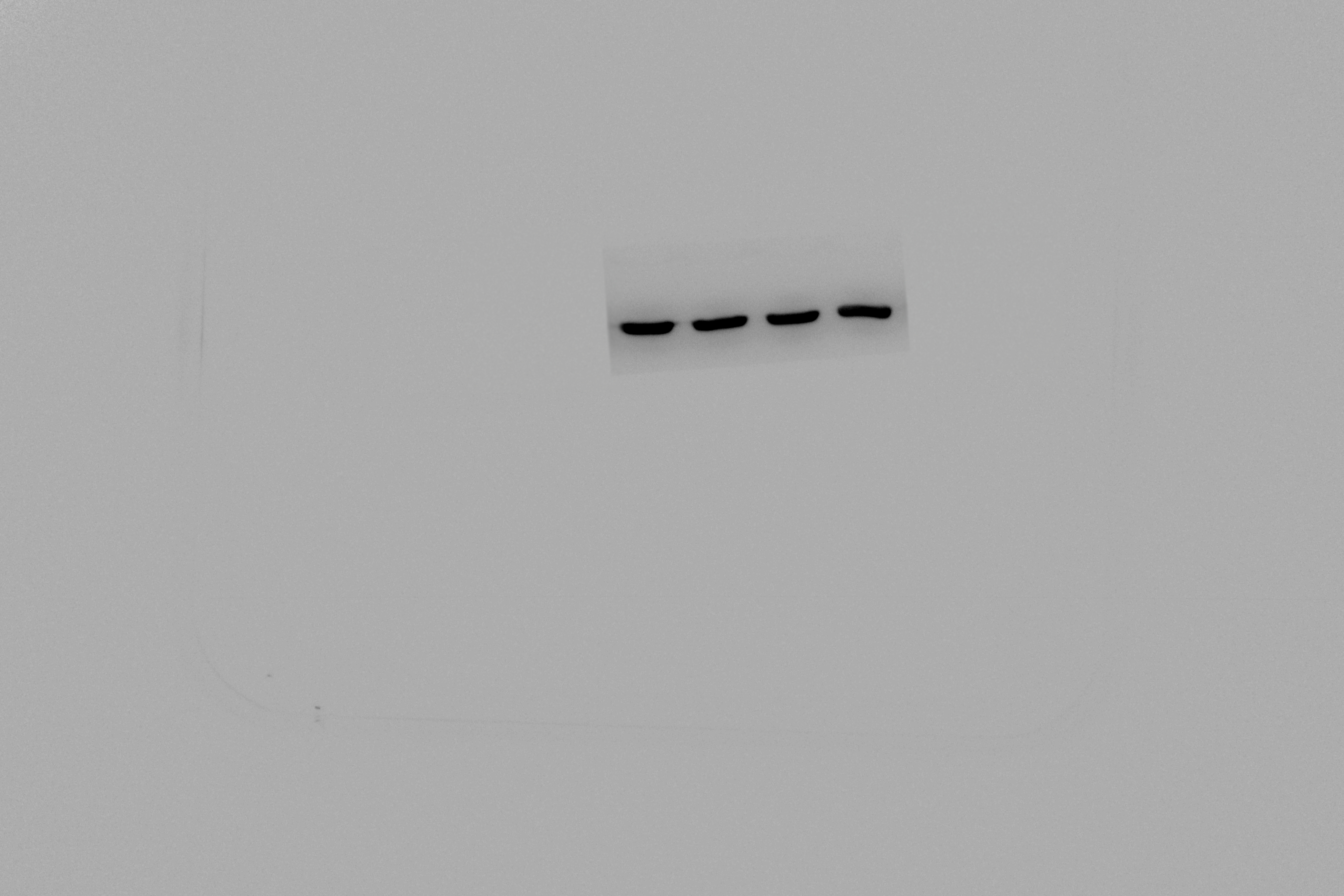

Supplement: S95 Fig — (TIF) [file pone.0153919.s095.tif]

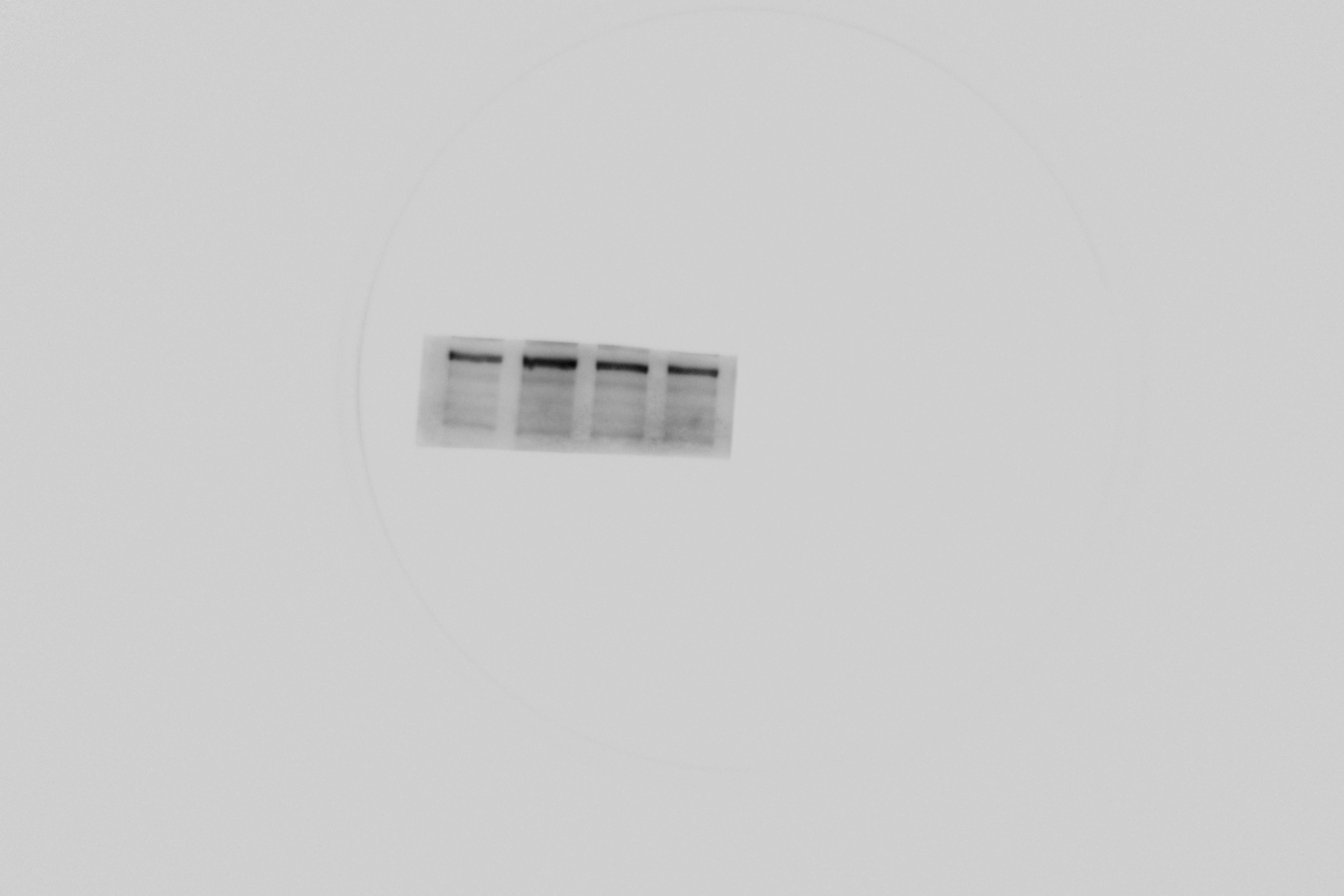

Supplement: S96 Fig — (TIF) [file pone.0153919.s096.tif]

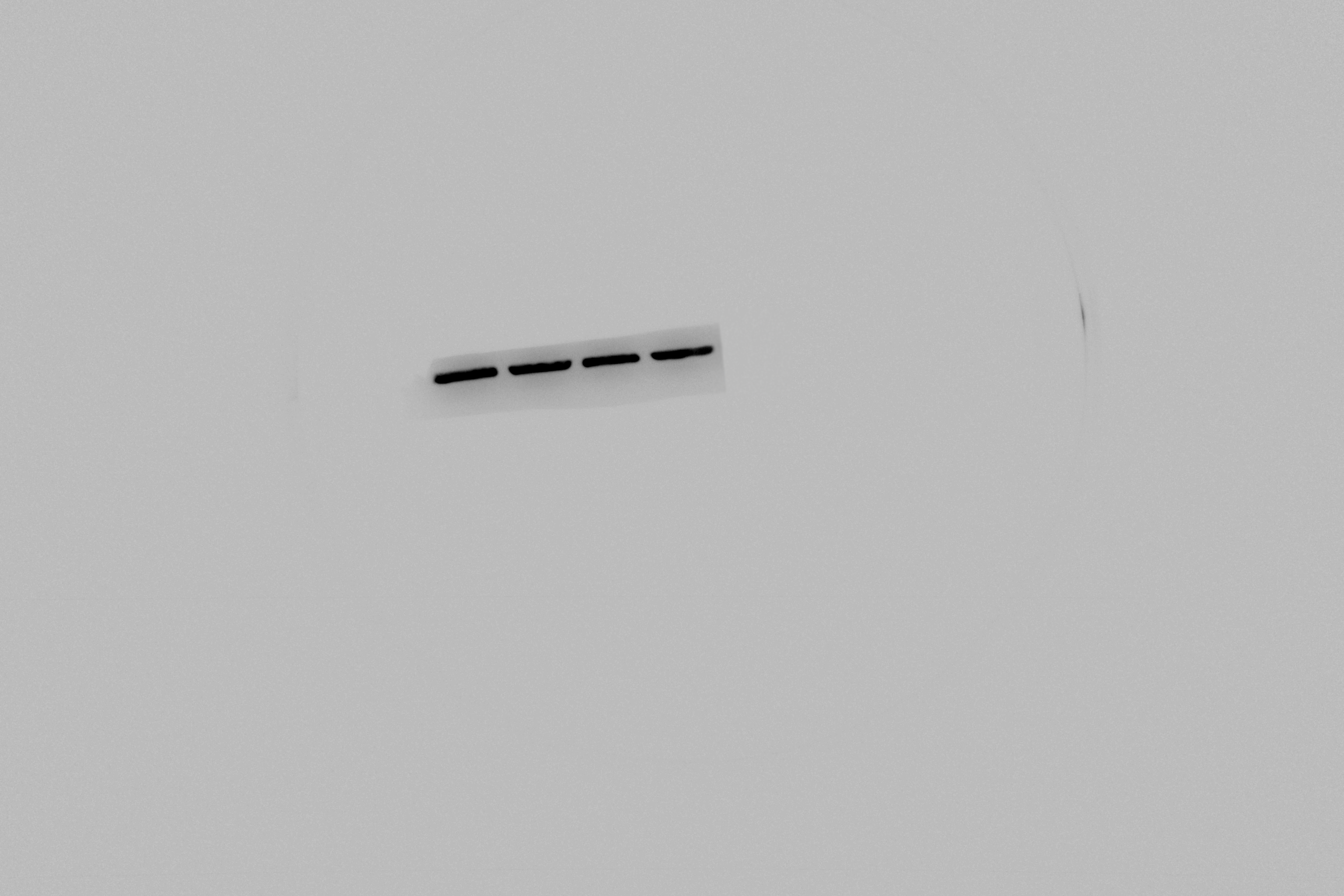

Supplement: S97 Fig — (TIF) [file pone.0153919.s097.tif]

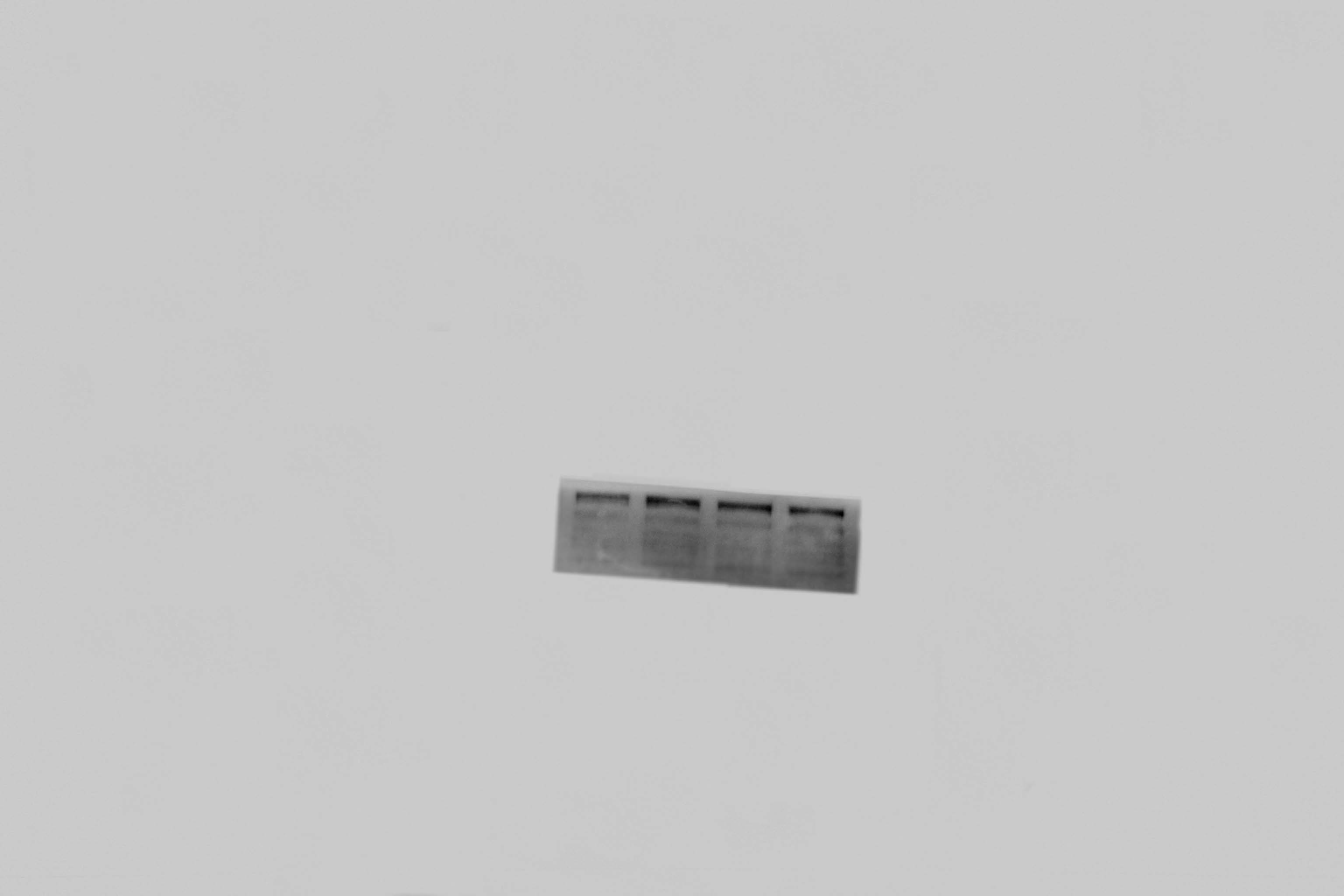

Supplement: S98 Fig — (TIF) [file pone.0153919.s098.tif]

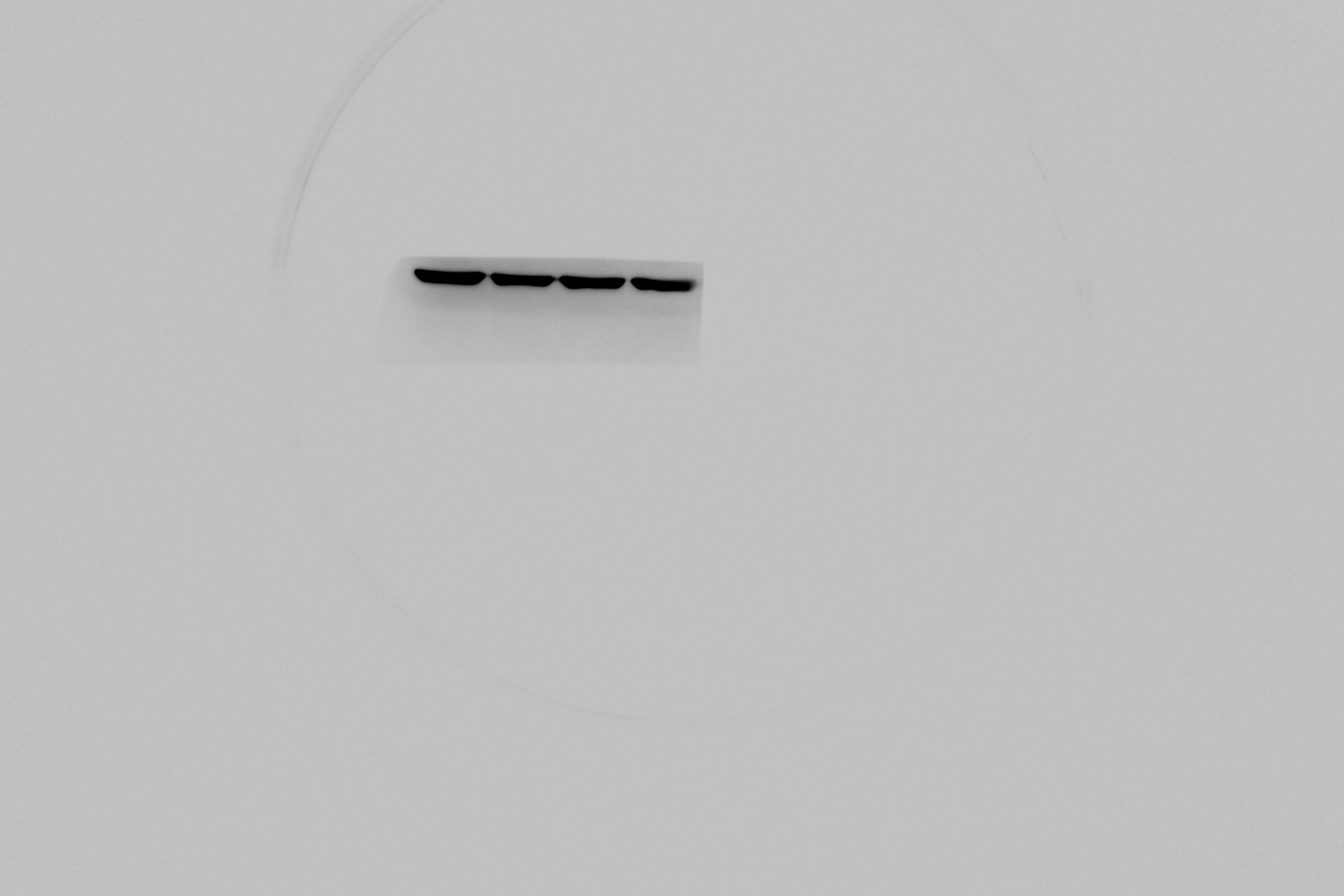

Supplement: S99 Fig — (TIF) [file pone.0153919.s099.tif]

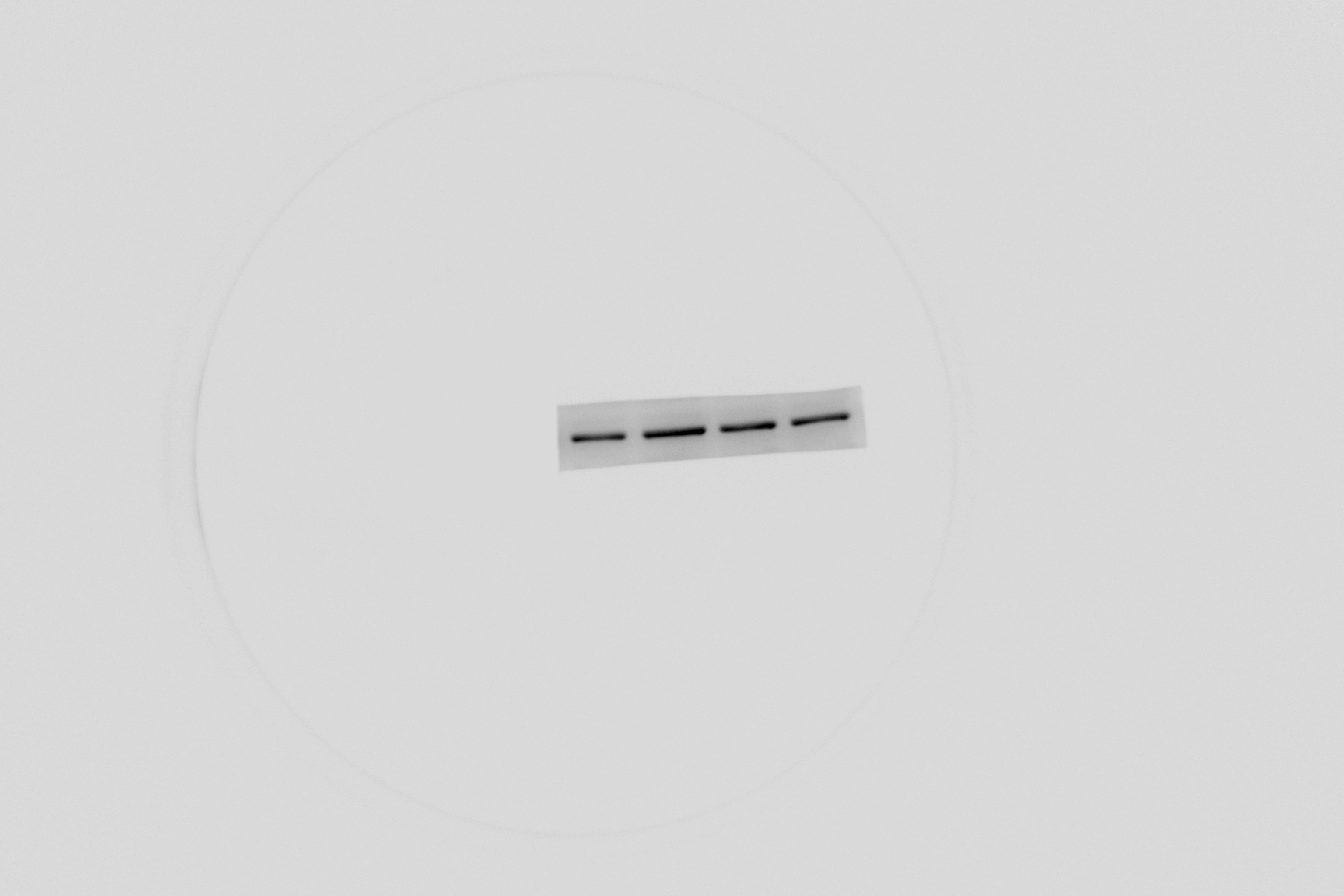

Supplement: S100 Fig — (TIF) [file pone.0153919.s100.tif]

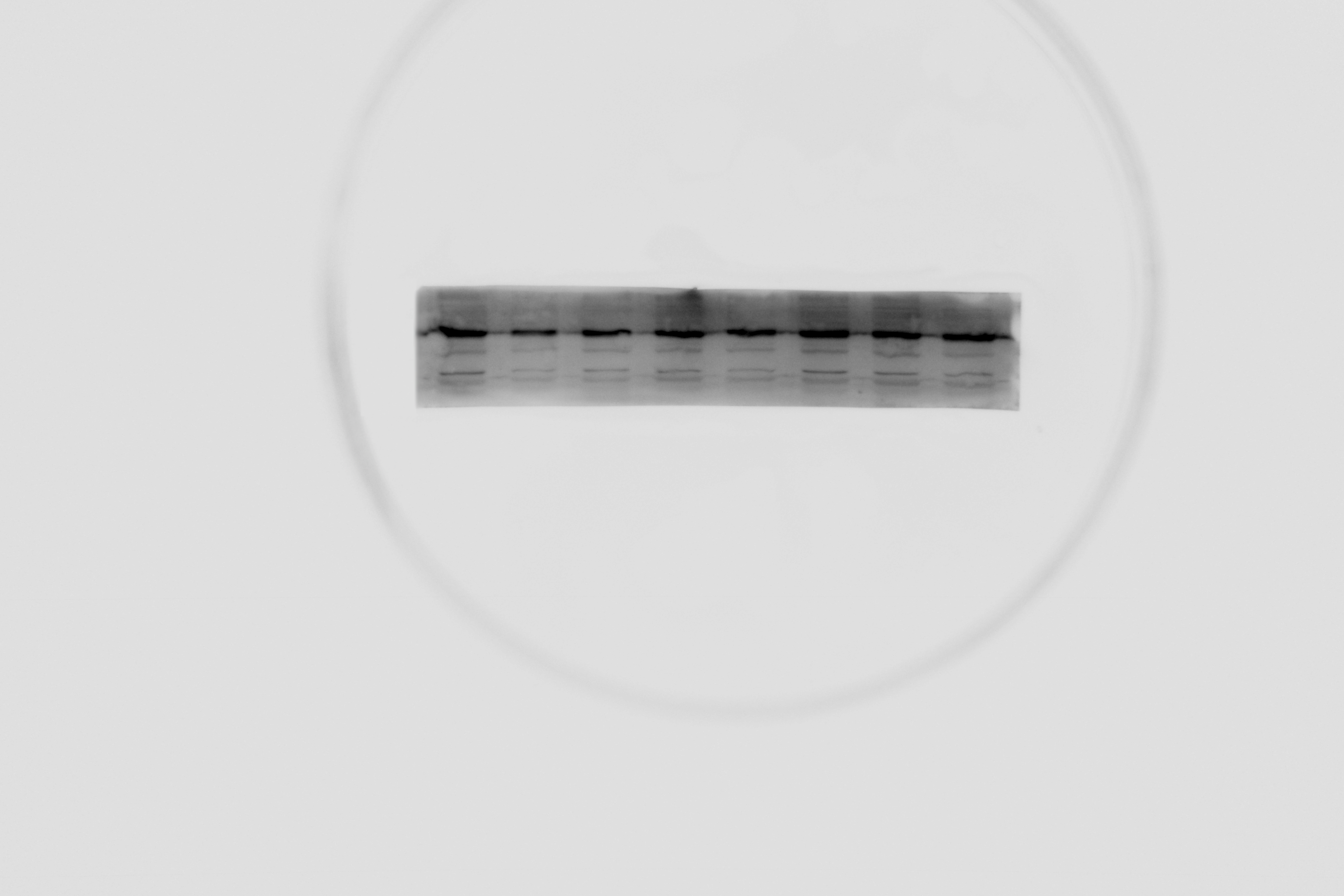

Supplement: S102 Fig — (TIF) [file pone.0153919.s102.tif]

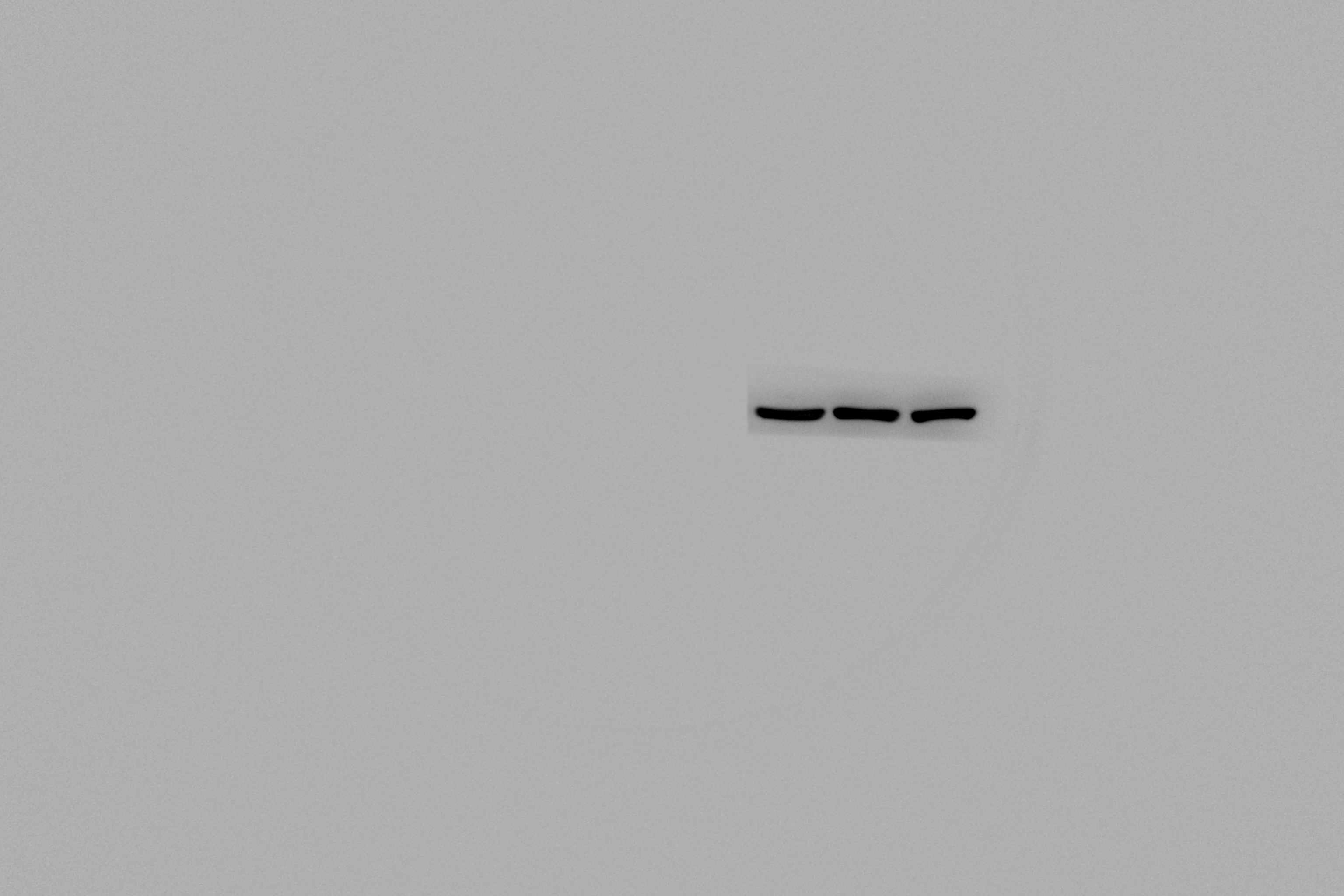

Supplement: S103 Fig — (TIF) [file pone.0153919.s103.tif]

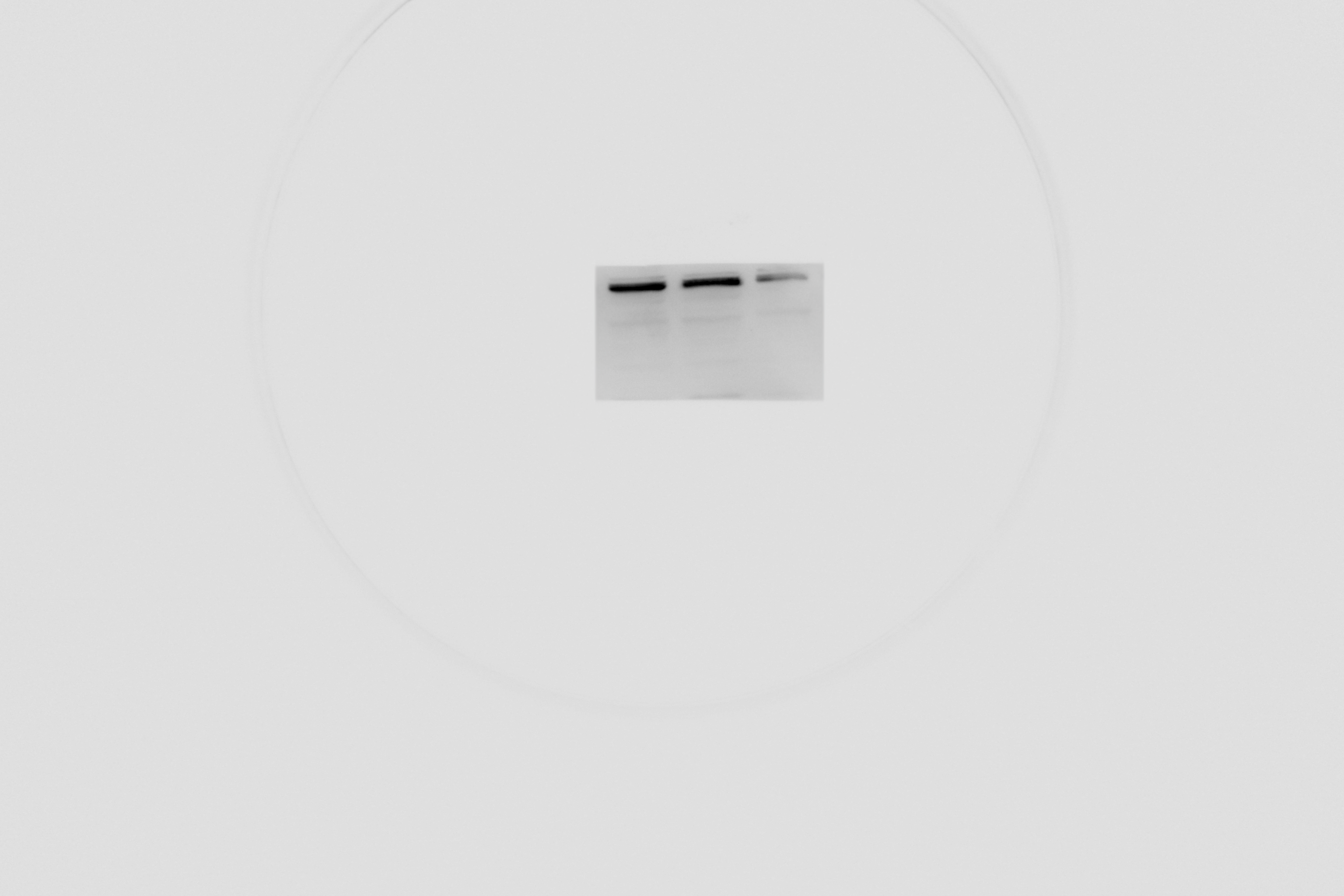

Supplement: S104 Fig — (TIF) [file pone.0153919.s104.tif]

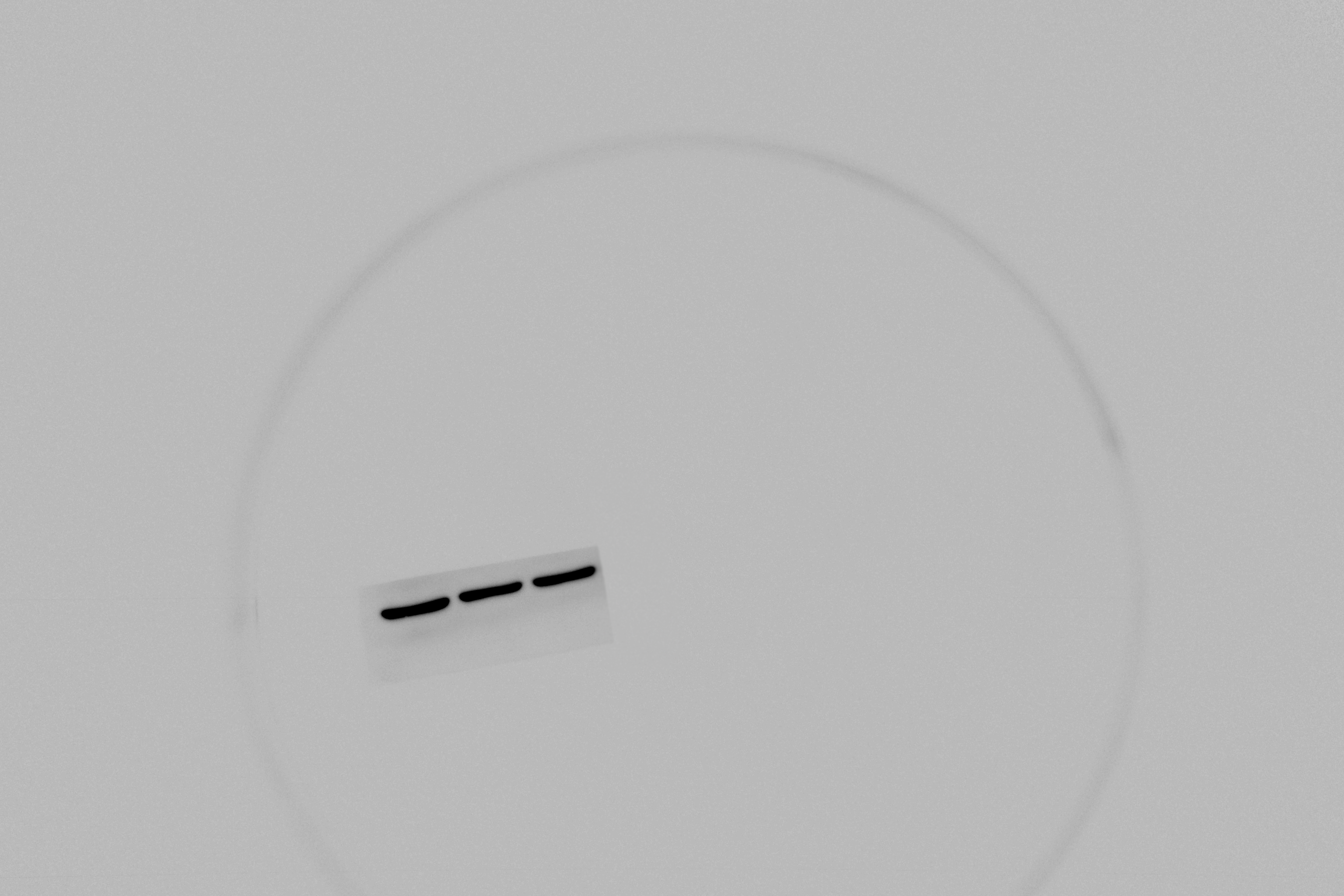

Supplement: S105 Fig — (TIF) [file pone.0153919.s105.tif]

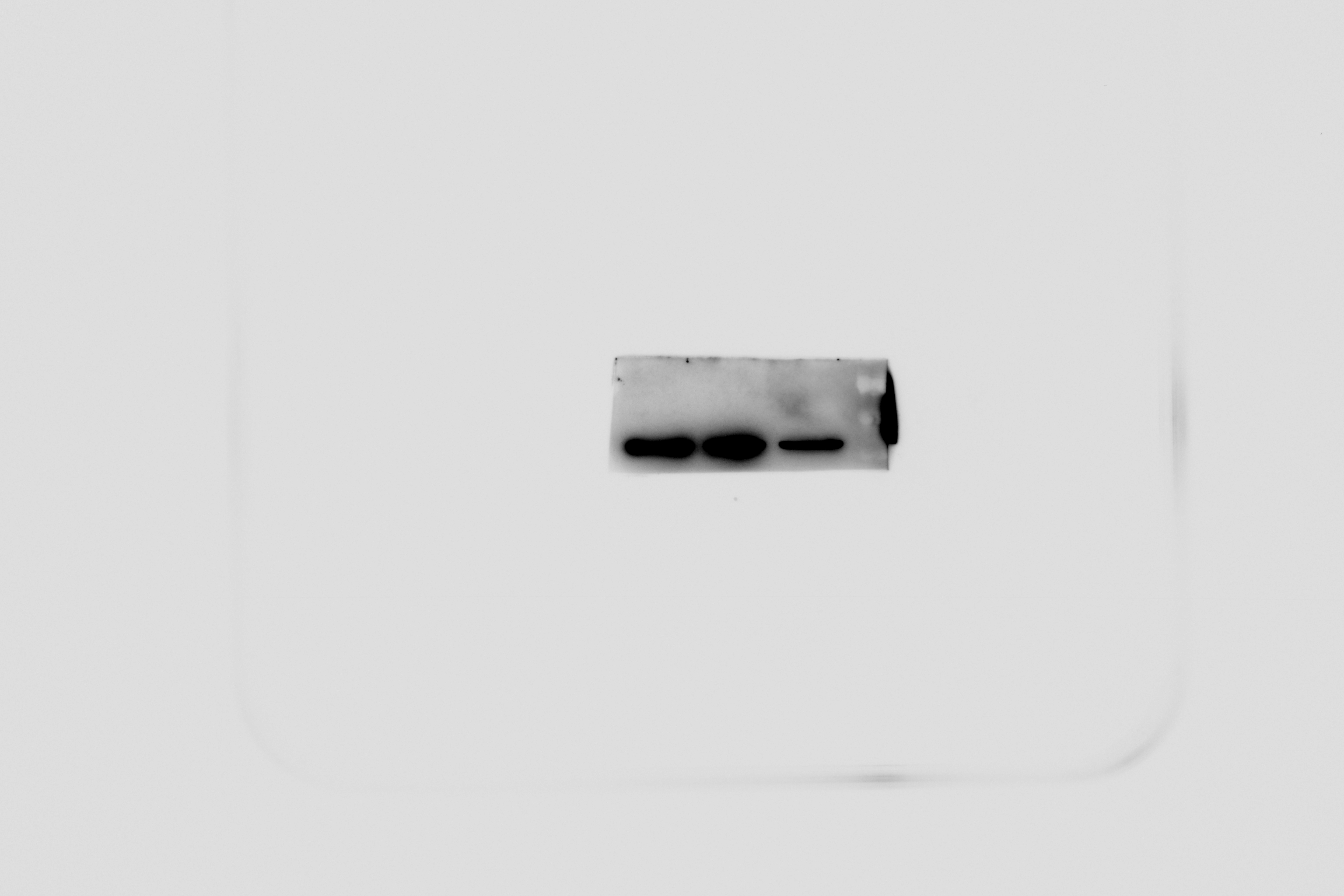

Supplement: S106 Fig — (TIF) [file pone.0153919.s106.tif]

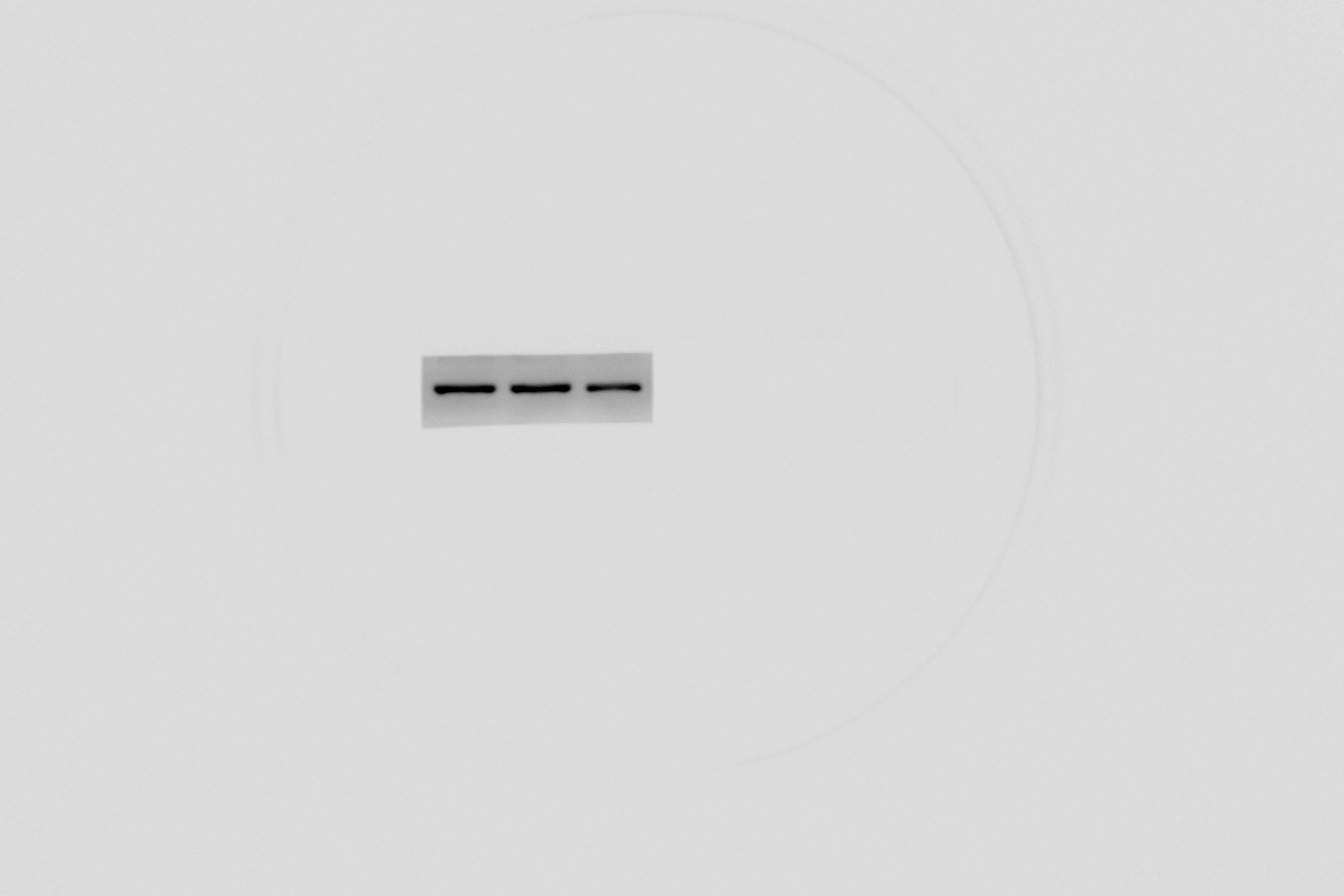

Supplement: S108 Fig — (TIF) [file pone.0153919.s108.tif]
